# Metaproteomics reveals major microbial players and their biodegradation functions in a large-scale aerobic composting plant

**DOI:** 10.1111/1751-7915.12290

**Published:** 2015-05-19

**Authors:** Dongming Liu, Mingxiao Li, Beidou Xi, Yue Zhao, Zimin Wei, Caihong Song, Chaowei Zhu

**Affiliations:** 1 College of Life Sciences, Northeast Agricultural UniversityHarbin, 150030, China; 2 Innovation Base of Groundwater and Environmental System Engineering, Chinese Research Academy of Environmental ScienceBeijing, 100012, China

## Abstract

Composting is an appropriate management alternative for municipal solid waste; however, our knowledge about the microbial regulation of this process is still scare. We employed metaproteomics to elucidate the main biodegradation pathways in municipal solid waste composting system across the main phases in a large-scale composting plant. The investigation of microbial succession revealed that Bacillales, Actinobacteria and Saccharomyces increased significantly with respect to abundance in composting process. The key microbiologic population for cellulose degradation in different composting stages was different. Fungi were found to be the main producers of cellulase in earlier phase. However, the cellulolytic fungal communities were gradually replaced by a purely bacterial one in active phase, which did not support the concept that the thermophilic fungi are active through the thermophilic phase. The effective decomposition of cellulose required the synergy between bacteria and fungi in the curing phase.

## Introduction

Composting is an aerobic process, during which organic waste is biologically degraded by microorganisms to humus-like material (Partanen *et al*., 2010). The products have a commercial value as soil conditioner or organic fertilizer (Negro *et al*., 1999). The typical composting batch could be divided into three phases: the mesophilic phase (10–42°C), the thermophilic phase (45–70°C) and the cooling and maturation phase (65–23°C) (Joséphine and Philippe, 2004; Bonito *et al*., 2010). Both bacteria and fungi play a key role in a typical composting process. Thus, a thorough understanding of microbial communities throughout the composting process is crucial for understanding the system and optimizing compost product quality (De Gannes *et al*., 2013a).

The information of microbial communities in composts has been examined by culture-independent deoxyribonucleic acid (DNA)-based methods. These investigations have focused on analysis of shifts in microbial community composition via amplified ribosomal DNA restriction analysis, single strand-conformation polymorphism, restriction fragment length polymorphism, denaturing gradient gel electrophoresis and nucleic acid microarrays (Peters *et al*., 2000; Dees and Ghiorse, 2001; Franke-Whittle *et al*., 2009; 2014; Baharuddin *et al*., 2010; Bonito *et al*., 2010; Partanen *et al*., 2010; Charbonneau *et al*., 2012; Tian *et al*., 2013). However, little is known about global gene expression of compost as relevant studies have not been performed.

Proteins reflect the actual functionality with respect to metabolic reactions and regulatory cascades, and give more direct information about microbial activity than functional genes and even the corresponding messenger RNAs (Wilmes and Bond, 2006). Furthermore, the use of proteins also bears the potential to reveal the identity of the active microorganisms via database analysis using the level of homology to other species (Benndorf *et al*., 2007). Thus, the presence of specific proteins in environmental samples is a potentially reliable indicator for microbial function (Benndorf *et al*., 2007). Wilmes and Bond (2004) proposed the term ‘metaproteomics’ for the large-scale characterization of the entire protein complement of environmental microbiota at a given point in time. By determining proteins which have been synthesized by microorganisms present at the time of sampling, metaproteomics enables the reconstruction of microbial processes and metabolic pathways that are central to the functioning of the ecosystem (Williams *et al*., 2012). Until now, several authors presented metaproteome data from different environmental systems, such as soil (Benndorf *et al*., 2007; Chourey *et al*., 2010), activated sludge (Wilmes and Bond, 2006), water (Benndorf *et al*., 2007; Lauro *et al*., 2011; Williams *et al*., 2012) and leaf litter (Schneider *et al*., 2012). Additionally, metaproteomics was used to define key catabolic players at contaminated sites to predict pollutant degradation networks in the environment (Guazzaroni *et al*., 2012). Metaproteomics has become an efficient tool to unravel and characterize metabolic networks as well as ecological interactions during complex environments.

For economical and capacity reasons, there is always a tendency to push the capacity limits and minimize the retention time at composting plants (Partanen *et al*., 2010). This may lead to unwanted anaerobic conditions and failure to attain the temperatures required for hygienization. To adjust these conditions at the large-scale composting plants, comprehensive information is needed regarding total biodiversity and metabolic processes. In the current study, we analysed the metaproteome from composting samples collected in different phases in a large-scale composting plant. By examining expressed proteins in the samples, we were able to infer the predominant metabolic processes performed by bacteria and fungi present in composting pile, and consider the significance of the differences between main composting phases.

## Results and discussion

### Physicochemical data for the composting samples

The composting pile achieved thermophilic temperature shortly after pile establishment. This temperature was maintained for 16 days, and gradually descended to ambient values thereafter. The pH values of the composting pile increased from acidic values in earlier phase to alkaline values on day 50 (Table 1). The highest water content level was seen in the active phase, with approximately 30% water content loss occurring afterwards, which might be explained by microbial heat generation causing enhanced desiccation. The total organic carbon (C) content reductions were found in composting pile, reflecting a notable mineralization of organic matter over time. The total nitrogen (N) percentage showed an increasing trend with composting duration, which is due to the concentration effect caused by carbon loss associated with mineralization of the organic matter (Shemekite *et al*., 2014). The C/N ratio decreased significantly over time, as C was lost in the form of CO_2_ through microbial respiration and N was recycled (Ryckeboer *et al*., 2003).

**Table 1:** Physical and chemical properties of the samples

	LS1	LS2	LS3
Age (d)	5	20	50
Temperature (°C)	60.3	62.4	47.0
pH	6.46	6.90	7.46
Water content (%)	58.80	53.02	34.84
Organic C (%)	63.71	52.29	47.17
N (%)	1.70	1.77	1.95
C : N ratio	37.48	29.54	24.19

### Bacterial diversity represented in the composting metaproteome

A total of 640 proteins were detected (222 from the sample LS1, 220 from the sample LS2 and 198 from the sample LS3). Of the 640 proteins, 449 were with highest sequence identity to bacterial proteins and 191 fungal proteins. Within the bacterial subset, the majority had the best match (highest sequence identity) to proteins from members of the *Gammaproteobacteria* (181), followed by Bacilli (65), *Alphaproteobacteria* (41), Actinobacteria (37) and *Betaproteobacteria* (31) (Table 2). Within the *Gammaproteobacteria*, most of the matches had highest identity to proteins from the order Pseudomonadales (61) and Enterobacteriales (50). Within Pseudomonadales, the genus Azotobacter (5) can carry out the denitrification process (Szántó, 2009). The order Enterobacteriales includes the genus Salmonella, most of which are pathogens, and Escherichia, which also includes pathogenic strains (Sundberg *et al*., 2011). Within the *Alphaproteobacteria*, most of the proteins had the best match to members of three groups: order Rhizobiales (22), Rhodobacterales (7) and Rhodospirillales (6). The Rhizobium is the nitrogen-fixing bacteria; they take nitrogen from the air and convert it into ammonia, a form of nitrogen that plants can use. Within Rhodobacterales, some are denitrifying bacteria such as *Paracoccus denitrificans* (Baumann *et al*., 1996; Siddavattam *et al*., 2011). The order Rhodospirillales includes nitrogen-fixing bacteria such as Rhodobacter sphaeroides (Kontur *et al*., 2012). In the groups related to *Betaproteobacteria*, most of the matches had highest identity to proteins from the order Burkholderiales (17), Methylophilales (5), Neisseriales (4) and Nitrosomonadales (3). Cupriavidus and Bordetella were the most dominant genuses among the order Burkholderiales. Cupriavidus is adapted to several form of heavy metal stress (Nies, 1999; 2000). Bordetella is best known for species that are opportunistic human pathogens, but is also identified as a soil bacterium (Eriksson *et al*., 2003). The order Nitrosomonadales was found as nitrifying bacteria (Szántó, 2009). Pathogens such as Neiseria were also found among the *Betaproteobacteria*.

**Table 2:** Most abundant bacterial order/suborder for the predominant phyla

Taxonomy	Protein number
*Proteobacteria*	
*Gammaproteobacteria*	181
Pseudomonadales	61
Enterobacteriales	50
*Alphaproteobacteria*	41
Rhizobiales	22
Rhodobacterales	7
Rhodospirillales	6
*Betaproteobacteria*	31
Burkholderiales	17
Methylophilales	5
Neisseriales	4
Nitrosomonadales	3
*Deltaproteobacteria*	15
Firmicutes	
Bacilli	65
Bacillales	45
Lactobacillales	20
Clostridia	19
Actinobacteria	37
Corynebacterineae	19
Streptosporangineae	5
Frankineae	4
Tenericutes	13
Cyanobacteria	12
Bacteroidetes	8
Spirochaetes	7
Thermotogae	5
Aquificae	5

The bacteria found in the metaproteome, such as members of the Actinobacteria, Bacilli, *Alphaproteobacteria*, *Betaproteobacteria* and *Gammaproteobacteria*, were typical for municipal solid waste and lignocellulosic materials (bagasse, coffee and rice) compost. Our data are consistent with the phylogenetic diversity obtained by previous gene-based analyses of facility composting, which identified the relative abundance of Actinobacteria, Bacteroidetes, Firmicutes and *Proteobacteria* (Partanen *et al*., 2010; Sundberg *et al*., 2011; De Gannes *et al*., 2013b). Exception included Bacteroidetes, which were minor community constituents in our study.

### Fungal diversity represented in the composting metaproteome

Matches to the fungi were mainly to Saccharomycetes (114), followed by Schizosaccharomycetes (25), Sordariomycetes (16), Eurotiomycetes (16) and Basidiomycota (9) (Table 3). Within the Saccharomycetes, nearly half (51) of the matches had highest identity to proteins from the genus Saccharomyces; other proteins had the best match to members of genus Candida (14), Eremothecium (14) and Kluyveromyces (11). Some of the yeasts genuses are pathogens, such as Candida, which are opportunistic human pathogens (Bonito *et al*., 2010), and Eremothecium (also known as Ashbya), which are plant pathogens (Ashby and Nowell, 1926). Within the Sordariomycetes, most of the proteins had the best match to members of genus Neurospora (8) and Chaetomium (3), which relate to the degradation of cellulose (Umikalsom *et al*., 1997; Phillips *et al*., 2011; Sygmund *et al*., 2012). Matches to the Eurotiomycetes were mainly to Aspergillus (13), which includes pathogenic strains (Dehghani *et al*., 2012). Basidiomycetes are known to produce powerful degradation enzymes (Bonito *et al*., 2010). In our study, Phanerochaete chrysosporium, which produced cellobiose dehydrogenase, was detected in earlier phase (Table 4).

**Table 3:** Most abundant fungal genera for the predominant phyla

Taxonomy	Protein number
Ascomycota	
Saccharomycetes	114
Saccharomyces	51
Candida	14
Eremothecium	14
Kluyveromyces	11
Yarrowia	6
Pichia	4
Schizosaccharomycetes	25
Schizosaccharomyces	25
Sordariomycetes	16
Neurospora	8
Chaetomium	3
Eurotiomycetes	16
Aspergillus	13
Basidiomycota	9
Microsporidia	8

**Table 4:** Proteins belonged to carbohydrate metabolic pathway identified in the composting metaproteome

Protein name	Accession	Functional group	Species	Taxonomy
LS1				
Glyceraldehyde-3-phosphate dehydrogenase	P0A1P0	Glycolysis / Gluconeogenesis	*Salmonella enterica*	*Gammaproteobacteria*
Enolase	A8AY46	Glycolysis / Gluconeogenesis	*Streptococcus gordonii*	Bacilli; Lactobacillales
Glyceraldehyde-3-phosphate dehydrogenase 2	Q6FSM4	Glycolysis / Gluconeogenesis	*Candida glabrata*	Saccharomycetes
Glyceraldehyde-3-phosphate dehydrogenase	Q00584	Glycolysis / Gluconeogenesis	*Claviceps purpurea*	Sordariomycetes
Acetyl-coenzyme A synthetase	Q3IFM6	Pyruvate metabolism	*Pseudoalteromonas haloplanktis*	*Gammaproteobacteria*
Acetate kinase	A4VSL6	Pyruvate metabolism	*Streptococcus suis*	Bacilli; Lactobacillales
Aconitate hydratase 2	Q9I2V5	TCA	*Pseudomonas aeruginosa*	*Gammaproteobacteria*
Aconitate hydratase 1	Q9I3F5	TCA	*Pseudomonas aeruginosa*	*Gammaproteobacteria*
Isocitrate lyase	Q9I0K4	TCA	*Pseudomonas aeruginosa*	*Gammaproteobacteria*
Malate synthase G	Q3K5N4	TCA	*Pseudomonas fluorescens*	*Gammaproteobacteria*
Malate dehydrogenase	Q4FQU7	TCA	*Psychrobacter arcticus*	*Gammaproteobacteria*
Isocitrate dehydrogenase [NADP] 2	P41561	TCA	*Colwellia maris*	*Gammaproteobacteria*
Malate dehydrogenase	A5UCQ1	TCA	*Haemophilus influenzae*	*Gammaproteobacteria*
Citrate synthase	O33915	TCA	*Sinorhizobium meliloti*	*Alphaproteobacteria*
Malate synthase G	Q2J0A5	TCA	*Rhodopseudomonas palustris*	*Alphaproteobacteria*
Malate synthase G	C0RES0	TCA	*Brucella melitensis*	*Alphaproteobacteria*
Malate dehydrogenase	Q2GCH6	TCA	*Neorickettsia sennetsu*	*Alphaproteobacteria*
Malate synthase G	A1UGU7	TCA	*Mycobacterium* sp.	Actinobacteria
Succinate dehydrogenase [ubiquinone] flavoprotein subunit, mitochondrial	Q00711	TCA	*Saccharomyces cerevisiae*	Saccharomycetes
Citrate synthase, mitochondrial	P00890	TCA	*Saccharomyces cerevisiae*	Saccharomycetes
Aconitate hydratase, mitochondrial	P19414	TCA	*Saccharomyces cerevisiae*	Saccharomycetes
Malate dehydrogenase, cytoplasmic	P83778	TCA	*Candida albicans*	Saccharomycetes
Putative exoglucanase type C	P46238	Cellulose degradation	*Fusarium oxysporum*	Sordariomycetes
Exoglucanase 1	P38676	Cellulose degradation	*Neurospora crassa*	Sordariomycetes
Cellobiose dehydrogenase	Q01738	Cellulose degradation	*Phanerochaete chrysosporium*	Basidiomycota
Mannose-6-phosphate isomerase	Q870Y1	Amino sugar and nucleotide sugar metabolism	*Neurospora crassa*	Sordariomycetes
Endo-1,3(4)-beta-glucanase 1	P53753	Cell wall degradation	*Saccharomyces cerevisiae*	Saccharomycetes
LS2				
Glyceraldehyde-3-phosphate dehydrogenase	P0A1P0	Glycolysis / Gluconeogenesis	*Salmonella enterica*	*Gammaproteobacteria*
Glyceraldehyde-3-phosphate dehydrogenase	Q00584	Glycolysis / Gluconeogenesis	Claviceps purpurea	Sordariomycetes
Phosphoenolpyruvate carboxykinase [GTP]	A8L175	Glycolysis / Gluconeogenesis	*Frankia* sp.	Actinobacteria
Fructose-1,6-bisphosphatase class 1	C0QTP7	Glycolysis / Gluconeogenesis	*Persephonella marina*	Aquificae
Glyoxylate/hydroxypyruvate reductase B	Q0W9V5	Pyruvate metabolism	*Yersinia pestis*	*Gammaproteobacteria*
Acetyl-coenzyme A synthetase	Q9F7R5	Pyruvate metabolism	*uncultured marine gamma proteobacterium*	*Gammaproteobacteria*
Acetyl-coenzyme A synthetase 1	Q9Z3R3	Pyruvate metabolism	*Sinorhizobium meliloti*	*Alphaproteobacteria*
Pyruvate, phosphate dikinase	Q59754	Pyruvate metabolism	*Sinorhizobium meliloti*	*Alphaproteobacteria*
Malate dehydrogenase	A5WGM2	TCA	*Psychrobacter sp.*	*Gammaproteobacteria*
Citrate lyase subunit beta	P17725	TCA	*Klebsiella pneumoniae*	*Gammaproteobacteria*
Probable malate : quinone oxidoreductase	B3QK65	TCA	*Rhodopseudomonas palustris*	*Alphaproteobacteria*
Succinate dehydrogenase flavoprotein subunit	Q59661	TCA	*Paracoccus denitrificans*	*Alphaproteobacteria*
Succinate dehydrogenase iron-sulfur subunit	Q1RGP3	TCA	*Rickettsia bellii*	*Alphaproteobacteria*
Succinate dehydrogenase flavoprotein subunit	P08065	TCA	*Bacillus subtilis*	Bacilli; Bacillales
Malate dehydrogenase	C1DB66	TCA	*Laribacter hongkongensis*	*Betaproteobacteria*
Endoglucanase	P10475	Cellulose degradation	*Bacillus subtilis*	Bacilli; Bacillales
Endoglucanase E-2	P26222	Cellulose degradation	*Thermobifida fusca*	Actinobacteria
Endo-1,4-beta-xylanase B	P26515	Hemicellulose degradation	*Streptomyces lividans*	Actinobacteria
1,4-alpha-glucan branching enzyme GlgB	B0TZI5	Starch and sucrose metabolism	*Francisella philomiragia*	*Gammaproteobacteria*
Phosphoglucosamine mutase	Q5NNT4	Amino sugar and nucleotide sugar metabolism	*Zymomonas mobilis*	*Alphaproteobacteria*
Phosphoglucosamine mutase	A8AWM5	Amino sugar and nucleotide sugar metabolism	*Streptococcus gordonii*	Bacilli; Lactobacillales
Beta-N-acetylhexosaminidase	P49610	Amino sugar and nucleotide sugar metabolism	*Streptococcus pneumoniae*	Bacilli; Lactobacillales
2-keto-3-deoxy-L-rhamnonate aldolase	Q0TFJ7	Fructose and mannose metabolism	*Escherichia coli*	*Gammaproteobacteria*
Xylose isomerase	P19148	Fructose and mannose metabolism	*Thermoanaerobacterium thermosulfurigenes*	Clostridia
LS3				
6-phosphofructokinase	A8GLB0	Glycolysis / Gluconeogenesis	*Serratia proteamaculans*	*Gammaproteobacteria*
Glyceraldehyde-3-phosphate dehydrogenase	P24750	Glycolysis / Gluconeogenesis	*Escherichia hermannii*	*Gammaproteobacteria*
Glyceraldehyde-3-phosphate dehydrogenase	O32755	Glycolysis / Gluconeogenesis	*Lactobacillus delbrueckii*	Bacilli; Lactobacillales
Hexokinase-2	P50521	Glycolysis / Gluconeogenesis	*Schizosaccharomyces pombe*	Schizosaccharomycetes
Triosephosphate isomerase	Q9HGY8	Glycolysis / Gluconeogenesis	*Aspergillus oryzae*	Eurotiomycetes
Glyceraldehyde-3-phosphate dehydrogenase	Q00584	Glycolysis / Gluconeogenesis	*Claviceps purpurea*	Sordariomycetes
Phosphoenolpyruvate carboxykinase [GTP]	A8L175	Glycolysis / Gluconeogenesis	*Frankia* sp.	Actinobacteria
Aryl-phospho-beta-D-glucosidase BglC	P42403	Glycolysis / Gluconeogenesis	*Bacillus subtilis*	Bacilli; Bacillales
Pyruvate dehydrogenase E1 component	Q59637	Pyruvate metabolism	*Pseudomonas aeruginosa*	*Gammaproteobacteria*
Glyoxylate/hydroxypyruvate reductase B	Q0W9V5	Pyruvate metabolism	*Yersinia pestis*	*Gammaproteobacteria*
Acetyl-coenzyme A synthetase	Q9F7R5	Pyruvate metabolism	uncultured marine *Gammaproteobacterium*	*Gammaproteobacteria*
Acetyl-coenzyme A synthetase 1	Q9Z3R3	Pyruvate metabolism	*Sinorhizobium meliloti*	*Alphaproteobacteria*
Malate dehydrogenase	A5WGM2	TCA	*Psychrobacter* sp.	*Gammaproteobacteria*
Citrate lyase subunit beta	P17725	TCA	*Klebsiella pneumoniae*	*Gammaproteobacteria*
Succinate dehydrogenase flavoprotein subunit	Q59661	TCA	*Paracoccus denitrificans*	*Alphaproteobacteria*
Malate dehydrogenase	B2JQD2	TCA	*Burkholderia phymatum*	*Betaproteobacteria*
Succinate dehydrogenase flavoprotein subunit	P08065	TCA	*Bacillus subtilis*	Bacilli; Bacillales
Malate synthase G	A4IN50	TCA	*Geobacillus thermodenitrificans*	Bacilli; Bacillales
Endoglucanase	P10475	Cellulose degradation	*Bacillus subtilis*	Bacilli; Bacillales
Endoglucanase E-2	P26222	Cellulose degradation	*Thermobifida fusca*	Actinobacteria
Probable beta-glucosidase I	A2R989	Cellulose degradation	*Aspergillus niger*	Eurotiomycetes
Endo-1,4-beta-xylanase B	P26515	Hemicellulose degradation	*Streptomyces lividans*	Actinobacteria
1,4-alpha-glucan branching enzyme GlgB	A8GKV0	Starch and sucrose metabolism	*Serratia proteamaculans*	*Gammaproteobacteria*
Phosphoglucosamine mutase	Q5NNT4	Amino sugar and nucleotide sugar metabolism	*Zymomonas mobilis*	*Alphaproteobacteria*
2-keto-3-deoxy-L-rhamnonate aldolase	Q31Z78	Fructose and mannose metabolism	*Shigella boydii*	*Gammaproteobacteria*
Sensor protein ChvG	P72292	Cell wall degradation	*Sinorhizobium meliloti*	*Alphaproteobacteria*

Compared with bacteria, the diversity of fungi was lower, but also generally the species richness of fungi is not as high as that of bacteria. This finding was congruent with those of other investigators (Hultman *et al*., 2010; De Gannes *et al*., 2013a).

### Microbial community differs between sampling times

**Figure 1:**
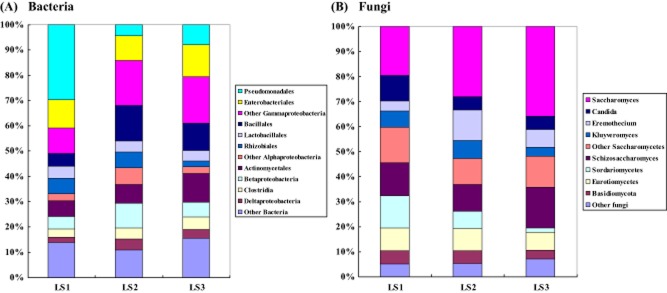
Phylogenetic assignment of (A) bacterial and (B) fungal proteins detected in the three samples. Relative abundances were calculated from the sum of SpCns found for each group at the respective sampling sites. (A) The sum is 717 565 and 443 respectively; (B) the sum is 315 225 and 242 respectively.

The protein assignments illustrated that specific taxa of bacteria and fungi had different temporal abundances (Fig. 1). The Bacilli, *Gammaproteobacteria* and *Alphaproteobacteria* were further divided into the orders in order to study the phylogenetic differences between the three samples (Fig. 1A). The largest increase in the proportion of plant spectra was observed in Bacillales (4.8 to 14.1%) in the active phase, followed by Actinomycetales (6.2 to 11.3%). The proportion of Pseudomonadales sharply decreased in the active phase from 29.7% to 4.3%. As the composts transitioned into the curing phase, the proportion of order Rhizobiales decreased from 6.2% to 2.1%.

Bacteria may be present throughout the composting process as active or dormant cells, or as spores. Only their numbers and level of activity change during the composting process (Gentleman *et al*., 2004). Microorganisms belonging to the Bacillales and Actinobacteria, known to be critical for an efficient composting process, were sufficiently abundant in the waste to inoculate the subsequent composting process. However, the numbers were found to be lower than those of the Lactobacillales in most large-scale reactors, because the oxygen supply may be restricted (Partanen *et al*., 2010; Watanabe *et al*., 2010). To improve this situation, the active phase of the composting process should be steered towards conditions that favour thermotolerant microbes such as Bacillales and Actinobacteria. In this study, the main adjustment was turning the compost pile mechanically according to a schedule. The Bacillales and Actinobacteria groups became dominant as composting progressed, and there was no increase in Lactobacillales, suggesting that improving the internal aeration of the composting mass by mechanized turning was an efficient way to improve the performance of the composting plant.

The sharp decrease of the order Pseudomonadales could be explained by the effects of high temperature on Gram-negative bacteria (Dees and Ghiorse, 2001). On the other hand, as previously reported, the type of indigenous microorganisms such as actinomycetes and fungi is critical for the suppression of pathogens growth in compost (Kim *et al*., 2011). This might be another reason why the order Pseudomonadales decreased.

Since proteins representing the Saccharomycetes were by far the largest group, this class was further divided into genuses in order to study the phylogenetic differences (Fig. 1B). The largest increase in the proportion was observed in Saccharomyces (19.5% to 35.7%), followed by Schizosaccharomyces (13.0% to 16.1%). The smallest decrease was observed in Kluyveromyces (6.5% to 3.6%), followed by Candida (10.4% to 5.4%) and Sordariomycetes (13.0% to 1.8%). The proportion of Eremothecium rose to 12.3% in the active phase and decreased to 7.1% in the curing phase.

A high proportion of ascomycetous yeasts, included order Saccharomycetes and Schizosaccharomycetes, were detected at all examined phases. Yeasts being able to grow at low pH may promote a reduction in the acidity and an increase in the growth of thermophilic bacteria (Choi and Park, 1998). Previous studies have reported on the presence of yeasts during the early phases of composting (Bonito *et al*., 2010; Hultman *et al*., 2010). Although plant and human pathogens were found at the start of active phase, few pathogenic species were recovered from samples representing the curing phase of composting suggesting that the composting process is effective in the removal of fungal pathogens.

### Carbohydrate metabolism in composting process

The most prevalent bacterial and fungal proteins in the metaproteome were components of carbohydrate metabolic enzymes (77 proteins; 12.0% of the total metaproteome), translation proteins (74; 11.6%), ribosomal subunits (60; 9.4%) and transport proteins (42; 6.6%). Deoxyribonucleic acid replication and repair proteins (41), amino acid metabolic enzymes (36), protein chaperones (36), transcription proteins (36) and outer membrane proteins (32) were also detected in the metaproteome (Supplementary Table S1 and S2).

**Figure 2:**
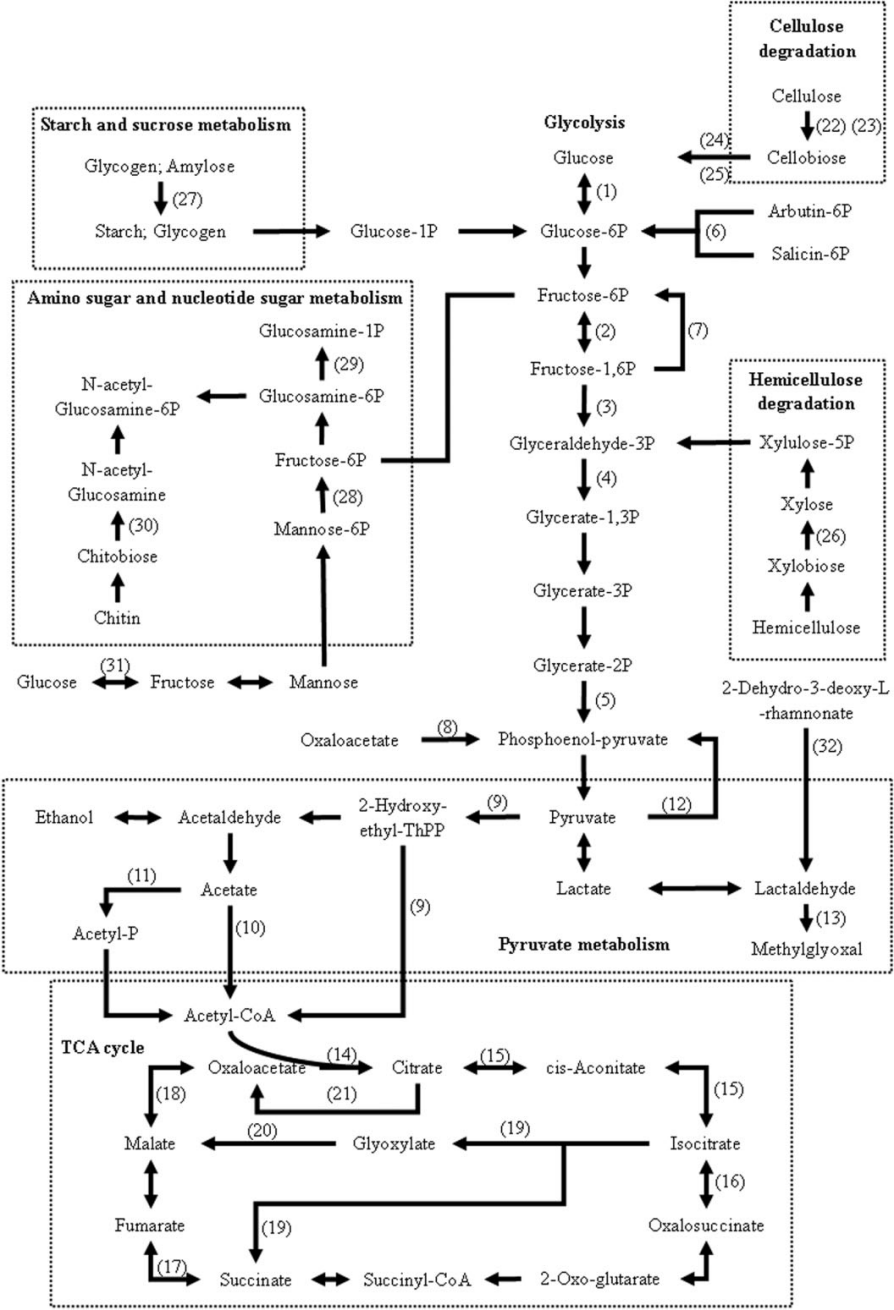
Depiction of the carbohydrate metabolic characteristics of microbial communities inferred from the metaproteome. Proteins shown are: (1) Hexokinase; (2) 6-phosphofructokinase; (3) Triosephosphate isomerase; (4) Glyceraldehyde-3-phosphate dehydrogenase; (5) Enolase; (6) Aryl-phospho-beta-D-glucosidase; (7) Fructose-1,6-bisphosphatase; (8) Phosphoenolpyruvate carboxykinase; (9) Pyruvate dehydrogenase; (10) Acetyl-coenzyme A synthetase; (11) Acetate kinase; (12) Pyruvate, phosphate dikinase; (13) Glyoxylate/hydroxypyruvate reductase; (14) Citrate synthase; (15) Aconitate hydratase; (16) Isocitrate dehydrogenase; (17) Succinate dehydrogenase; (18) Malate dehydrogenase; (19) Isocitrate lyase; (20) Malate synthase; (21) Citrate lyase; (22) Exoglucanase; (23) Endoglucanase; (24) Cellobiose dehydrogenase; (25) Beta-glucosidase; (26) Endo-1,4-beta-xylanase B; (27)1,4-alpha-glucan branching enzyme GlgB; (28) Mannose-6-phosphate isomerase; (29) Phosphoglucosamine mutase; (30) Beta-N-acetylhexosaminidase; (31) 2-keto-3-deoxy-L-rhamnonate aldolase; (32) Xylose isomerase.

Composting can be defined as an aerobic process of decomposition of organic matter. The active phase of composting involves the degradation of easily degradable compounds such as carbohydrates, amino acids, proteins and lipids. In the current metaproteome, the carbohydrate metabolic enzymes comprised a higher proportion, suggesting that the carbohydrate metabolism was the principal metabolic pathway in composting process. These enzymes were involved in different carbohydrate metabolic pathways (Fig. 2). The largest amounts (29 of the 77 total) of carbohydrate metabolic enzymes were involved in the citrate cycle (TCA cycle, Krebs cycle), including malate dehydrogenase (9), succinate dehydrogenase (6) and malate synthase (5), which were much more abundant than the enzymes were essential for glycolysis/gluconeogenesis (16), pyruvate metabolism (10), cellulose degradation (8) and amino sugar and nucleotide sugar metabolism (5) (Table 4).

The identified enzymes related to carbohydrate metabolism were mainly affiliated to *Gammaproteobacteria* (25), *Alphaproteobacteria* (16), Bacilli (11), Actinobacteria (7), Saccharomycetes (6) and Sordariomycetes (6) members (Table 4). The proportion of Saccharomyces proteins increased significantly in composting process, but no proteins involved in carbohydrate metabolism were detected except the earlier phase.

### Key cellulolytic community in different composting stages is different

Ten cellulase and hemicellulase were identified in composting samples (Table 4). All three cellulase in the sample LS1 matched to fungi, included *Fusarium oxysporum*, *Neurospora crassa* and *Phanerochaete chrysosporium*, whereas all cellulase and hemicellulase in the sample LS2 matched to bacteria, such as Bacillus subtilis and Thermobifida fusca.

The most common complex carbohydrate available in the composting substrates is cellulose. A key function of bacteria and fungi during composting is to produce cellulolytic enzymes (Hubbe *et al*., 2010). In the earlier phase, cellulolytic fungal communities specifically targeted the breakdown of the cellulose. However, the cellulolytic fungal communities were replaced by a purely bacterial one in the active phase. As the composts transitioned into the curing phase, cellulolytic fungal communities were recovered and the mixed community of bacteria and fungi enhance the decomposition of cellulose. Previously, it has been shown that thermophilic fungi are active through the thermophilic phase (Tchobanoglous *et al*., 1993). Our results revealed that the cellulolytic fungal communities played a vital role in earlier phase, after which they gradually disappeared and cellulolytic bacterial communities gradually played a leading role. This suggested that the key microbiologic population for cellulose degradation in different composting stages was different.

Cellulose is a polymer of glucose, which is digested by a variety of enzymes. Cellulose itself requires three types of enzymes for its decomposition: Endoglucanases hydrolyse accessible intramolecular β-1,4-glucosidic bonds of cellulose chains randomly to produce new chain ends; exoglucanases or cellobiohydrolases processively cleave cellulose chains at the ends to release soluble cellobiose or glucose; and β-glucosidases hydrolyse cellobiose to glucose in order to eliminate cellobiose inhibition (Percival Zhang *et al*., 2006). In the current study, all three types of cellulases were detected in the metaproteome, suggesting the effective breakdown of cellulose in the composting process. Interestingly, exoglucanases and β-glucosidases were only produced by fungi, whereas endoglucanases were only produced by bacteria. Beta-glucosidase is a limited enzyme in cellulose decomposition, and it was only found in the curing phase. This indicated that the curing phase may be the key phase, in which mixed communities of bacteria and fungi worked together to digested cellulose.

### Nitrogen flows in composting process

In composting processes, the nitrogen flows can be best described through ammonia (NH_3_) dynamics, which encompasses the production, volatilization, conversion and assimilation of ammonia (Szántó, 2009). Proteins that could be assigned to a function in the production of ammonia were identified in the metaproteome, including proteases and urease (Table 5). This suggested that the protein and urea were the source of ammonia in the municipal solid waste.

**Table 5:** Proteins belonged to nitrogen metabolism identified in the composting metaproteome

Protein name	Accession	Functional group	Species	Taxonomy
Lon protease 2	P36774	Protein degradation	*Myxococcus xanthus*	*Deltaproteobacteria*
Lon protease homolog 2, peroxisomal	Q6CWS4	Protein degradation	*Kluyveromyces lactis*	Saccharomycetes
Probable M18 family aminopeptidase 2	A4VJG1	Protein degradation	*Pseudomonas stutzeri*	*Gammaproteobacteria*
Probable cytosol aminopeptidase	Q0SHL0	Protein degradation	*Rhodococcus jostii*	Actinobacteria
ATP-dependent zinc metalloprotease FtsH	Q03Z46	Protein degradation	*Leuconostoc mesenteroides*	Bacilli; Lactobacillales
ATP-dependent Clp protease ATP-binding subunit ClpX	B2JGL6	Protein degradation	*Burkholderia phymatum*	*Betaproteobacteria*
ATP-dependent protease ATPase subunit HslU	Q73NE3	Protein degradation	*Treponema denticola*	Spirochaetes
Urease subunit alpha	B1JR71	Urea degradation	*Yersinia pseudotuberculosis*	*Gammaproteobacteria*
[Protein-PII] uridylyltransferase	Q3J5H6	Nitrogen fixation	*Rhodobacter sphaeroides*	*Alphaproteobacteria*
Nitrous-oxide reductase	P19573	Denitrification	*Pseudomonas stutzeri*	*Gammaproteobacteria*

Nitrogen can be lost as NO_3_^-^, NO_2_^-^, N_2_ or N_2_O through the process nitrification–denitrification (Szántó, 2009). Only one denitrification enzyme (nitrous-oxide reductase) produced by *Pseudomonas stutzeri* was detected in the metaproteome (Table 5). This indicated that denitrification enzymes production of other denitrifying bacteria was not obvious, and the Pseudomonas might be the key player in denitrification pathway in composting process.

The high abundance of translation proteins (11.6%) and amino acid metabolic enzymes (5.6%) could be attributed to nitrogen assimilation in composting process. Microbial biomass such as bacteria, viruses and fungi require nitrogen for their cell matter, which they gain from ammonium through assimilation into the cell tissue (Szántó, 2009). Assimilation is the immobilization of the ammoniacal nitrogen by the consecutive amino acid and protein synthesis. Another nitrogen assimilation route is the process of nitrogen fixation, in which N_2_ gas is converted into the biologically useful forms. In the current study, nitrogen fixation enzyme was only produced by Rhodobacter sphaeroides (Table 5).

## Conclusions

Our metaproteomic analysis provided insight into microbial succession and in the activity of certain phylogenetic groups in a large-scale composting plant. The investigation of microbial succession revealed that Bacillales, Actinobacteria and Saccharomyces increased significantly with respect to abundance in composting process. The *Gammaproteobacteria* were the single largest group accounting for 40.3% of the total bacterial proteins.

The carbohydrate metabolism was the principal metabolic pathway in composting process. Cellulose and hemicellulose were the main carbon sources. Three types of cellulase essential for cellulose degradation were detected. Fungi were found to be the main producers of cellulase in earlier phase. Only bacterial, but no fungal, cellulolytic enzymes were detected at the end of the active phase, a finding that strongly supported our conclusion that the key microbiologic population for cellulose degradation in different composting stages was different, which did not support the concept that the thermophilic fungi are active through the thermophilic phase. In the curing phase, mixed community of bacteria and fungi enhance decomposition of cellulose, suggesting that the curing phase may be a rate-limiting phase in the cellulose decomposition process.

Though some denitrifying bacteria such *Azotobacter vinelandii*, *Azotobacter chroococcum* and *Paracoccus denitrificans* were detected in the samples, only *Pseudomonas stutzeri* produced denitrification enzyme. This indicated that denitrification enzymes production of other denitrifying bacteria was not obvious, and the Pseudomonas might be the key player in denitrification pathway in composting process.

We are fully aware that the metaproteomics-based approach suffers from certain limitations such as the protein extraction efficiency and data analysis. For complex environmental samples, it has been estimated that « 1% of the total metaproteome can be resolved using current method (Wilmes and Bond, 2006; Leary *et al*., 2013). Database selection has a significant impact in metaproteomics, and provides critical indications for improving depth and reliability of metaproteomic results (Tanca *et al*., 2013). Nevertheless, we believe that the metaproteome analysis provides a deep insight into molecular details of the composting process. Thus, further proteome analyses will help to elucidate the main metabolic pathways occurring at the microbial level in different composting systems.

## Experimental procedures

### Sampling

Samples were collected from Asuwei Composting Plant in Beijing, China. More details of this composting plant and how composting was carried out has been described elsewhere (He *et al*., 2011). The entire composting process was divided into the active and curing phases. The active phase was carried out for 20 days during which the pile was turned every 2 days by forklift, and the average temperature was maintained at around 60°C. The curing phase took 30 days to complete, and the pile was turned mechanically every 7 days. The temperature began to decrease in 20 days and reached a constant level with ambient temperature after 50 days. Triplicate samples were collected at different points from the top to the bottom of the composting plies after 5, 20 and 50 days. At different sampling-times, triplicate samples were mixed, and the composite sample was divided into two parts. Metaproteome analyses were performed in duplicate.

### Physicochemical analyses

The pH was measured with a pH meter (SevenEasy S20, Mettler Toledo, Shanghai, China) and a standard electrode by mixing the samples with deionized water at a weight/volume ratio of 1:10 (Laos *et al*., 2002). Water content was determined gravimetrically by drying the sample at 105°C for 6 h. Total carbon was evaluated by dry combustion at 550°C (Navarro *et al*., 1990), and total nitrogen by the Kjeldahl digestion analysis. Information on the sampling and the physical-chemical properties at the sampling dates is illustrated in Table 1.

### Metaproteomics

Protein extraction from 5 g fresh composting samples followed the method of Keiblinger and colleagues (2012) using 20 ml extraction buffer [1: 1 (v: v) SDS–phenol buffer: 50 mM Tris, 1% SDS pH 7.5 + phenol (pH 8.0)].

Extracted proteins were separated by one-dimensional SDS-PAGE. Before electrophoresis, the protein pellet was dissolved in appropriate volumes sample buffer for SDS-PAGE and incubated 20 min at 100°C. After centrifugation, the supernatant (5 μl) was loaded on SDS gel (5% stacking gel, 10% separating gel) using a Mini-PROTEAN Tetra system (Bio-Rad, Beijing, China). After electrophoresis, gel was stained with Coomassie brilliant blue R250.

For identification of proteins from composting samples, each lane of the SDS-PAGE was cut into seven slices. The gel slices were further cut into 1 mm^3^ gel pieces and subjected to immediate in-gel tryptic digestion (Promega, Madison, WI, USA). Digested peptides were separated by nano-LC (Ultimate 3000, Dionex, Sunnyvale, CA, USA; column: Venusil XBP-C18, 3.0 μm, 150 × 0.075 mm, Agela Technologies, Wilmington, DE, USA; eluent: 0.1% formic acid, 0% to 60% acetonitrile) and analysed by MS/MS (Q Exactive, Thermo Scientific, Pittsburgh PA, USA) as described earlier (Williams *et al*., 2012).

Database searches were carried out with MS/MS ion search (MASCOT, http://www.matrixscience.com) against a non-redundant protein database, SwissProt2013 xyzzy (539 616 sequences; 191 569 459 residues). The following search parameters were applied: (i) trypsin was chosen as protein-digesting enzyme and one missed cleavages were tolerated, (ii) carbamidomethyl-cysteine was chosen as fixed modification and (iii) Gln- > pyro-Glu (N-term Q) and oxidation (M) were chosen as variable modification. Searches were performed with a peptide mass tolerance of ± 15 ppm and a fragment mass tolerance of ± 20 mmu. Mascot searches with a false discovery rate > 5% were rejected. Protein matches were only accepted if they were identified by a minimum of one unique peptide. All proteins were manually annotated with the aid of blastp, and the protein hit that showed the highest sequence identity was recorded, including the organism name (Supplementary Table S1 and S2). Higher protein abundance is represented by a higher number of MS/MS spectra acquired from peptides of the respective protein. Thus, protein abundances were calculated based on the normalized spectral counts (SpCn; Piersma *et al*., 2010). An important consideration with spectrum counting and similar approaches is the fact that small proteins tend to have fewer peptides identified per protein compared with large proteins (Florens *et al*., 2006). All phylogenetic group abundances presented in the metaproteome are based on SpCns.

## Conflict of interest

None declared.

## Supporting Information

**Table S1.** Detailed identifications of bacterial proteins extracted from samples. **Table S2.** Detailed identifications of fungal proteins extracted from samples.
