# Supplementary material for: Metaproteomics reveals major microbial players and their biodegradation functions in a large-scale aerobic composting plant
Source: Microb Biotechnol. 2015 May 19;8(6):950–60. doi: 10.1111/1751-7915.12290 (PMC4621448; doi:10.1111/1751-7915.12290)
Supplement: Supplementary file 1 — Table S1. Detailed identifications of bacterial proteins extracted from samples. Table S2. Detailed identifications of fungal proteins extracted from samples. [file mbt20008-0950-sd1.zip › MBT2_12290_supp-0001-SupplementaryTableS1.doc]

**Supplementary Table S1 Detailed identifications of bacterial proteins extracted from samples**

| **Protein** | **Accession** | **Score** | **Assigned specrra** | **Unique peptides** | **Sequence coverage** | **Function** | **Source** | **Organism** |
| --- | --- | --- | --- | --- | --- | --- | --- | --- |
| **Samle LS 1** | | | | | | | | |
| Putative flagellin YvzB | O34366 | 223 | 14 | 2 | 18.1 | Cell motility | Bacillus subtilis subsp. subtilis str. 168 | Bacteria; Firmicutes; Bacilli; Bacillales; Bacillaceae; Bacillus. |
| Protein MraZ | Q9K9R9 | 93 | 5 | 1 | 5.6 | Cell division | Bacillus halodurans C-125 | Bacteria; Firmicutes; Bacilli; Bacillales; Bacillaceae; Bacillus. |
| Ribonucleoside-diphosphate reductase NrdEB subunit alpha | O31875 | 41 | 4 | 2 | 1.8 | Replication and repair | Bacillus subtilis subsp. subtilis str. 168 | Bacteria; Firmicutes; Bacilli; Bacillales; Bacillaceae; Bacillus. |
| Probable tRNA sulfurtransferase | Q9K804 | 37 | 2 | 1 | 1.7 | Cofactor biosynthesis | Bacillus halodurans C-125 | Bacteria; Firmicutes; Bacilli; Bacillales; Bacillaceae; Bacillus. |
| Probable tautomerase YusQ | O32183 | 32 | 1 | 1 | 6.3 | Other | Bacillus subtilis subsp. subtilis str. 168 | Bacteria; Firmicutes; Bacilli; Bacillales; Bacillaceae; Bacillus. |
| tRNA uridine 5-carboxymethylaminomethyl modification enzyme MnmG | A0RLR1 | 20 | 1 | 1 | 1 | Translation | Bacillus thuringiensis str. Al Hakam | Bacteria; Firmicutes; Bacilli; Bacillales; Bacillaceae; Bacillus; Bacillus cereus group. |
| Catalase | Q4L643 | 165 | 11 | 5 | 7 | Oxidative stress | Staphylococcus haemolyticus JCSC1435 | Bacteria; Firmicutes; Bacilli; Bacillales; Staphylococcus. |
| DNA topoisomerase 4 subunit B | Q59961 | 54 | 1 | 1 | 1.1 | Replication and repair | Streptococcus pneumoniae TIGR4 | Bacteria; Firmicutes; Bacilli; Lactobacillales; Streptococcaceae; Streptococcus. |
| Enolase | A8AY46 | 48 | 2 | 2 | 8.3 | Carbohydrate metabolism; Glycolysis | Streptococcus gordonii str. Challis | Bacteria; Firmicutes; Bacilli; Lactobacillales; Streptococcaceae; Streptococcus. |
| Acetate kinase | A4VSL6 | 35 | 2 | 1 | 2 | Carbohydrate metabolism; Pyruvate metabolism | Streptococcus suis 05ZYH33 | Bacteria; Firmicutes; Bacilli; Lactobacillales; Streptococcaceae; Streptococcus. |
| Putrescine carbamoyltransferase | B7S4N1 | 33 | 1 | 1 | 3.2 | Amino acid metabolism | Streptococcus ratti | Bacteria; Firmicutes; Bacilli; Lactobacillales; Streptococcaceae; Streptococcus. |
| Probable potassium transport system protein kup 1 | Q9CHU5 | 23 | 1 | 1 | 1.6 | Transport; Ion transport | Lactococcus lactis subsp. lactis Il1403 | Bacteria; Firmicutes; Bacilli; Lactobacillales; Streptococcaceae; Lactococcus. |
| Isoleucine--tRNA ligase | Q1GAS8 | 63 | 7 | 1 | 0.8 | Translation | Lactobacillus delbrueckii subsp. bulgaricus ATCC 11842 | Bacteria; Firmicutes; Bacilli; Lactobacillales; Lactobacillaceae; Lactobacillus. |
| Peptide chain release factor 2 | Q88YL5 | 18 | 4 | 3 | 5.6 | Translation | Lactobacillus plantarum WCFS1 | Bacteria; Firmicutes; Bacilli; Lactobacillales; Lactobacillaceae; Lactobacillus. |
| Chromosome partition protein Smc | B9E1H0 | 35 | 3 | 3 | 1.8 | Replication and repair | Clostridium kluyveri NBRC 12016 | Bacteria; Firmicutes; Clostridia; Clostridiales; Clostridiaceae; Clostridium. |
| Exodeoxyribonuclease 7 large subunit | B8I3A9 | 33 | 1 | 1 | 2 | Replication and repair | Clostridium cellulolyticum H10 | Bacteria; Firmicutes; Clostridia; Clostridiales; Clostridiaceae; Clostridium. |
| Valine--tRNA ligase | Q891R5 | 19 | 1 | 1 | 1 | Translation | Clostridium tetani E88 | Bacteria; Firmicutes; Clostridia; Clostridiales; Clostridiaceae; Clostridium. |
| UDP-N-acetylglucosamine--N-acetylmuramyl-(pentapeptide) pyrophosphoryl-undecaprenol N-acetylglucosamine transferase | A6TS61 | 32 | 1 | 1 | 1.6 | Out membrane proteins; Cell wall biogenesis | Alkaliphilus metalliredigens QYMF | Bacteria; Firmicutes; Clostridia; Clostridiales; Clostridiaceae; Alkaliphilus. |
| 50S ribosomal protein L9 | Q2RM68 | 33 | 2 | 1 | 7.3 | Ribosomes | Moorella thermoacetica ATCC 39073 | Bacteria; Firmicutes; Clostridia; Thermoanaerobacterales; Thermoanaerobacteraceae; Moorella group; Moorella. |
| Chaperone protein DnaK | Q1Q7X0 | 5038 | 189 | 38 | 53.3 | Chaperone | Psychrobacter cryohalolentis K5 | Bacteria; Proteobacteria; Gammaproteobacteria; Pseudomonadales; Moraxellaceae; Psychrobacter. |
| Trigger factor | Q1Q8J3 | 425 | 11 | 5 | 15.9 | Chaperone | Psychrobacter cryohalolentis K5 | Bacteria; Proteobacteria; Gammaproteobacteria; Pseudomonadales; Moraxellaceae; Psychrobacter. |
| Malate dehydrogenase | Q4FQU7 | 241 | 8 | 4 | 16.1 | Carbohydrate metabolism; TCA | Psychrobacter arcticus 273-4 | Bacteria; Proteobacteria; Gammaproteobacteria; Pseudomonadales; Moraxellaceae; Psychrobacter. |
| 60 kDa chaperonin | Q1QDC9 | 236 | 15 | 12 | 31.7 | Chaperone | Psychrobacter cryohalolentis K5 | Bacteria; Proteobacteria; Gammaproteobacteria; Pseudomonadales; Moraxellaceae; Psychrobacter. |
| ATP synthase subunit beta | Q1Q899 | 96 | 2 | 2 | 5.7 | Energy metabolism | Psychrobacter cryohalolentis K5 | Bacteria; Proteobacteria; Gammaproteobacteria; Pseudomonadales; Moraxellaceae; Psychrobacter. |
| Elongation factor Tu | Q1Q8P2 | 78 | 3 | 3 | 7.1 | Translation | Psychrobacter cryohalolentis K5 | Bacteria; Proteobacteria; Gammaproteobacteria; Pseudomonadales; Moraxellaceae; Psychrobacter. |
| Nucleoside diphosphate kinase | Q1QD23 | 76 | 2 | 2 | 30.8 | Nucleotide metabolism | Psychrobacter cryohalolentis K5 | Bacteria; Proteobacteria; Gammaproteobacteria; Pseudomonadales; Moraxellaceae; Psychrobacter. |
| 50S ribosomal protein L29 | Q1QDH8 | 64 | 3 | 3 | 55.4 | Ribosomes | Psychrobacter cryohalolentis K5 | Bacteria; Proteobacteria; Gammaproteobacteria; Pseudomonadales; Moraxellaceae; Psychrobacter. |
| 50S ribosomal protein L1 | Q1Q8P6 | 57 | 4 | 3 | 13.7 | Ribosomes | Psychrobacter cryohalolentis K5 | Bacteria; Proteobacteria; Gammaproteobacteria; Pseudomonadales; Moraxellaceae; Psychrobacter. |
| Fatty acid oxidation complex subunit alpha | Q1Q8J9 | 52 | 3 | 2 | 3.1 | Lipid metabolism | Psychrobacter cryohalolentis K5 | Bacteria; Proteobacteria; Gammaproteobacteria; Pseudomonadales; Moraxellaceae; Psychrobacter. |
| LPS-assembly protein LptD | Q1QA28 | 49 | 7 | 5 | 4.5 | Out membrane proteins | Psychrobacter cryohalolentis K5 | Bacteria; Proteobacteria; Gammaproteobacteria; Pseudomonadales; Moraxellaceae; Psychrobacter. |
| 50S ribosomal protein L11 | Q1Q8P5 | 48 | 1 | 1 | 6.3 | Ribosomes | Psychrobacter cryohalolentis K5 | Bacteria; Proteobacteria; Gammaproteobacteria; Pseudomonadales; Moraxellaceae; Psychrobacter. |
| Chaperone protein HtpG | Q1Q978 | 38 | 2 | 2 | 3.3 | Chaperone | Psychrobacter cryohalolentis K5 | Bacteria; Proteobacteria; Gammaproteobacteria; Pseudomonadales; Moraxellaceae; Psychrobacter. |
| 50S ribosomal protein L5 | Q1QDH4 | 32 | 2 | 2 | 15.2 | Ribosomes | Psychrobacter cryohalolentis K5 | Bacteria; Proteobacteria; Gammaproteobacteria; Pseudomonadales; Moraxellaceae; Psychrobacter. |
| 50S ribosomal protein L6 | Q4FUE1 | 29 | 1 | 1 | 6.2 | Ribosomes | Psychrobacter arcticus 273-4 | Bacteria; Proteobacteria; Gammaproteobacteria; Pseudomonadales; Moraxellaceae; Psychrobacter. |
| 30S ribosomal protein S17 | Q1QDH7 | 28 | 1 | 1 | 17.6 | Ribosomes | Psychrobacter cryohalolentis K5 | Bacteria; Proteobacteria; Gammaproteobacteria; Pseudomonadales; Moraxellaceae; Psychrobacter. |
| Aconitate hydratase 2 | Q9I2V5 | 282 | 12 | 4 | 5.2 | Carbohydrate metabolism; TCA | Pseudomonas aeruginosa PAO1 | Bacteria; Proteobacteria; Gammaproteobacteria; Pseudomonadales; Pseudomonadaceae; Pseudomonas. |
| Fimbrial assembly protein PilQ | P34750 | 256 | 9 | 7 | 12.3 | Cell motility | Pseudomonas aeruginosa PAO1 | Bacteria; Proteobacteria; Gammaproteobacteria; Pseudomonadales; Pseudomonadaceae; Pseudomonas. |
| Nitrous-oxide reductase | P19573 | 223 | 13 | 5 | 11.3 | Nitrogen metabolism； denitrification | Pseudomonas stutzeri | Bacteria; Proteobacteria; Gammaproteobacteria; Pseudomonadales; Pseudomonadaceae; Pseudomonas. |
| B-type flagellin | P72151 | 219 | 7 | 3 | 7.2 | Cell motility | Pseudomonas aeruginosa PAO1 | Bacteria; Proteobacteria; Gammaproteobacteria; Pseudomonadales; Pseudomonadaceae; Pseudomonas. |
| Malate synthase G | Q3K5N4 | 178 | 6 | 3 | 3.9 | Carbohydrate metabolism; TCA | Pseudomonas fluorescens Pf0-1 | Bacteria; Proteobacteria; Gammaproteobacteria; Pseudomonadales; Pseudomonadaceae; Pseudomonas. |
| Uncharacterized protein PA3922 | Q9HX91 | 142 | 10 | 5 | 8.8 | Other | Pseudomonas aeruginosa PAO1 | Bacteria; Proteobacteria; Gammaproteobacteria; Pseudomonadales; Pseudomonadaceae; Pseudomonas. |
| Aconitate hydratase 1 | Q9I3F5 | 137 | 5 | 3 | 4.1 | Carbohydrate metabolism; TCA | Pseudomonas aeruginosa PAO1 | Bacteria; Proteobacteria; Gammaproteobacteria; Pseudomonadales; Pseudomonadaceae; Pseudomonas. |
| Major outer membrane lipoprotein | P11221 | 105 | 2 | 1 | 12 | Out membrane proteins | Pseudomonas aeruginosa PAO1 | Bacteria; Proteobacteria; Gammaproteobacteria; Pseudomonadales; Pseudomonadaceae; Pseudomonas. |
| 3-ketoacyl-CoA thiolase | A4XSM9 | 85 | 6 | 3 | 8.4 | Lipid metabolism | Pseudomonas mendocina ymp | Bacteria; Proteobacteria; Gammaproteobacteria; Pseudomonadales; Pseudomonadaceae; Pseudomonas. |
| 30S ribosomal protein S7 | Q4ZMP0 | 79 | 5 | 3 | 25.6 | Ribosomes | Pseudomonas syringae pv. syringae B728a | Bacteria; Proteobacteria; Gammaproteobacteria; Pseudomonadales; Pseudomonadaceae; Pseudomonas; Pseudomonas syringae. |
| 50S ribosomal protein L1 | B0KK57 | 61 | 4 | 2 | 10.4 | Ribosomes | Pseudomonas putida GB-1 | Bacteria; Proteobacteria; Gammaproteobacteria; Pseudomonadales; Pseudomonadaceae; Pseudomonas. |
| 30S ribosomal protein S10 | B0KK66 | 61 | 2 | 2 | 21.4 | Ribosomes | Pseudomonas putida GB-1 | Bacteria; Proteobacteria; Gammaproteobacteria; Pseudomonadales; Pseudomonadaceae; Pseudomonas. |
| Uncharacterized ATP-binding protein in capB 3'region | P72190 | 50 | 1 | 1 | 4.5 | Other | Pseudomonas fragi | Bacteria; Proteobacteria; Gammaproteobacteria; Pseudomonadales; Pseudomonadaceae; Pseudomonas. |
| Major cold shock protein CspA | P95459 | 47 | 2 | 1 | 26.1 | Stress response; Transcription regulation | Pseudomonas aeruginosa PAO1 | Bacteria; Proteobacteria; Gammaproteobacteria; Pseudomonadales; Pseudomonadaceae; Pseudomonas. |
| 50S ribosomal protein L11 | C3K2Y7 | 44 | 1 | 1 | 14 | Ribosomes | Pseudomonas fluorescens SBW25 | Bacteria; Proteobacteria; Gammaproteobacteria; Pseudomonadales; Pseudomonadaceae; Pseudomonas. |
| Isocitrate lyase | Q9I0K4 | 43 | 1 | 1 | 1.9 | Carbohydrate metabolism; TCA | Pseudomonas aeruginosa PAO1 | Bacteria; Proteobacteria; Gammaproteobacteria; Pseudomonadales; Pseudomonadaceae; Pseudomonas. |
| Dihydrolipoyllysine-residue succinyltransferase component of 2-oxoglutarate dehydrogenase complex | Q9I3D2 | 30 | 2 | 2 | 3.4 | Amino-acid degradation | Pseudomonas aeruginosa PAO1 | Bacteria; Proteobacteria; Gammaproteobacteria; Pseudomonadales; Pseudomonadaceae; Pseudomonas. |
| Coenzyme PQQ synthesis protein E | A5VXG4 | 30 | 3 | 1 | 2.1 | Cofactor biosynthesis | Pseudomonas putida F1 | Bacteria; Proteobacteria; Gammaproteobacteria; Pseudomonadales; Pseudomonadaceae; Pseudomonas. |
| Elongation factor G 2 | Q88FI4 | 19 | 2 | 2 | 3.4 | Translation | Pseudomonas putida KT2440 | Bacteria; Proteobacteria; Gammaproteobacteria; Pseudomonadales; Pseudomonadaceae; Pseudomonas. |
| 30S ribosomal protein S8 | B0KK81 | 19 | 2 | 1 | 8.5 | Ribosomes | Pseudomonas putida GB-1 | Bacteria; Proteobacteria; Gammaproteobacteria; Pseudomonadales; Pseudomonadaceae; Pseudomonas. |
| UDP-N-acetylglucosamine--N-acetylmuramyl-(pentapeptide) pyrophosphoryl-undecaprenol N-acetylglucosamine transferase | A4XQS4 | 18 | 2 | 2 | 8.7 | Out membrane proteins; Cell wall biogenesis | Pseudomonas mendocina ymp | Bacteria; Proteobacteria; Gammaproteobacteria; Pseudomonadales; Pseudomonadaceae; Pseudomonas. |
| Carbamoyl-phosphate synthase large chain | P38100 | 18 | 9 | 3 | 3.1 | Amino-acid biosynthesis | Pseudomonas aeruginosa PAO1 | Bacteria; Proteobacteria; Gammaproteobacteria; Pseudomonadales; Pseudomonadaceae; Pseudomonas. |
| Outer membrane protein Omp38 | A3M8K2 | 143 | 7 | 3 | 7.9 | Out membrane proteins | Acinetobacter baumannii ATCC 17978 | Bacteria; Proteobacteria; Gammaproteobacteria; Pseudomonadales; Moraxellaceae; Acinetobacter; Acinetobacter calcoaceticus/baumannii complex. |
| Trigger factor | B0V4R6 | 57 | 3 | 2 | 5.9 | Chaperone | Acinetobacter baumannii AYE | Bacteria; Proteobacteria; Gammaproteobacteria; Pseudomonadales; Moraxellaceae; Acinetobacter; Acinetobacter calcoaceticus/baumannii complex. |
| 50S ribosomal protein L17 | B3PK63 | 53 | 1 | 1 | 7.6 | Ribosomes | Cellvibrio japonicus Ueda107 | Bacteria; Proteobacteria; Gammaproteobacteria; Pseudomonadales; Pseudomonadaceae; Cellvibrio. |
| Acyl carrier protein | C1DRH0 | 40 | 2 | 2 | 32.1 | Lipid metabolism | Azotobacter vinelandii DJ | Bacteria; Proteobacteria; Gammaproteobacteria; Pseudomonadales; Pseudomonadaceae; Azotobacter. |
| 50S ribosomal protein L5 | C1DKM5 | 39 | 4 | 3 | 17.9 | Ribosomes | Azotobacter vinelandii DJ | Bacteria; Proteobacteria; Gammaproteobacteria; Pseudomonadales; Pseudomonadaceae; Azotobacter. |
| 50S ribosomal protein L23 | P0ADZ0 | 276 | 19 | 1 | 15 | Ribosomes | Escherichia coli K-12 | Bacteria; Proteobacteria; Gammaproteobacteria; Enterobacteriales; Enterobacteriaceae; Escherichia. |
| Major outer membrane lipoprotein Lpp | P69776 | 189 | 11 | 4 | 47.4 | Out membrane proteins | Escherichia coli K-12 | Bacteria; Proteobacteria; Gammaproteobacteria; Enterobacteriales; Enterobacteriaceae; Escherichia. |
| Maltose-binding periplasmic protein | P0AEX9 | 107 | 5 | 4 | 14.6 | Transport; Sugar transport | Escherichia coli K-12 | Bacteria; Proteobacteria; Gammaproteobacteria; Enterobacteriales; Enterobacteriaceae; Escherichia. |
| Putative amino-acid ABC transporter-binding protein YhdW | P45766 | 40 | 1 | 1 | 2.3 | Transport; Amino-acid transport | Escherichia coli K-12 | Bacteria; Proteobacteria; Gammaproteobacteria; Enterobacteriales; Enterobacteriaceae; Escherichia. |
| Uncharacterized protein YgaU | P0ADE6 | 28 | 1 | 1 | 5.4 | Other | Escherichia coli K-12 | Bacteria; Proteobacteria; Gammaproteobacteria; Enterobacteriales; Enterobacteriaceae; Escherichia. |
| Lysophospholipid transporter LplT | A4WE10 | 23 | 2 | 1 | 2.3 | Transport; Lipid transport | Enterobacter sp. 638 | Bacteria; Proteobacteria; Gammaproteobacteria; Enterobacteriales; Enterobacteriaceae; Enterobacter. |
| Glyceraldehyde-3-phosphate dehydrogenase | P0A1P0 | 50 | 3 | 2 | 6.3 | Carbohydrate metabolism; Glycolysis | Salmonella enterica subsp. enterica serovar Typhimurium str. LT2 | Bacteria; Proteobacteria; Gammaproteobacteria; Enterobacteriales; Enterobacteriaceae; Salmonella. |
| Flagellin | P06175 | 44 | 2 | 2 | 4.5 | Cell motility | Salmonella enterica subsp. enterica serovar Rubislaw | Bacteria; Proteobacteria; Gammaproteobacteria; Enterobacteriales; Enterobacteriaceae; Salmonella. |
| Endoribonuclease SymE | C0Q7I4 | 39 | 2 | 1 | 5.5 | RNA degradation | Salmonella enterica subsp. enterica serovar Paratyphi C strain RKS4594 | Bacteria; Proteobacteria; Gammaproteobacteria; Enterobacteriales; Enterobacteriaceae; Salmonella. |
| 30S ribosomal protein S14 | Q8K963 | 38 | 1 | 1 | 5.9 | Ribosomes | Buchnera aphidicola str. Sg (Schizaphis graminum) | Bacteria; Proteobacteria; Gammaproteobacteria; Enterobacteriales; Enterobacteriaceae; Buchnera. |
| Uncharacterized membrane protein BUsg_286 | Q8K9N7 | 37 | 6 | 2 | 3.6 | Other | Buchnera aphidicola str. Sg (Schizaphis graminum) | Bacteria; Proteobacteria; Gammaproteobacteria; Enterobacteriales; Enterobacteriaceae; Buchnera. |
| Chaperone protein HscA | Q7N228 | 25 | 2 | 1 | 2.8 | Chaperone | Photorhabdus luminescens subsp. laumondii TTO1 | Bacteria; Proteobacteria; Gammaproteobacteria; Enterobacteriales; Enterobacteriaceae; Photorhabdus. |
| Vitamin B12 transporter BtuB | Q7MYE3 | 22 | 3 | 1 | 1.5 | Transport | Photorhabdus luminescens subsp. laumondii TTO1 | Bacteria; Proteobacteria; Gammaproteobacteria; Enterobacteriales; Enterobacteriaceae; Photorhabdus. |
| Chaperone protein Skp | P31519 | 25 | 1 | 1 | 5.5 | Chaperone | Yersinia enterocolitica | Bacteria; Proteobacteria; Gammaproteobacteria; Enterobacteriales; Enterobacteriaceae; Yersinia. |
| H(+)/Cl(-) exchange transporter ClcA | B1JK21 | 16 | 2 | 1 | 1.5 | Transport; Ion transport | Yersinia pseudotuberculosis YPIII | Bacteria; Proteobacteria; Gammaproteobacteria; Enterobacteriales; Enterobacteriaceae; Yersinia. |
| Outer membrane protein A | P04845 | 72 | 4 | 4 | 14.5 | Out membrane proteins | Serratia marcescens | Bacteria; Proteobacteria; Gammaproteobacteria; Enterobacteriales; Enterobacteriaceae; Serratia. |
| Trigger factor | Q3IF53 | 142 | 10 | 8 | 21 | Chaperone | Pseudoalteromonas haloplanktis TAC125 | Bacteria; Proteobacteria; Gammaproteobacteria; Alteromonadales; Pseudoalteromonadaceae; Pseudoalteromonas. |
| Isocitrate dehydrogenase [NADP] 2 | P41561 | 75 | 1 | 1 | 2.3 | Carbohydrate metabolism; TCA | Colwellia maris | Bacteria; Proteobacteria; Gammaproteobacteria; Alteromonadales; Colwelliaceae; Colwellia. |
| Acetyl-coenzyme A synthetase | Q3IFM6 | 68 | 4 | 4 | 6.2 | Carbohydrate metabolism; Pyruvate metabolism | Pseudoalteromonas haloplanktis TAC125 | Bacteria; Proteobacteria; Gammaproteobacteria; Alteromonadales; Pseudoalteromonadaceae; Pseudoalteromonas. |
| 50S ribosomal protein L6 | B1KMW8 | 39 | 2 | 2 | 11.4 | Ribosomes | Shewanella woodyi ATCC 51908 | Bacteria; Proteobacteria; Gammaproteobacteria; Alteromonadales; Shewanellaceae; Shewanella. |
| 3-ketoacyl-CoA thiolase | Q3IJ24 | 31 | 3 | 1 | 4.9 | Lipid metabolism | Pseudoalteromonas haloplanktis TAC125 | Bacteria; Proteobacteria; Gammaproteobacteria; Alteromonadales; Pseudoalteromonadaceae; Pseudoalteromonas. |
| Phenylalanine--tRNA ligase alpha subunit | Q21KD6 | 17 | 2 | 1 | 3.3 | Translation | Saccharophagus degradans 2-40 | Bacteria; Proteobacteria; Gammaproteobacteria; Alteromonadales; Alteromonadaceae; Saccharophagus. |
| Ribosome-recycling factor | Q7VL83 | 69 | 2 | 2 | 9.2 | Translation | Haemophilus ducreyi 35000HP | Bacteria; Proteobacteria; Gammaproteobacteria; Pasteurellales; Pasteurellaceae; Haemophilus. |
| 30S ribosomal protein S6 | B0BQB3 | 54 | 2 | 2 | 16.1 | Ribosomes | Actinobacillus pleuropneumoniae serovar 3 str. JL03 | Bacteria; Proteobacteria; Gammaproteobacteria; Pasteurellales; Pasteurellaceae; Actinobacillus. |
| Malate dehydrogenase | A5UCQ1 | 38 | 2 | 2 | 7 | Carbohydrate metabolism; TCA | Haemophilus influenzae PittEE | Bacteria; Proteobacteria; Gammaproteobacteria; Pasteurellales; Pasteurellaceae; Haemophilus. |
| 50S ribosomal protein L19 | B0BSW1 | 24 | 2 | 1 | 7.8 | Ribosomes | Actinobacillus pleuropneumoniae serovar 3 str. JL03 | Bacteria; Proteobacteria; Gammaproteobacteria; Pasteurellales; Pasteurellaceae; Actinobacillus. |
| Na(+)/H(+) antiporter NhaB | A0KL08 | 38 | 3 | 1 | 1.3 | Transport; Ion transport | Aeromonas hydrophila subsp. hydrophila ATCC 7966 | Bacteria; Proteobacteria; Gammaproteobacteria; Aeromonadales; Aeromonadaceae; Aeromonas. |
| 50S ribosomal protein L3 | A0KF21 | 35 | 1 | 1 | 4.8 | Ribosomes | Aeromonas hydrophila subsp. hydrophila ATCC 7966 | Bacteria; Proteobacteria; Gammaproteobacteria; Aeromonadales; Aeromonadaceae; Aeromonas. |
| Integration host factor subunit alpha | Q0AAM1 | 34 | 2 | 2 | 18.8 | Replication and repair | Alkalilimnicola ehrlichii MLHE-1 | Bacteria; Proteobacteria; Gammaproteobacteria; Chromatiales; Ectothiorhodospiraceae; Alkalilimnicola. |
| 30S ribosomal protein S4 | A6W368 | 84 | 2 | 2 | 10.2 | Ribosomes | Marinomonas sp. MWYL1 | Bacteria; Proteobacteria; Gammaproteobacteria; Oceanospirillales; Marinomonas. |
| Chaperone protein ClpB | Q7MNK1 | 160 | 7 | 5 | 7.4 | Chaperone | Vibrio vulnificus YJ016 | Bacteria; Proteobacteria; Gammaproteobacteria; Vibrionales; Vibrionaceae; Vibrio. |
| Lipoyl synthase | B9JW84 | 35 | 2 | 2 | 4.3 | Translation; Protein modification | Agrobacterium vitis S4 | Bacteria; Proteobacteria; Alphaproteobacteria; Rhizobiales; Rhizobiaceae; Rhizobium/Agrobacterium group; Agrobacterium. |
| Sarcosine oxidase subunit alpha | O87386 | 37 | 2 | 1 | 0.6 | Amino acid metabolism | Sinorhizobium meliloti 1021 | Bacteria; Proteobacteria; Alphaproteobacteria; Rhizobiales; Rhizobiaceae; Sinorhizobium/Ensifer group; Sinorhizobium. |
| Citrate synthase | O33915 | 108 | 7 | 3 | 8.2 | Carbohydrate metabolism; TCA | Sinorhizobium meliloti 1021 | Bacteria; Proteobacteria; Alphaproteobacteria; Rhizobiales; Rhizobiaceae; Sinorhizobium/Ensifer group; Sinorhizobium. |
| Glycerol kinase | B1ZGW7 | 37 | 1 | 1 | 1.2 | Lipid metabolism | Methylobacterium populi BJ001 | Bacteria; Proteobacteria; Alphaproteobacteria; Rhizobiales; Methylobacteriaceae; Methylobacterium. |
| 50S ribosomal protein L19 | B0U8L6 | 20 | 4 | 2 | 16.8 | Ribosomes | Methylobacterium sp. 4-46 | Bacteria; Proteobacteria; Alphaproteobacteria; Rhizobiales; Methylobacteriaceae; Methylobacterium. |
| Histidine--tRNA ligase | Q987T0 | 27 | 3 | 2 | 3.8 | Translation | Mesorhizobium loti MAFF303099 | Bacteria; Proteobacteria; Alphaproteobacteria; Rhizobiales; Phyllobacteriaceae; Mesorhizobium. |
| Malate synthase G | Q2J0A5 | 103 | 10 | 5 | 7.6 | Carbohydrate metabolism; TCA | Rhodopseudomonas palustris HaA2 | Bacteria; Proteobacteria; Alphaproteobacteria; Rhizobiales; Bradyrhizobiaceae; Rhodopseudomonas. |
| Malate synthase G | C0RES0 | 73 | 5 | 3 | 2.5 | Carbohydrate metabolism; TCA | Brucella melitensis ATCC 23457 | Bacteria; Proteobacteria; Alphaproteobacteria; Rhizobiales; Brucellaceae; Brucella. |
| Ribosome maturation factor RimP | A7IC11 | 29 | 1 | 1 | 3.1 | Ribosome biogenesis | Xanthobacter autotrophicus Py2 | Bacteria; Proteobacteria; Alphaproteobacteria; Rhizobiales; Xanthobacteraceae; Xanthobacter. |
| Chromosomal replication initiator protein DnaA | A3PFL5 | 17 | 3 | 2 | 7.7 | Replication and repair | Rhodobacter sphaeroides ATCC 17029 | Bacteria; Proteobacteria; Alphaproteobacteria; Rhodobacterales; Rhodobacteraceae; Rhodobacter. |
| Trigger factor | B6ISY8 | 33 | 2 | 2 | 3.8 | Chaperone | Rhodospirillum centenum SW (Rhodocista centenaria SW) | Bacteria; Proteobacteria; Alphaproteobacteria; Rhodospirillales; Rhodospirillaceae; Rhodospirillum. |
| tRNA pseudouridine synthase A | Q2GGK1 | 36 | 3 | 1 | 2.4 | Translation | Ehrlichia chaffeensis str. Arkansas | Bacteria; Proteobacteria; Alphaproteobacteria; Rickettsiales; Anaplasmataceae; Ehrlichia. |
| Malate dehydrogenase | Q2GCH6 | 21 | 3 | 1 | 3.5 | Carbohydrate metabolism; TCA | Neorickettsia sennetsu str. Miyayama | Bacteria; Proteobacteria; Alphaproteobacteria; Rickettsiales; Anaplasmataceae; Neorickettsia. |
| p-hydroxybenzoic acid--AMP ligase FadD22 | B2HIL6 | 17 | 1 | 1 | 1.7 | Lipid metabolism | Mycobacterium marinum M | Bacteria; Actinobacteria; Actinobacteridae; Actinomycetales; Corynebacterineae; Mycobacteriaceae; Mycobacterium. |
| Malate synthase G | A1UGU7 | 72 | 1 | 1 | 1.8 | Carbohydrate metabolism; TCA | Mycobacterium sp. KMS | Bacteria; Actinobacteria; Actinobacteridae; Actinomycetales; Corynebacterineae; Mycobacteriaceae; Mycobacterium. |
| 2-phospho-L-lactate transferase | Q9CCK1 | 30 | 6 | 1 | 3 | Cofactor biosynthesis | Mycobacterium leprae TN | Bacteria; Actinobacteria; Actinobacteridae; Actinomycetales; Corynebacterineae; Mycobacteriaceae; Mycobacterium. |
| Thiamine-phosphate synthase | Q5YNP9 | 25 | 2 | 1 | 2.6 | Cofactor biosynthesis | Nocardia farcinica IFM 10152 | Bacteria; Actinobacteria; Actinobacteridae; Actinomycetales; Corynebacterineae; Nocardiaceae; Nocardia. |
| Probable DNA ligase | Q0SG75 | 28 | 1 | 1 | 2 | Replication and repair | Rhodococcus jostii RHA1 | Bacteria; Actinobacteria; Actinobacteridae; Actinomycetales; Corynebacterineae; Nocardiaceae; Rhodococcus. |
| Aspartyl/glutamyl-tRNA(Asn/Gln) amidotransferase subunit B | Q2J6U8 | 33 | 2 | 2 | 3.2 | Translation | Frankia sp. CcI3 | Bacteria; Actinobacteria; Actinobacteridae; Actinomycetales; Frankineae; Frankiaceae; Frankia. |
| Catalase | P29422 | 102 | 7 | 5 | 9.7 | Oxidative stress | Micrococcus luteus | Bacteria; Actinobacteria; Actinobacteridae; Actinomycetales; Micrococcineae; Micrococcaceae; Micrococcus. |
| Aminoglycoside N(3')-acetyltransferase IX | P29810 | 14 | 5 | 1 | 2.5 | Antibiotic resistance | Micromonospora chalcea | Bacteria; Actinobacteria; Actinobacteridae; Actinomycetales; Micromonosporineae; Micromonosporaceae; Micromonospora. |
| Imidazole glycerol phosphate synthase subunit HisF | Q6A7Y8 | 72 | 3 | 1 | 6 | Amino-acid biosynthesis | Propionibacterium acnes KPA171202 | Bacteria; Actinobacteria; Actinobacteridae; Actinomycetales; Propionibacterineae; Propionibacteriaceae; Propionibacterium. |
| ATP-dependent Clp protease ATP-binding subunit ClpX | B2JGL6 | 55 | 6 | 4 | 10.9 | Chaperone | Burkholderia phymatum STM815 | Bacteria; Proteobacteria; Betaproteobacteria; Burkholderiales; Burkholderiaceae; Burkholderia. |
| Elongation factor Tu | A4SUU7 | 31 | 5 | 5 | 13.9 | Translation | Polynucleobacter necessarius subsp. asymbioticus QLW-P1DMWA-1 | Bacteria; Proteobacteria; Betaproteobacteria; Burkholderiales; Burkholderiaceae; Polynucleobacter. |
| 2,3-dihydroxyphenylpropionate/2,3-dihydroxicinnamic acid 1,2-dioxygenase | Q9S157 | 32 | 2 | 1 | 2.8 | Aromatic compound metabolism | Comamonas testosteroni | Bacteria; Proteobacteria; Betaproteobacteria; Burkholderiales; Comamonadaceae; Comamonas. |
| UDP-N-acetylenolpyruvoylglucosamine reductase | Q3SMH1 | 35 | 1 | 1 | 2.5 | Out membrane proteins; Cell wall biogenesis | Thiobacillus denitrificans ATCC 25259 | Bacteria; Proteobacteria; Betaproteobacteria; Hydrogenophilales; Hydrogenophilaceae; Thiobacillus. |
| Bis(5'-nucleosyl)-tetraphosphatase, symmetrical | Q1H450 | 36 | 1 | 1 | 3.6 | Nucleotide metabolism | Methylobacillus flagellatus KT | Bacteria; Proteobacteria; Betaproteobacteria; Methylophilales; Methylophilaceae; Methylobacillus. |
| Lipoprotein signal peptidase | A1KS63 | 16 | 3 | 1 | 7.3 | Translation; Protein modification | Neisseria meningitidis FAM18 | Bacteria; Proteobacteria; Betaproteobacteria; Neisseriales; Neisseriaceae; Neisseria. |
| Ribosomal RNA large subunit methyltransferase H | Q47JQ1 | 28 | 4 | 1 | 6.4 | Ribosome biogenesis; rRNA processing | Dechloromonas aromatica RCB | Bacteria; Proteobacteria; Betaproteobacteria; Rhodocyclales; Rhodocyclaceae; Dechloromonas. |
| Chaperone protein HtpG | P61184 | 33 | 1 | 1 | 1 | Chaperone | Bdellovibrio bacteriovorus HD100 | Bacteria; Proteobacteria; Deltaproteobacteria; Bdellovibrionales; Bdellovibrionaceae; Bdellovibrio. |
| Alanine--tRNA ligase | Q6AQ16 | 33 | 1 | 1 | 0.8 | Translation | Desulfotalea psychrophila LSv54 | Bacteria; Proteobacteria; Deltaproteobacteria; Desulfobacterales; Desulfobulbaceae; Desulfotalea. |
| Glycine--tRNA ligase beta subunit | C4XS54 | 42 | 3 | 2 | 1.9 | Translation | Desulfovibrio magneticus RS-1 | Bacteria; Proteobacteria; Deltaproteobacteria; Desulfovibrionales; Desulfovibrionaceae; Desulfovibrio. |
| Trigger factor | Q0SMP7 | 46 | 1 | 1 | 1.8 | Chaperone | Borrelia afzelii Pko | Bacteria; Spirochaetes; Spirochaetales; Spirochaetaceae; Borrelia; Borrelia burgdorferi group. |
| Variable small protein 8 | Q45215 | 26 | 1 | 1 | 5.5 | Out membrane proteins | Borrelia hermsii | Bacteria; Spirochaetes; Spirochaetales; Spirochaetaceae; Borrelia. |
| tRNA dimethylallyltransferase | B0SBH8 | 25 | 7 | 1 | 4.1 | Translation | Leptospira biflexa serovar Patoc strain 'Patoc 1 (Ames)' | Bacteria; Spirochaetes; Spirochaetales; Leptospiraceae; Leptospira. |
| V-type ATP synthase subunit E | O51123 | 22 | 1 | 1 | 5 | Energy metabolism | Borrelia burgdorferi B31 | Bacteria; Spirochaetes; Spirochaetales; Spirochaetaceae; Borrelia; Borrelia burgdorferi group. |
| Cytadherence high molecular weight protein 2 | Q7NBF8 | 41 | 5 | 5 | 1.9 | Cell motility | Mycoplasma gallisepticum str. R(low) | Bacteria; Tenericutes; Mollicutes; Mycoplasmataceae; Mycoplasma. |
| Putative MgpC-like protein MPN_414 | P75372 | 30 | 3 | 2 | 5.5 | Other | Mycoplasma pneumoniae M129 | Bacteria; Tenericutes; Mollicutes; Mycoplasmataceae; Mycoplasma. |
| Uncharacterized protein MG323.1 | Q9ZB74 | 22 | 2 | 2 | 3.7 | Other | Mycoplasma genitalium G37 | Bacteria; Tenericutes; Mollicutes; Mycoplasmataceae; Mycoplasma. |
| DNA-directed RNA polymerase subunit beta' | Q9X6Y2 | 36 | 3 | 2 | 0.9 | Transcription | Aquifex pyrophilus | Bacteria; Aquificae; Aquificales; Aquificaceae; Aquifex. |
| Translation initiation factor IF-2 | O67825 | 25 | 3 | 3 | 3.9 | Translation | Aquifex aeolicus VF5 | Bacteria; Aquificae; Aquificales; Aquificaceae; Aquifex. |
| 50S ribosomal protein L5 | A5FMY8 | 34 | 7 | 2 | 9.8 | Ribosomes | Flavobacterium johnsoniae UW101 | Bacteria; Bacteroidetes; Flavobacteriia; Flavobacteriales; Flavobacteriaceae; Flavobacterium. |
| Ribosome-recycling factor | Q11XK1 | 22 | 2 | 1 | 6.5 | Translation | Cytophaga hutchinsonii ATCC 33406 | Bacteria; Bacteroidetes; Cytophagia; Cytophagales; Cytophagaceae; Cytophaga. |
| Elongation factor Tu | A2BT83 | 43 | 2 | 2 | 6 | Translation | Prochlorococcus marinus str. AS9601 | Bacteria; Cyanobacteria; Prochlorales; Prochlorococcaceae; Prochlorococcus. |
| LL-diaminopimelate aminotransferase | Q10ZC3 | 35 | 1 | 1 | 1.7 | Amino-acid biosynthesis | Trichodesmium erythraeum IMS101 | Bacteria; Cyanobacteria; Oscillatoriophycideae; Oscillatoriales; Trichodesmium. |
| S-adenosylmethionine synthase | B2UR09 | 32 | 1 | 1 | 2 | Amino-acid biosynthesis | Akkermansia muciniphila ATCC BAA-835 | Bacteria; Verrucomicrobia; Verrucomicrobiae; Verrucomicrobiales; Verrucomicrobiaceae; Akkermansia. |
| Catalase-peroxidase | B3E099 | 50 | 3 | 2 | 3.6 | Oxidative stress | Methylacidiphilum infernorum V4 | Bacteria; Verrucomicrobia; unclassified Verrucomicrobia; Methylacidiphilales; Methylacidiphilaceae; Methylacidiphilum. |
| Adenosylhomocysteinase | Q01VU1 | 35 | 2 | 1 | 1.7 | Amino-acid biosynthesis | Candidatus Solibacter usitatus Ellin6076 | Bacteria; Acidobacteria; Solibacteres; Solibacterales; Solibacteraceae; Candidatus Solibacter. |
| Ribonucleoside-diphosphate reductase subunit alpha | Q9PL93 | 17 | 7 | 3 | 3.7 | Replication and repair | Chlamydia muridarum Nigg | Bacteria; Chlamydiae; Chlamydiales; Chlamydiaceae; Chlamydia/Chlamydophila group; Chlamydia. |
| Single-stranded DNA-binding protein | Q9KH06 | 46 | 9 | 1 | 3.8 | Replication and repair | Thermus aquaticus | Bacteria; Deinococcus-Thermus; Deinococci; Thermales; Thermaceae; Thermus. |
| Elongation factor G | Q8R602 | 34 | 1 | 1 | 1.2 | Translation | Fusobacterium nucleatum subsp. nucleatum ATCC 25586 | Bacteria; Fusobacteria; Fusobacteriales; Fusobacteriaceae; Fusobacterium. |
| Adenine deaminase | C5CI63 | 21 | 4 | 1 | 2.1 | Nucleotide metabolism | Kosmotoga olearia TBF 19.5.1 | Bacteria; Thermotogae; Thermotogales; Thermotogaceae; Kosmotoga. |
| **Samle LS 2** | | | | | | | | |
| Flagellar basal-body rod protein FlgG | P23446 | 176 | 4 | 2 | 6.4 | Cell motility | Bacillus subtilis subsp. subtilis str. 168 | Bacteria; Firmicutes; Bacilli; Bacillales; Bacillaceae; Bacillus. |
| Putative flagellin YvzB | O34366 | 62 | 10 | 2 | 18.1 | Cell motility | Bacillus subtilis subsp. subtilis str. 168 | Bacteria; Firmicutes; Bacilli; Bacillales; Bacillaceae; Bacillus. |
| Endoglucanase | P10475 | 37 | 5 | 2 | 5 | Carbohydrate metabolism; Cellulose degradation | Bacillus subtilis subsp. subtilis str. 168 | Bacteria; Firmicutes; Bacilli; Bacillales; Bacillaceae; Bacillus. |
| tRNA(Ile)-lysidine synthase | P37563 | 34 | 3 | 2 | 4.2 | Translation | Bacillus subtilis subsp. subtilis str. 168 | Bacteria; Firmicutes; Bacilli; Bacillales; Bacillaceae; Bacillus. |
| Surfactin synthase subunit 2 | Q04747 | 33 | 2 | 2 | 0.7 | Antibiotic biosynthesis; surfactin biosynthesis | Bacillus subtilis subsp. subtilis str. 168 | Bacteria; Firmicutes; Bacilli; Bacillales; Bacillaceae; Bacillus. |
| Succinate dehydrogenase flavoprotein subunit | P08065 | 29 | 3 | 2 | 1.7 | Carbohydrate metabolism; TCA | Bacillus subtilis subsp. subtilis str. 168 | Bacteria; Firmicutes; Bacilli; Bacillales; Bacillaceae; Bacillus. |
| Transition state regulatory protein AbrB | P08874 | 28 | 1 | 1 | 8.3 | Transcription | Bacillus subtilis subsp. subtilis str. 168 | Bacteria; Firmicutes; Bacilli; Bacillales; Bacillaceae; Bacillus. |
| Aspartyl/glutamyl-tRNA(Asn/Gln) amidotransferase subunit B | Q5WJ19 | 27 | 2 | 1 | 1.9 | Translation | Bacillus clausii KSM-K16 | Bacteria; Firmicutes; Bacilli; Bacillales; Bacillaceae; Bacillus. |
| Phosphoserine aminotransferase | Q9KDM4 | 33 | 2 | 2 | 8 | Amino-acid biosynthesis | Bacillus halodurans C-125 | Bacteria; Firmicutes; Bacilli; Bacillales; Bacillaceae; Bacillus. |
| Chemoreceptor glutamine deamidase CheD | Q65JK2 | 25 | 4 | 1 | 6.5 | Cell motility | Bacillus licheniformis DSM 13 = ATCC 14580 | Bacteria; Firmicutes; Bacilli; Bacillales; Bacillaceae; Bacillus. |
| Probable glycine dehydrogenase [decarboxylating] subunit 1 | Q65HG0 | 28 | 3 | 2 | 5.1 | Amino acid metabolism | Bacillus licheniformis DSM 13 = ATCC 14580 | Bacteria; Firmicutes; Bacilli; Bacillales; Bacillaceae; Bacillus. |
| Protective antigen | P13423 | 68 | 6 | 2 | 2.1 | Immune system | Bacillus anthracis | Bacteria; Firmicutes; Bacilli; Bacillales; Bacillaceae; Bacillus; Bacillus cereus group. |
| Chaperone protein DnaK | A0RIT3 | 44 | 4 | 2 | 3.3 | Chaperone | Bacillus thuringiensis str. Al Hakam | Bacteria; Firmicutes; Bacilli; Bacillales; Bacillaceae; Bacillus; Bacillus cereus group. |
| ATP synthase subunit delta | A0RL98 | 29 | 2 | 1 | 5.6 | Energy metabolism | Bacillus thuringiensis str. Al Hakam | Bacteria; Firmicutes; Bacilli; Bacillales; Bacillaceae; Bacillus; Bacillus cereus group. |
| 2-succinyl-5-enolpyruvyl-6-hydroxy-3-cyclohexene-1-carboxylate synthase | A7GU91 | 18 | 1 | 1 | 1.7 | Cofactor biosynthesis | Bacillus cytotoxicus NVH 391-98 | Bacteria; Firmicutes; Bacilli; Bacillales; Bacillaceae; Bacillus; Bacillus cereus group. |
| Septation ring formation regulator EzrA | A9VKG1 | 15 | 1 | 1 | 1.9 | Cell growth and death | Bacillus weihenstephanensis KBAB4 | Bacteria; Firmicutes; Bacilli; Bacillales; Bacillaceae; Bacillus; Bacillus cereus group. |
| rRNA adenine N-6-methyltransferase | P02979 | 38 | 7 | 2 | 7 | Antibiotic resistance | Staphylococcus aureus | Bacteria; Firmicutes; Bacilli; Bacillales; Staphylococcus. |
| DNA repair protein RecO | Q2FGF7 | 25 | 3 | 2 | 6.4 | Replication and repair | Staphylococcus aureus subsp. aureus USA300 | Bacteria; Firmicutes; Bacilli; Bacillales; Staphylococcus. |
| Malonyl CoA-acyl carrier protein transacylase | Q5HGK3 | 19 | 2 | 1 | 4.9 | Lipid metabolism | Staphylococcus aureus subsp. aureus COL | Bacteria; Firmicutes; Bacilli; Bacillales; Staphylococcus. |
| Elongation factor Tu | A4IJI7 | 34 | 4 | 2 | 6.6 | Translation | Geobacillus thermodenitrificans NG80-2 | Bacteria; Firmicutes; Bacilli; Bacillales; Bacillaceae; Geobacillus. |
| 30S ribosomal protein S7 | A4IJI5 | 22 | 3 | 3 | 22.4 | Ribosomes | Geobacillus thermodenitrificans NG80-2 | Bacteria; Firmicutes; Bacilli; Bacillales; Bacillaceae; Geobacillus. |
| Uncharacterized protein Lin0241 | Q92F66 | 14 | 1 | 1 | 4.9 | Other | Listeria innocua Clip11262 | Bacteria; Firmicutes; Bacilli; Bacillales; Listeriaceae; Listeria. |
| 60 kDa chaperonin | B9E899 | 40 | 2 | 2 | 5.6 | Chaperone | Macrococcus caseolyticus JCSC5402 | Bacteria; Firmicutes; Bacilli; Bacillales; Macrococcus. |
| Phosphoglucosamine mutase | A8AWM5 | 22 | 1 | 1 | 2 | Carbohydrate metabolism; Amino sugar and nucleotide sugar metabolism | Streptococcus gordonii str. Challis | Bacteria; Firmicutes; Bacilli; Lactobacillales; Streptococcaceae; Streptococcus. |
| Beta-N-acetylhexosaminidase | P49610 | 21 | 2 | 2 | 1.4 | Carbohydrate metabolism; Amino sugar and nucleotide sugar metabolism | Streptococcus pneumoniae TIGR4 | Bacteria; Firmicutes; Bacilli; Lactobacillales; Streptococcaceae; Streptococcus. |
| DegV domain-containing protein SpyM3_1149 | P0DA54 | 19 | 2 | 1 | 3.9 | Other | Streptococcus pyogenes MGAS315 | Bacteria; Firmicutes; Bacilli; Lactobacillales; Streptococcaceae; Streptococcus. |
| Elongation factor Tu | A8YUS2 | 16 | 4 | 3 | 10.9 | Translation | Lactobacillus helveticus DPC 4571 | Bacteria; Firmicutes; Bacilli; Lactobacillales; Lactobacillaceae; Lactobacillus. |
| Isoleucine--tRNA ligase | Q1GAS8 | 47 | 4 | 1 | 0.8 | Translation | Lactobacillus delbrueckii subsp. bulgaricus ATCC 11842 | Bacteria; Firmicutes; Bacilli; Lactobacillales; Lactobacillaceae; Lactobacillus. |
| 50S ribosomal protein L29 | B1MW06 | 43 | 5 | 2 | 33.8 | Ribosomes | Leuconostoc citreum KM20 | Bacteria; Firmicutes; Bacilli; Lactobacillales; Leuconostoc. |
| Elongation factor Ts | Q04F87 | 43 | 2 | 2 | 8.2 | Translation | Oenococcus oeni PSU-1 | Bacteria; Firmicutes; Bacilli; Lactobacillales; Oenococcus. |
| Flagellin | P80583 | 242 | 8 | 4 | 8.8 | Cell motility | Clostridium tyrobutyricum | Bacteria; Firmicutes; Clostridia; Clostridiales; Clostridiaceae; Clostridium. |
| Probable 2-phosphosulfolactate phosphatase | Q899J5 | 26 | 3 | 1 | 5.2 | Dephosphorylation | Clostridium tetani E88 | Bacteria; Firmicutes; Clostridia; Clostridiales; Clostridiaceae; Clostridium. |
| Uracil phosphoribosyltransferase | B0S2A9 | 72 | 2 | 1 | 3.8 | Nucleotide metabolism | Finegoldia magna ATCC 29328 | Bacteria; Firmicutes; Clostridia; Clostridiales; Clostridiales Family XI. Incertae Sedis; Finegoldia. |
| Endonuclease MutS2 | A8MHU4 | 29 | 2 | 1 | 0.8 | Replication and repair | Alkaliphilus oremlandii OhILAs | Bacteria; Firmicutes; Clostridia; Clostridiales; Clostridiaceae; Alkaliphilus. |
| DNA-directed RNA polymerase subunit alpha | B8G1Z4 | 77 | 6 | 3 | 15.6 | Transcription | Desulfitobacterium hafniense DCB-2 | Bacteria; Firmicutes; Clostridia; Clostridiales; Peptococcaceae; Desulfitobacterium. |
| Elongation factor Tu | Q18CE4 | 86 | 6 | 3 | 10.6 | Translation | Clostridium difficile 630 | Bacteria; Firmicutes; Clostridia; Clostridiales; Peptostreptococcaceae. |
| Xylose isomerase | P19148 | 52 | 1 | 1 | 3 | Carbohydrate metabolism; Fructose and mannose metabolism | Thermoanaerobacterium thermosulfurigenes | Bacteria; Firmicutes; Clostridia; Thermoanaerobacterales; Thermoanaerobacterales Family III. Incertae Sedis; Thermoanaerobacterium. |
| Acetyl-coenzyme A synthetase 1 | Q9Z3R3 | 30 | 1 | 1 | 1.7 | Carbohydrate metabolism; Pyruvate metabolism | Sinorhizobium meliloti 1021 | Bacteria; Proteobacteria; Alphaproteobacteria; Rhizobiales; Rhizobiaceae; Sinorhizobium/Ensifer group; Sinorhizobium. |
| Pyruvate, phosphate dikinase | Q59754 | 43 | 3 | 2 | 2.3 | Carbohydrate metabolism; pyruvate metabolism | Sinorhizobium meliloti 1021 | Bacteria; Proteobacteria; Alphaproteobacteria; Rhizobiales; Rhizobiaceae; Sinorhizobium/Ensifer group; Sinorhizobium. |
| Coproporphyrinogen-III oxidase, aerobic | B3PV29 | 40 | 5 | 2 | 5.6 | Cofactor biosynthesis; Porphyrin biosynthesis | Rhizobium etli CIAT 652 | Bacteria; Proteobacteria; Alphaproteobacteria; Rhizobiales; Rhizobiaceae; Rhizobium/Agrobacterium group; Rhizobium. |
| Tyrosine recombinase XerC | Q8UC70 | 34 | 1 | 1 | 3.5 | Replication and repair | Agrobacterium fabrum str. C58 | Bacteria; Proteobacteria; Alphaproteobacteria; Rhizobiales; Rhizobiaceae; Rhizobium/Agrobacterium group; Agrobacterium; Agrobacterium tumefaciens complex. |
| Probable malate:quinone oxidoreductase | B3QK65 | 32 | 3 | 2 | 4.8 | Carbohydrate metabolism; TCA | Rhodopseudomonas palustris TIE-1 | Bacteria; Proteobacteria; Alphaproteobacteria; Rhizobiales; Bradyrhizobiaceae; Rhodopseudomonas. |
| 50S ribosomal protein L5 | Q2IXP8 | 29 | 2 | 1 | 5 | Ribosomes | Rhodopseudomonas palustris HaA2 | Bacteria; Proteobacteria; Alphaproteobacteria; Rhizobiales; Bradyrhizobiaceae; Rhodopseudomonas. |
| Glycerol-3-phosphate dehydrogenase [NAD(P)+] | B3Q866 | 16 | 12 | 1 | 4.6 | Lipid metabolism; Membrane lipid metabolism | Rhodopseudomonas palustris TIE-1 | Bacteria; Proteobacteria; Alphaproteobacteria; Rhizobiales; Bradyrhizobiaceae; Rhodopseudomonas. |
| Catalase-peroxidase | Q1QS58 | 23 | 4 | 2 | 3.6 | Oxidative stress | Nitrobacter hamburgensis X14 | Bacteria; Proteobacteria; Alphaproteobacteria; Rhizobiales; Bradyrhizobiaceae; Nitrobacter. |
| Translation initiation factor IF-2 | Q6G0P2 | 18 | 1 | 1 | 1.4 | Translation | Bartonella quintana str. Toulouse | Bacteria; Proteobacteria; Alphaproteobacteria; Rhizobiales; Bartonellaceae; Bartonella. |
| Chaperone protein HtpG | Q98JB6 | 20 | 4 | 2 | 4.6 | Chaperone | Mesorhizobium loti MAFF303099 | Bacteria; Proteobacteria; Alphaproteobacteria; Rhizobiales; Phyllobacteriaceae; Mesorhizobium. |
| 3-isopropylmalate dehydrogenase | Q2RV53 | 41 | 3 | 1 | 3.2 | Amino-acid biosynthesis | Rhodospirillum rubrum ATCC 11170 | Bacteria; Proteobacteria; Alphaproteobacteria; Rhodospirillales; Rhodospirillaceae; Rhodospirillum. |
| Glutamate--tRNA ligase 1 | B6ISG3 | 32 | 3 | 2 | 4.7 | Translation | Rhodospirillum centenum SW (Rhodocista centenaria SW) | Bacteria; Proteobacteria; Alphaproteobacteria; Rhodospirillales; Rhodospirillaceae; Rhodospirillum. |
| Histidine ammonia-lyase | A5FZB9 | 17 | 6 | 3 | 6.9 | Amino-acid degradation | Acidiphilium cryptum JF-5 | Bacteria; Proteobacteria; Alphaproteobacteria; Rhodospirillales; Acetobacteraceae; Acidiphilium. |
| Siroheme synthase | A9H259 | 32 | 5 | 1 | 3 | Cofactor biosynthesis | Gluconacetobacter diazotrophicus PAl 5 | Bacteria; Proteobacteria; Alphaproteobacteria; Rhodospirillales; Acetobacteraceae; Gluconacetobacter. |
| ATP synthase subunit beta 1 | A3PIB9 | 70 | 3 | 3 | 6.3 | Energy metabolism | Rhodobacter sphaeroides ATCC 17029 | Bacteria; Proteobacteria; Alphaproteobacteria; Rhodobacterales; Rhodobacteraceae; Rhodobacter. |
| [Protein-PII] uridylyltransferase | Q3J5H6 | 28 | 5 | 2 | 2.9 | Nitrogen fixation | Rhodobacter sphaeroides 2.4.1 | Bacteria; Proteobacteria; Alphaproteobacteria; Rhodobacterales; Rhodobacteraceae; Rhodobacter. |
| Succinate dehydrogenase flavoprotein subunit | Q59661 | 73 | 2 | 2 | 4 | Carbohydrate metabolism; TCA | Paracoccus denitrificans | Bacteria; Proteobacteria; Alphaproteobacteria; Rhodobacterales; Rhodobacteraceae; Paracoccus. |
| Macrolide export ATP-binding/permease protein MacB 1/2 | A1B677 | 43 | 2 | 2 | 2.5 | Transport; ABC transporter | Paracoccus denitrificans PD1222 | Bacteria; Proteobacteria; Alphaproteobacteria; Rhodobacterales; Rhodobacteraceae; Paracoccus. |
| Succinate dehydrogenase iron-sulfur subunit | Q1RGP3 | 66 | 1 | 1 | 4.6 | Carbohydrate metabolism; TCA | Rickettsia bellii RML369-C | Bacteria; Proteobacteria; Alphaproteobacteria; Rickettsiales; Rickettsiaceae; Rickettsieae; Rickettsia; belli group. |
| DNA-directed RNA polymerase subunit beta | A8GMA7 | 22 | 3 | 2 | 2 | Transcription | Rickettsia akari str. Hartford | Bacteria; Proteobacteria; Alphaproteobacteria; Rickettsiales; Rickettsiaceae; Rickettsieae; Rickettsia; spotted fever group. |
| Phosphoglucosamine mutase | Q5NNT4 | 89 | 3 | 1 | 1.6 | Carbohydrate metabolism; Amino sugar and nucleotide sugar metabolism | Zymomonas mobilis subsp. mobilis ZM4 = ATCC 31821 | Bacteria; Proteobacteria; Alphaproteobacteria; Sphingomonadales; Sphingomonadaceae; Zymomonas. |
| Ornithine carbamoyltransferase | B3R6D6 | 15 | 1 | 1 | 4.9 | Amino-acid degradation | Cupriavidus taiwanensis | Bacteria; Proteobacteria; Betaproteobacteria; Burkholderiales; Burkholderiaceae; Cupriavidus. |
| 30S ribosomal protein S6 | Q1LLW8 | 56 | 2 | 2 | 15.3 | Ribosomes | Cupriavidus metallidurans CH34 | Bacteria; Proteobacteria; Betaproteobacteria; Burkholderiales; Burkholderiaceae; Cupriavidus. |
| Aspartyl/glutamyl-tRNA(Asn/Gln) amidotransferase subunit C | Q1LSE0 | 40 | 1 | 1 | 9.1 | Translation | Cupriavidus metallidurans CH34 | Bacteria; Proteobacteria; Betaproteobacteria; Burkholderiales; Burkholderiaceae; Cupriavidus. |
| tRNA modification GTPase MnmE | Q8Y3H5 | 29 | 1 | 1 | 1.7 | Translation; tRNA processing | Ralstonia solanacearum GMI1000 | Bacteria; Proteobacteria; Betaproteobacteria; Burkholderiales; Burkholderiaceae; Ralstonia. |
| Putative NADH dehydrogenase/NAD(P)H nitroreductase HadB | Q53009 | 16 | 4 | 2 | 12.8 | Energy metabolism; Oxidative phosphorylation; Flavoprotein | Ralstonia pickettii | Bacteria; Proteobacteria; Betaproteobacteria; Burkholderiales; Burkholderiaceae; Ralstonia. |
| Azurin | P0A320 | 72 | 5 | 1 | 7.3 | Transport; Electron transport | Bordetella pertussis Tohama I | Bacteria; Proteobacteria; Betaproteobacteria; Burkholderiales; Alcaligenaceae; Bordetella. |
| Deoxyuridine 5'-triphosphate nucleotidohydrolase | A1V6V5 | 55 | 2 | 1 | 8.8 | Nucleotide metabolism; Pyrimidine metabolism | Burkholderia mallei SAVP1 | Bacteria; Proteobacteria; Betaproteobacteria; Burkholderiales; Burkholderiaceae; Burkholderia; pseudomallei group. |
| 3-deoxy-manno-octulosonate cytidylyltransferase | A4G7X9 | 36 | 2 | 1 | 3.2 | Outer membrane proteins; Bacterial outer membrane biogenesis | Herminiimonas arsenicoxydans | Bacteria; Proteobacteria; Betaproteobacteria; Burkholderiales; Oxalobacteraceae; Herminiimonas. |
| Malate dehydrogenase | C1DB66 | 103 | 3 | 2 | 8.5 | Carbohydrate metabolism; TCA | Laribacter hongkongensis HLHK9 | Bacteria; Proteobacteria; Betaproteobacteria; Neisseriales; Neisseriaceae; Laribacter. |
| Catalase | Q59602 | 27 | 1 | 1 | 3 | Oxidative stress | Neisseria gonorrhoeae | Bacteria; Proteobacteria; Betaproteobacteria; Neisseriales; Neisseriaceae; Neisseria. |
| D-amino acid dehydrogenase small subunit | A1KRK7 | 15 | 1 | 1 | 3.6 | Amino-acid degradation | Neisseria meningitidis FAM18 | Bacteria; Proteobacteria; Betaproteobacteria; Neisseriales; Neisseriaceae; Neisseria. |
| 30S ribosomal protein S2 | Q1H139 | 41 | 4 | 2 | 6.8 | Ribosomes | Methylobacillus flagellatus KT | Bacteria; Proteobacteria; Betaproteobacteria; Methylophilales; Methylophilaceae; Methylobacillus. |
| Protein translocase subunit SecA | Q1GZ36 | 23 | 6 | 3 | 2.5 | Transport; Protein transport | Methylobacillus flagellatus KT | Bacteria; Proteobacteria; Betaproteobacteria; Methylophilales; Methylophilaceae; Methylobacillus. |
| Full=60 kDa chaperonin | Q0AJH9 | 49 | 1 | 1 | 2.4 | Chaperone | Nitrosomonas eutropha C91 | Bacteria; Proteobacteria; Betaproteobacteria; Nitrosomonadales; Nitrosomonadaceae; Nitrosomonas. |
| 50S ribosomal protein L10 | Q82T73 | 34 | 1 | 1 | 3.5 | Ribosomes | Nitrosomonas europaea ATCC 19718 | Bacteria; Proteobacteria; Betaproteobacteria; Nitrosomonadales; Nitrosomonadaceae; Nitrosomonas. |
| 30S ribosomal protein S12 | Q8KTP6 | 34 | 3 | 2 | 11.9 | Ribosomes | Candidatus Tremblaya princeps | Bacteria; Proteobacteria; Betaproteobacteria; Candidatus Tremblaya. |
| Elongation factor Tu | Q1MPT8 | 101 | 4 | 3 | 7 | Translation | Lawsonia intracellularis PHE/MN1-00 | Bacteria; Proteobacteria; Deltaproteobacteria; Desulfovibrionales; Desulfovibrionaceae; Lawsonia. |
| Aspartyl/glutamyl-tRNA(Asn/Gln) amidotransferase subunit B | A1VCY2 | 21 | 2 | 1 | 3.4 | Translation | Desulfovibrio vulgaris DP4 | Bacteria; Proteobacteria; Deltaproteobacteria; Desulfovibrionales; Desulfovibrionaceae; Desulfovibrio. |
| Holliday junction ATP-dependent DNA helicase RuvA | B4UFY0 | 17 | 1 | 1 | 6.1 | Replication and repair | Anaeromyxobacter sp. K | Bacteria; Proteobacteria; Deltaproteobacteria; Myxococcales; Cystobacterineae; Myxococcaceae; Anaeromyxobacter. |
| Lon protease 2 | P36774 | 31 | 4 | 3 | 4.6 | Protease | Myxococcus xanthus | Bacteria; Proteobacteria; Deltaproteobacteria; Myxococcales; Cystobacterineae; Myxococcaceae; Myxococcus. |
| DNA mismatch repair protein MutS | A8ZXH5 | 20 | 1 | 1 | 1.2 | Replication and repair | Desulfococcus oleovorans Hxd3 | Bacteria; Proteobacteria; Deltaproteobacteria; Desulfobacterales; Desulfobacteraceae; Desulfococcus. |
| Methylthioribose-1-phosphate isomerase | Q3A2J8 | 21 | 1 | 1 | 2.6 | Amino-acid biosynthesis | Pelobacter carbinolicus DSM 2380 | Bacteria; Proteobacteria; Deltaproteobacteria; Desulfuromonadales; Pelobacteraceae; Pelobacter. |
| DNA mismatch repair protein MutL | Q2LUR5 | 87 | 26 | 2 | 3.6 | Replication and repair | Syntrophus aciditrophicus SB | Bacteria; Proteobacteria; Deltaproteobacteria; Syntrophobacterales; Syntrophaceae; Syntrophus. |
| Outer membrane protein A | P24017 | 200 | 14 | 6 | 15.4 | Outer membrane proteins | Klebsiella pneumoniae | Bacteria; Proteobacteria; Gammaproteobacteria; Enterobacteriales; Enterobacteriaceae; Klebsiella. |
| Outer membrane protein C | Q48473 | 150 | 9 | 4 | 12.1 | Outer membrane proteins | Klebsiella pneumoniae | Bacteria; Proteobacteria; Gammaproteobacteria; Enterobacteriales; Enterobacteriaceae; Klebsiella. |
| Fimbrial subunit type 3 | P12267 | 66 | 3 | 2 | 10.4 | Cell motility | Klebsiella pneumoniae | Bacteria; Proteobacteria; Gammaproteobacteria; Enterobacteriales; Enterobacteriaceae; Klebsiella. |
| Citrate lyase subunit beta | P17725 | 31 | 7 | 2 | 7.3 | Carbohydrate metabolism; TCA | Klebsiella pneumoniae | Bacteria; Proteobacteria; Gammaproteobacteria; Enterobacteriales; Enterobacteriaceae; Klebsiella. |
| Outer membrane protein X | P25253 | 230 | 10 | 5 | 32.6 | Outer membrane proteins | Enterobacter cloacae | Bacteria; Proteobacteria; Gammaproteobacteria; Enterobacteriales; Enterobacteriaceae; Enterobacter; Enterobacter cloacae complex. |
| Outer membrane pore protein E | Q47490 | 45 | 5 | 4 | 12.3 | Outer membrane proteins | Enterobacter cloacae | Bacteria; Proteobacteria; Gammaproteobacteria; Enterobacteriales; Enterobacteriaceae; Enterobacter; Enterobacter cloacae complex. |
| Maltoporin | A4W5F0 | 108 | 3 | 2 | 6.1 | Transport; Sugar transport | Enterobacter sp. 638 | Bacteria; Proteobacteria; Gammaproteobacteria; Enterobacteriales; Enterobacteriaceae; Enterobacter. |
| tRNA-dihydrouridine synthase C | Q8Z5B2 | 16 | 1 | 1 | 2.6 | Translation; tRNA processing | Salmonella enterica subsp. enterica serovar Typhi (Salmonella typhi) | Bacteria; Proteobacteria; Gammaproteobacteria; Enterobacteriales; Enterobacteriaceae; Salmonella. |
| Outer membrane protein S1 | Q56110 | 28 | 5 | 2 | 5.1 | Outer membrane proteins | Salmonella enterica subsp. enterica serovar Typhi (Salmonella typhi) | Bacteria; Proteobacteria; Gammaproteobacteria; Enterobacteriales; Enterobacteriaceae; Salmonella. |
| Glyceraldehyde-3-phosphate dehydrogenase | P0A1P0 | 64 | 2 | 1 | 4.2 | Carbohydrate degradation; glycolysis | Salmonella enterica subsp. enterica serovar Typhimurium str. LT2 | Bacteria; Proteobacteria; Gammaproteobacteria; Enterobacteriales; Enterobacteriaceae; Salmonella. |
| Glyoxylate/hydroxypyruvate reductase B | Q0W9V5 | 54 | 8 | 3 | 7.4 | Carbohydrate metabolism; Pyruvate metabolism; | Yersinia pestis | Bacteria; Proteobacteria; Gammaproteobacteria; Enterobacteriales; Enterobacteriaceae; Yersinia. |
| Urease subunit alpha | B1JR71 | 49 | 4 | 4 | 7.5 | Nitrogen metabolism; urea degradation | Yersinia pseudotuberculosis YPIII | Bacteria; Proteobacteria; Gammaproteobacteria; Enterobacteriales; Enterobacteriaceae; Yersinia. |
| Single-stranded DNA-binding protein | Q8K933 | 47 | 1 | 1 | 6.2 | Replication and repair | Buchnera aphidicola str. Sg (Schizaphis graminum) | Bacteria; Proteobacteria; Gammaproteobacteria; Enterobacteriales; Enterobacteriaceae; Buchnera. |
| Flagellar transcriptional regulator FlhC | B2VDW1 | 53 | 2 | 2 | 10.7 | Transcription regulation; Bacterial flagellum biogenesis | Erwinia tasmaniensis | Bacteria; Proteobacteria; Gammaproteobacteria; Enterobacteriales; Enterobacteriaceae; Erwinia. |
| 2-keto-3-deoxy-L-rhamnonate aldolase | Q0TFJ7 | 18 | 1 | 1 | 4.5 | Carbohydrate metabolism; Fructose and mannose metabolism | Escherichia coli 536 | Bacteria; Proteobacteria; Gammaproteobacteria; Enterobacteriales; Enterobacteriaceae; Escherichia. |
| Major outer membrane lipoprotein | P02940 | 63 | 1 | 1 | 15.4 | Outer membrane proteins | Morganella morganii | Bacteria; Proteobacteria; Gammaproteobacteria; Enterobacteriales; Enterobacteriaceae; Morganella. |
| Alkaline phosphatase L | P35482 | 102 | 2 | 1 | 3.3 | Dephosphorylation | Pseudomonas aeruginosa PAO1 | Bacteria; Proteobacteria; Gammaproteobacteria; Pseudomonadales; Pseudomonadaceae; Pseudomonas. |
| UPF0312 protein PST_3941 | A4VRG3 | 45 | 4 | 2 | 6.8 | Other | Pseudomonas stutzeri A1501 | Bacteria; Proteobacteria; Gammaproteobacteria; Pseudomonadales; Pseudomonadaceae; Pseudomonas. |
| Nicotinate dehydrogenase subunit B | Q88FX8 | 19 | 8 | 5 | 5.3 | Cofactor degradation | Pseudomonas putida KT2440 | Bacteria; Proteobacteria; Gammaproteobacteria; Pseudomonadales; Pseudomonadaceae; Pseudomonas. |
| Probable phosphite transport system-binding protein HtxB | O69061 | 24 | 1 | 1 | 4 | Transport; Signal | Pseudomonas stutzeri | Bacteria; Proteobacteria; Gammaproteobacteria; Pseudomonadales; Pseudomonadaceae; Pseudomonas. |
| Dihydroorotase | C1DQV1 | 28 | 2 | 2 | 6.3 | Nucleotide metabolism | Azotobacter vinelandii DJ | Bacteria; Proteobacteria; Gammaproteobacteria; Pseudomonadales; Pseudomonadaceae; Azotobacter. |
| Outer membrane protein Omp38 | A3M8K2 | 108 | 8 | 3 | 8.1 | Outer membrane proteins | Acinetobacter baumannii ATCC 17978 | Bacteria; Proteobacteria; Gammaproteobacteria; Pseudomonadales; Moraxellaceae; Acinetobacter; Acinetobacter calcoaceticus/baumannii complex. |
| Malate dehydrogenase | A5WGM2 | 227 | 7 | 3 | 15 | Carbohydrate metabolism; TCA | Psychrobacter sp. PRwf-1 | Bacteria; Proteobacteria; Gammaproteobacteria; Pseudomonadales; Moraxellaceae; Psychrobacter. |
| 50S ribosomal protein L1 | A1WVD2 | 35 | 2 | 1 | 3.5 | Ribosomes | Halorhodospira halophila SL1 | Bacteria; Proteobacteria; Gammaproteobacteria; Chromatiales; Ectothiorhodospiraceae; Halorhodospira. |
| 50S ribosomal protein L16 | A1WVB5 | 80 | 2 | 1 | 10.2 | Ribosomes | Halorhodospira halophila SL1 | Bacteria; Proteobacteria; Gammaproteobacteria; Chromatiales; Ectothiorhodospiraceae; Halorhodospira. |
| Chaperone protein DnaK | A1WX31 | 26 | 2 | 1 | 1.9 | Chaperone | Halorhodospira halophila SL1 | Bacteria; Proteobacteria; Gammaproteobacteria; Chromatiales; Ectothiorhodospiraceae; Halorhodospira. |
| Shikimate dehydrogenase | Q0A4S2 | 35 | 2 | 2 | 8.9 | Amino-acid biosynthesis | Alkalilimnicola ehrlichii MLHE-1 | Bacteria; Proteobacteria; Gammaproteobacteria; Chromatiales; Ectothiorhodospiraceae; Alkalilimnicola. |
| RNA-binding protein Hfq | Q0AB60 | 19 | 3 | 3 | 39.3 | Chaperone | Alkalilimnicola ehrlichii MLHE-1 | Bacteria; Proteobacteria; Gammaproteobacteria; Chromatiales; Ectothiorhodospiraceae; Alkalilimnicola. |
| 60 kDa chaperonin | B8GL19 | 32 | 1 | 1 | 4.8 | Chaperone | Thioalkalivibrio sulfidophilus HL-EbGr7 | Bacteria; Proteobacteria; Gammaproteobacteria; Chromatiales; Ectothiorhodospiraceae; Thioalkalivibrio. |
| Ketol-acid reductoisomerase | Q3J875 | 40 | 3 | 3 | 10.4 | Amino-acid biosynthesis | Nitrosococcus oceani ATCC 19707 | Bacteria; Proteobacteria; Gammaproteobacteria; Chromatiales; Chromatiaceae; Nitrosococcus. |
| 30S ribosomal protein S3 | Q2S918 | 35 | 3 | 3 | 11 | Ribosomes | Hahella chejuensis KCTC 2396 | Bacteria; Proteobacteria; Gammaproteobacteria; Oceanospirillales; Hahellaceae; Hahella. |
| 50S ribosomal protein L3 | Q2S912 | 30 | 1 | 1 | 3.8 | Ribosomes | Hahella chejuensis KCTC 2396 | Bacteria; Proteobacteria; Gammaproteobacteria; Oceanospirillales; Hahellaceae; Hahella. |
| DNA-directed RNA polymerase subunit beta | Q2S905 | 91 | 2 | 1 | 0.8 | Transcription | Hahella chejuensis KCTC 2396 | Bacteria; Proteobacteria; Gammaproteobacteria; Oceanospirillales; Hahellaceae; Hahella. |
| GTPase Der | Q0VNE4 | 39 | 1 | 1 | 1.5 | Ribosome biogenesis | Alcanivorax borkumensis SK2 | Bacteria; Proteobacteria; Gammaproteobacteria; Oceanospirillales; Alcanivoracaceae; Alcanivorax. |
| Ribonuclease P protein component | Q0VKU6 | 23 | 1 | 1 | 7.4 | Translation; tRNA processing | Alcanivorax borkumensis SK2 | Bacteria; Proteobacteria; Gammaproteobacteria; Oceanospirillales; Alcanivoracaceae; Alcanivorax. |
| 50S ribosomal protein L2 | A6W389 | 67 | 4 | 2 | 10.2 | Ribosomes | Marinomonas sp. MWYL1 | Bacteria; Proteobacteria; Gammaproteobacteria; Oceanospirillales; Marinomonas. |
| 1,4-alpha-glucan branching enzyme GlgB | B0TZI5 | 30 | 1 | 1 | 1.6 | Carbohydrate metabolism; Starch and sucrose metabolism ; Glycogen metabolism | Francisella philomiragia subsp. philomiragia ATCC 25017 | Bacteria; Proteobacteria; Gammaproteobacteria; Thiotrichales; Francisellaceae; Francisella. |
| Tryptophan synthase beta chain | Q0BP45 | 27 | 2 | 2 | 5.1 | Amino-acid biosynthesis | Francisella tularensis subsp. holarctica OSU18 | Bacteria; Proteobacteria; Gammaproteobacteria; Thiotrichales; Francisellaceae; Francisella. |
| 50S ribosomal protein L2 | Q31IX9 | 89 | 1 | 1 | 5.1 | Ribosomes | Thiomicrospira crunogena XCL-2 | Bacteria; Proteobacteria; Gammaproteobacteria; Thiotrichales; Piscirickettsiaceae; Thiomicrospira. |
| Na(+)/H(+) antiporter NhaB | A0KL08 | 36 | 4 | 1 | 1 | Transport; Ion transport | Aeromonas hydrophila subsp. hydrophila ATCC 7966 | Bacteria; Proteobacteria; Gammaproteobacteria; Aeromonadales; Aeromonadaceae; Aeromonas. |
| 30S ribosomal protein S11 | A0KF43 | 63 | 2 | 2 | 10.1 | Ribosomes | Aeromonas hydrophila subsp. hydrophila ATCC 7966 | Bacteria; Proteobacteria; Gammaproteobacteria; Aeromonadales; Aeromonadaceae; Aeromonas. |
| Bifunctional protein GlmU | Q21DL5 | 23 | 2 | 1 | 2.4 | Outer membrane proteins; Bacterial outer membrane biogenesis | Saccharophagus degradans 2-40 | Bacteria; Proteobacteria; Gammaproteobacteria; Alteromonadales; Alteromonadaceae; Saccharophagus. |
| Aspartate carbamoyltransferase | A1U761 | 18 | 2 | 1 | 3.6 | Nucleotide metabolism | Marinobacter aquaeolei VT8 | Bacteria; Proteobacteria; Gammaproteobacteria; Alteromonadales; Alteromonadaceae; Marinobacter. |
| Elongation factor Tu | Q605B0 | 330 | 11 | 4 | 8.3 | Translation | Methylococcus capsulatus str. Bath | Bacteria; Proteobacteria; Gammaproteobacteria; Methylococcales; Methylococcaceae; Methylococcus. |
| DNA-directed RNA polymerase subunit beta' | Q60A05 | 81 | 5 | 4 | 3.1 | Transcription | Methylococcus capsulatus str. Bath | Bacteria; Proteobacteria; Gammaproteobacteria; Methylococcales; Methylococcaceae; Methylococcus. |
| Protein translocase subunit SecA | A6VQX0 | 19 | 7 | 2 | 2.2 | Transport; Protein transport | Actinobacillus succinogenes 130Z | Bacteria; Proteobacteria; Gammaproteobacteria; Pasteurellales; Pasteurellaceae; Actinobacillus. |
| Uncharacterized protein HI_1419 | P44190 | 36 | 1 | 1 | 8.1 | Other | Haemophilus influenzae Rd KW20 | Bacteria; Proteobacteria; Gammaproteobacteria; Pasteurellales; Pasteurellaceae; Haemophilus. |
| Methionine--tRNA ligase | B0RWA1 | 19 | 2 | 2 | 4.2 | Translation | Xanthomonas campestris pv. campestris str. B100 | Bacteria; Proteobacteria; Gammaproteobacteria; Xanthomonadales; Xanthomonadaceae; Xanthomonas. |
| Undecaprenyl-diphosphatase | Q9PCE0 | 18 | 6 | 1 | 3.4 | Outer membrane proteins; Cell wall biogenesis/degradation | Xylella fastidiosa 9a5c | Bacteria; Proteobacteria; Gammaproteobacteria; Xanthomonadales; Xanthomonadaceae; Xylella. |
| 50S ribosomal protein L14 | B5ELY9 | 123 | 1 | 1 | 15.6 | Ribosomes | Acidithiobacillus ferrooxidans ATCC 53993 | Bacteria; Proteobacteria; Gammaproteobacteria; Acidithiobacillales; Acidithiobacillaceae; Acidithiobacillus. |
| ATP synthase subunit alpha | Q05FY3 | 44 | 3 | 2 | 4 | Energy metabolism | Candidatus Carsonella ruddii PV | Bacteria; Proteobacteria; Gammaproteobacteria; Candidatus Carsonella. |
| Acetyl-coenzyme A synthetase | Q9F7R5 | 141 | 4 | 3 | 4.1 | Carbohydrate metabolism; Pyruvate metabolism | uncultured marine gamma proteobacterium EBAC31A08 | Bacteria; Proteobacteria; Gammaproteobacteria; environmental samples. |
| Polyphosphate kinase | A4TE29 | 25 | 1 | 1 | 2.6 | Energy metabolism; Oxidative phosphorylation | Mycobacterium gilvum PYR-GCK | Bacteria; Actinobacteria; Actinobacteridae; Actinomycetales; Corynebacterineae; Mycobacteriaceae; Mycobacterium. |
| (Dimethylallyl)adenosine tRNA methylthiotransferase MiaB | Q49842 | 20 | 1 | 1 | 3.7 | Translation; tRNA processing | Mycobacterium leprae TN | Bacteria; Actinobacteria; Actinobacteridae; Actinomycetales; Corynebacterineae; Mycobacteriaceae; Mycobacterium. |
| Polyribonucleotide nucleotidyltransferase | A0QIW5 | 37 | 11 | 3 | 4.3 | mRNA degradation | Mycobacterium avium 104 | Bacteria; Actinobacteria; Actinobacteridae; Actinomycetales; Corynebacterineae; Mycobacteriaceae; Mycobacterium; Mycobacterium avium complex (MAC). |
| Putative S-adenosyl-L-methionine-dependent methyltransferase MAB_4584c | B1ML08 | 36 | 1 | 1 | 2 | Other | Mycobacterium abscessus ATCC 19977 | Bacteria; Actinobacteria; Actinobacteridae; Actinomycetales; Corynebacterineae; Mycobacteriaceae; Mycobacterium; Mycobacterium abscessus. |
| Chaperone protein DnaK | A4QHJ0 | 25 | 1 | 1 | 2.4 | Chaperone | Corynebacterium glutamicum R | Bacteria; Actinobacteria; Actinobacteridae; Actinomycetales; Corynebacterineae; Corynebacteriaceae; Corynebacterium. |
| (-)-trans-carveol dehydrogenase | Q9RA05 | 48 | 3 | 1 | 3.2 | Terpene metabolism | Rhodococcus erythropolis | Bacteria; Actinobacteria; Actinobacteridae; Actinomycetales; Corynebacterineae; Nocardiaceae; Rhodococcus. |
| Endoglucanase E-2 | P26222 | 38 | 4 | 3 | 7.9 | Carbohydrate metabolism; Cellulose degradation | Thermobifida fusca | Bacteria; Actinobacteria; Actinobacteridae; Actinomycetales; Streptosporangineae; Nocardiopsaceae; Thermobifida. |
| Carboxylesterase | Q47M62 | 37 | 1 | 1 | 2.6 | Xenobiotics biodegradation and metabolism; Drug metabolism | Thermobifida fusca YX | Bacteria; Actinobacteria; Actinobacteridae; Actinomycetales; Streptosporangineae; Nocardiopsaceae; Thermobifida. |
| N utilization substance protein B homolog | Q47QY5 | 20 | 3 | 1 | 7 | Transcription regulation; Transcription termination | Thermobifida fusca YX | Bacteria; Actinobacteria; Actinobacteridae; Actinomycetales; Streptosporangineae; Nocardiopsaceae; Thermobifida. |
| Endo-1,4-beta-xylanase B | P26515 | 64 | 3 | 1 | 2.7 | Carbohydrate metabolism; xylan degradation | Streptomyces lividans | Bacteria; Actinobacteria; Actinobacteridae; Actinomycetales; Streptomycineae; Streptomycetaceae; Streptomyces. |
| Phosphoenolpyruvate carboxykinase [GTP] | A8L175 | 21 | 3 | 2 | 3.3 | Carbohydrate biosynthesis; gluconeogenesis. | Frankia sp. EAN1pec | Bacteria; Actinobacteria; Actinobacteridae; Actinomycetales; Frankineae; Frankiaceae; Frankia. |
| Argininosuccinate synthase | Q6ACL1 | 44 | 3 | 2 | 4.2 | Amino-acid biosynthesis | Leifsonia xyli subsp. xyli str. CTCB07 | Bacteria; Actinobacteria; Actinobacteridae; Actinomycetales; Micrococcineae; Microbacteriaceae; Leifsonia. |
| Fructose-1,6-bisphosphatase class 1 | C0QTP7 | 36 | 8 | 1 | 2 | Carbohydrate biosynthesis; gluconeogenesis. | Persephonella marina EX-H1 | Bacteria; Aquificae; Aquificales; Hydrogenothermaceae; Persephonella. |
| Protein translocase subunit SecD | D0MIN4 | 51 | 2 | 1 | 1 | Transpotr; Protein transport | Rhodothermus marinus DSM 4252 | Bacteria; Bacteroidetes; Bacteroidetes Order II. Incertae sedis; Rhodothermaceae; Rhodothermus. |
| CinA-like protein | Q31CS5 | 22 | 1 | 1 | 2.1 | Metabolism of cofactors and vitamins | Prochlorococcus marinus str. MIT 9312 | Bacteria; Cyanobacteria; Prochlorales; Prochlorococcaceae; Prochlorococcus. |
| Adenosylhomocysteinase | A2C620 | 40 | 3 | 2 | 5 | Amino-acid biosynthesis | Prochlorococcus marinus str. MIT 9303 | Bacteria; Cyanobacteria; Prochlorales; Prochlorococcaceae; Prochlorococcus. |
| Photosystem I assembly protein Ycf4 | Q3MDS1 | 26 | 3 | 2 | 7.6 | Photosynthesis | Anabaena variabilis ATCC 29413 (Nostoc sp. PCC 7937) | Bacteria; Cyanobacteria; Nostocales; Nostocaceae; Anabaena. |
| Glutamyl-tRNA(Gln) amidotransferase subunit A | P73558 | 28 | 10 | 1 | 2.3 | Translation | Synechocystis sp. PCC 6803 substr. Kazusa | Bacteria; Cyanobacteria; Oscillatoriophycideae; Chroococcales; Synechocystis. |
| 3-isopropylmalate dehydrogenase | Q1IZK2 | 28 | 1 | 1 | 3.1 | Amino-acid biosynthesis | Deinococcus geothermalis DSM 11300 | Bacteria; Deinococcus-Thermus; Deinococci; Deinococcales; Deinococcaceae; Deinococcus. |
| Probable transcriptional regulatory protein GAU_0635 | C1A617 | 28 | 12 | 2 | 7.3 | Transcription regulation | Gemmatimonas aurantiaca T-27 | Bacteria; Gemmatimonadetes; Gemmatimonadales; Gemmatimonadaceae; Gemmatimonas. |
| Glutamate--tRNA ligase | B2S076 | 15 | 2 | 1 | 2.6 | Translation | Borrelia hermsii HS1 | Bacteria; Spirochaetes; Spirochaetales; Spirochaetaceae; Borrelia. |
| ATP-dependent protease ATPase subunit HslU | Q73NE3 | 28 | 1 | 1 | 1.6 | Chaperone | Treponema denticola ATCC 35405 | Bacteria; Spirochaetes; Spirochaetales; Spirochaetaceae; Treponema. |
| Aspartyl/glutamyl-tRNA(Asn/Gln) amidotransferase subunit B | P75533 | 17 | 2 | 2 | 4.2 | Translation | Mycoplasma pneumoniae M129 | Bacteria; Tenericutes; Mollicutes; Mycoplasmataceae; Mycoplasma. |
| 50S ribosomal protein L5 | Q98PZ4 | 34 | 1 | 1 | 4.4 | Ribosomes | Mycoplasma pulmonis UAB CTIP | Bacteria; Tenericutes; Mollicutes; Mycoplasmataceae; Mycoplasma. |
| Elongation factor Tu | Q98QG1 | 34 | 2 | 2 | 6.6 | Translation | Mycoplasma pulmonis UAB CTIP | Bacteria; Tenericutes; Mollicutes; Mycoplasmataceae; Mycoplasma. |
| Cytadherence high molecular weight protein 2 | Q7NBF8 | 34 | 11 | 6 | 2.8 | Cell motility | Mycoplasma gallisepticum str. R(low) | Bacteria; Tenericutes; Mollicutes; Mycoplasmataceae; Mycoplasma. |
| Segregation and condensation protein B | P75477 | 28 | 1 | 1 | 2.9 | Replication and repair | Mycoplasma pneumoniae M129 | Bacteria; Tenericutes; Mollicutes; Mycoplasmataceae; Mycoplasma. |
| ATP synthase subunit alpha | Q8EWZ0 | 52 | 4 | 3 | 6 | Energy metabolism | Mycoplasma penetrans HF-2 | Bacteria; Tenericutes; Mollicutes; Mycoplasmataceae; Mycoplasma. |
| Elongation factor G | P38525 | 42 | 3 | 2 | 3.6 | Translation | Thermotoga maritima MSB8 | Bacteria; Thermotogae; Thermotogales; Thermotogaceae; Thermotoga. |
| Ribosome maturation factor RimP | A5ILL0 | 36 | 2 | 2 | 12 | Ribosome biogenesis | Thermotoga petrophila RKU-1 | Bacteria; Thermotogae; Thermotogales; Thermotogaceae; Thermotoga. |
| **Samle LS 3** | | | | | | | | |
| **Protein** | **Accession** | **Score** | **Matches** | **Sequences** | **Sequence coverage** | **Function** | **Source** | **Organism** |
| Flagellar basal-body rod protein FlgG | P23446 | 187 | 3 | 1 | 6.4 | Cell motility | Bacillus subtilis subsp. subtilis str. 168 | Bacteria; Firmicutes; Bacilli; Bacillales; Bacillaceae; Bacillus. |
| Endoglucanase | P10475 | 35 | 4 | 1 | 1.6 | Carbohydrate metabolism; Cellulose degradation | Bacillus subtilis subsp. subtilis str. 168 | Bacteria; Firmicutes; Bacilli; Bacillales; Bacillaceae; Bacillus. |
| Succinate dehydrogenase flavoprotein subunit | P08065 | 22 | 4 | 3 | 5.3 | Carbohydrate metabolism; TCA | Bacillus subtilis subsp. subtilis str. 168 | Bacteria; Firmicutes; Bacilli; Bacillales; Bacillaceae; Bacillus. |
| Aryl-phospho-beta-D-glucosidase BglC | P42403 | 21 | 1 | 1 | 2.3 | Carbohydrate metabolism; Gluconeogenesis | Bacillus subtilis subsp. subtilis str. 168 | Bacteria; Firmicutes; Bacilli; Bacillales; Bacillaceae; Bacillus. |
| Arginine--tRNA ligase 2 | Q9KEL8 | 23 | 3 | 2 | 3.2 | Translation | Bacillus halodurans C-125 | Bacteria; Firmicutes; Bacilli; Bacillales; Bacillaceae; Bacillus. |
| Protective antigen | P13423 | 46 | 3 | 2 | 2.1 | Immune system | Bacillus anthracis | Bacteria; Firmicutes; Bacilli; Bacillales; Bacillaceae; Bacillus;  Bacillus cereus group. |
| Chaperone protein DnaK | A0RIT3 | 43 | 3 | 1 | 1.8 | Chaperone | Bacillus thuringiensis str. Al Hakam | Bacteria; Firmicutes; Bacilli; Bacillales; Bacillaceae; Bacillus;  Bacillus cereus group. |
| ATP synthase subunit delta | A0RL98 | 29 | 2 | 1 | 5.6 | Energy metabolism | Bacillus thuringiensis str. Al Hakam | Bacteria; Firmicutes; Bacilli; Bacillales; Bacillaceae; Bacillus;  Bacillus cereus group. |
| UPF0173 metal-dependent hydrolase BAMEG_4892 | C3L8Z4 | 28 | 1 | 1 | 6.6 | Other | Bacillus anthracis str. CDC 684 | Bacteria; Firmicutes; Bacilli; Bacillales; Bacillaceae; Bacillus;  Bacillus cereus group. |
| Septation ring formation regulator EzrA | A9VKG1 | 14 | 2 | 1 | 1.9 | Replication and repair | Bacillus weihenstephanensis KBAB4 | Bacteria; Firmicutes; Bacilli; Bacillales; Bacillaceae; Bacillus;  Bacillus cereus group. |
| Elongation factor Tu | A4IJI7 | 27 | 3 | 2 | 6.6 | Translation | Geobacillus thermodenitrificans NG80-2 | Bacteria; Firmicutes; Bacilli; Bacillales; Bacillaceae;  Geobacillus. |
| Malate synthase G | A4IN50 | 18 | 2 | 2 | 2 | Carbohydrate metabolism; TCA | Geobacillus thermodenitrificans NG80-2 | Bacteria; Firmicutes; Bacilli; Bacillales; Bacillaceae;  Geobacillus. |
| 50S ribosomal protein L29 | A0ALW0 | 26 | 1 | 1 | 20.6 | Ribosomes | Listeria welshimeri serovar 6b str. SLCC5334 | Bacteria; Firmicutes; Bacilli; Bacillales; Listeriaceae; Listeria. |
| rRNA adenine N-6-methyltransferase | P02979 | 48 | 6 | 2 | 10.7 | Transport | Staphylococcus aureus | Bacteria; Firmicutes; Bacilli; Bacillales; Staphylococcus. |
| DNA-directed RNA polymerase subunit beta' | P0C1T4 | 35 | 4 | 4 | 4.7 | Transcription | Staphylococcus aureus | Bacteria; Firmicutes; Bacilli; Bacillales; Staphylococcus. |
| Isoleucine--tRNA ligase | Q1GAS8 | 39 | 2 | 1 | 0.8 | Translation | Lactobacillus delbrueckii subsp. bulgaricus ATCC 11842 | Bacteria; Firmicutes; Bacilli; Lactobacillales; Lactobacillaceae;  Lactobacillus. |
| Chromosomal replication initiator protein DnaA | B3W6N4 | 38 | 2 | 1 | 1.8 | Replication and repair; Chromosome and associated proteins | Lactobacillus casei BL23 | Bacteria; Firmicutes; Bacilli; Lactobacillales; Lactobacillaceae;  Lactobacillus. |
| UDP-N-acetylenolpyruvoylglucosamine reductase | Q1GB18 | 28 | 1 | 1 | 4 | Outer membrane proteins; Cell wall biogenesis | Lactobacillus delbrueckii subsp. bulgaricus ATCC 11842 | Bacteria; Firmicutes; Bacilli; Lactobacillales; Lactobacillaceae;  Lactobacillus. |
| Glyceraldehyde-3-phosphate dehydrogenase | O32755 | 25 | 1 | 1 | 3.6 | Carbohydrate metabolism; Glycolysis | Lactobacillus delbrueckii subsp. bulgaricus | Bacteria; Firmicutes; Bacilli; Lactobacillales; Lactobacillaceae;  Lactobacillus. |
| ATP-dependent zinc metalloprotease FtsH | Q03Z46 | 19 | 3 | 2 | 2.4 | Proteases | Leuconostoc mesenteroides subsp. mesenteroides ATCC 8293 | Bacteria; Firmicutes; Bacilli; Lactobacillales; Leuconostoc. |
| DNA-binding protein HU | Q9CI64 | 24 | 1 | 1 | 16.5 | Replication and repair | Lactococcus lactis subsp. lactis Il1403 | Bacteria; Firmicutes; Bacilli; Lactobacillales; Streptococcaceae;  Lactococcus. |
| Flagellin | P80583 | 278 | 8 | 4 | 8.8 | Cell motility | Clostridium tyrobutyricum | Bacteria; Firmicutes; Clostridia; Clostridiales; Clostridiaceae;  Clostridium. |
| GTPase obg | A0Q1T4 | 40 | 2 | 1 | 1.9 | GTP-binding protein | Clostridium novyi NT | Bacteria; Firmicutes; Clostridia; Clostridiales; Clostridiaceae;  Clostridium. |
| Elongation factor Tu | A8MLC4 | 37 | 3 | 3 | 8.6 | Translation | Alkaliphilus oremlandii OhILAs | Bacteria; Firmicutes; Clostridia; Clostridiales; Clostridiaceae;  Alkaliphilus. |
| Uracil phosphoribosyltransferase | B0S2A9 | 45 | 1 | 1 | 3.8 | Nucleotide metabolism | Finegoldia magna ATCC 29328 | Bacteria; Firmicutes; Clostridia; Clostridiales; Clostridiales  Family XI. Incertae Sedis; Finegoldia. |
| Cysteine--tRNA ligase | A8MLB7 | 19 | 4 | 2 | 5.8 | Translation | Alkaliphilus oremlandii OhILAs | Bacteria; Firmicutes; Clostridia; Clostridiales; Clostridiaceae;  Alkaliphilus. |
| DNA-directed RNA polymerase subunit alpha | A4J139 | 65 | 4 | 1 | 2.2 | Transcription | Desulfotomaculum reducens MI-1 | Bacteria; Firmicutes; Clostridia; Clostridiales; Peptococcaceae;  Desulfotomaculum. |
| Elongation factor Tu | B8G1W4 | 57 | 3 | 2 | 5.8 | Translation | Desulfitobacterium hafniense DCB-2 | Bacteria; Firmicutes; Clostridia; Clostridiales; Peptococcaceae;  Desulfitobacterium. |
| Outer membrane protein X | P25253 | 262 | 9 | 5 | 32.6 | Outer membrane proteins | Enterobacter cloacae | Bacteria; Proteobacteria; Gammaproteobacteria; Enterobacteriales;  Enterobacteriaceae; Enterobacter; Enterobacter cloacae complex. |
| Outer membrane protein C | Q48473 | 222 | 15 | 7 | 25.3 | Outer membrane proteins | Klebsiella pneumoniae | Bacteria; Proteobacteria; Gammaproteobacteria; Enterobacteriales;  Enterobacteriaceae; Klebsiella. |
| Outer membrane protein A | P24017 | 194 | 12 | 6 | 16.3 | Outer membrane proteins | Klebsiella pneumoniae | Bacteria; Proteobacteria; Gammaproteobacteria; Enterobacteriales;  Enterobacteriaceae; Klebsiella. |
| Fimbrial subunit type 3 | P12267 | 52 | 4 | 2 | 10.4 | Cell motility | Klebsiella pneumoniae | Bacteria; Proteobacteria; Gammaproteobacteria; Enterobacteriales;  Enterobacteriaceae; Klebsiella. |
| Citrate lyase subunit beta | P17725 | 41 | 7 | 2 | 7.3 | Carbohydrate metabolism; TCA | Klebsiella pneumoniae | Bacteria; Proteobacteria; Gammaproteobacteria; Enterobacteriales;  Enterobacteriaceae; Klebsiella. |
| Maltoporin | C0Q4D6 | 55 | 3 | 2 | 6 | Transport; Sugar transport | Salmonella enterica subsp. enterica serovar Paratyphi C strain RKS4594 | Bacteria; Proteobacteria; Gammaproteobacteria; Enterobacteriales;  Enterobacteriaceae; Salmonella. |
| Outer membrane protein S2 | Q56111 | 27 | 1 | 1 | 5.2 | Outer membrane proteins | Salmonella enterica subsp. enterica serovar Typhi (Salmonella typhi) | Bacteria; Proteobacteria; Gammaproteobacteria; Enterobacteriales;  Enterobacteriaceae; Salmonella. |
| Single-stranded DNA-binding protein | Q8K933 | 34 | 1 | 1 | 6.2 | Replication and repair | Buchnera aphidicola str. Sg (Schizaphis graminum) | Bacteria; Proteobacteria; Gammaproteobacteria; Enterobacteriales;  Enterobacteriaceae; Buchnera. |
| Histidine biosynthesis bifunctional protein HisB | P59454 | 16 | 2 | 1 | 4.5 | Amino-acid biosynthesis | Buchnera aphidicola str. Bp (Baizongia pistaciae) | Bacteria; Proteobacteria; Gammaproteobacteria; Enterobacteriales;  Enterobacteriaceae; Buchnera. |
| Trigger factor | Q8K991 | 25 | 3 | 2 | 3.4 | Chaperone | Buchnera aphidicola str. Sg (Schizaphis graminum) | Bacteria; Proteobacteria; Gammaproteobacteria; Enterobacteriales;  Enterobacteriaceae; Buchnera. |
| 6-phosphofructokinase | A8GLB0 | 30 | 2 | 2 | 6.3 | Carbohydrate metabolism; Glycolysis | Serratia proteamaculans 568 | Bacteria; Proteobacteria; Gammaproteobacteria; Enterobacteriales;  Enterobacteriaceae; Serratia. |
| Flagellin | P13713 | 25 | 2 | 2 | 8 | Cell motility | Serratia marcescens | Bacteria; Proteobacteria; Gammaproteobacteria; Enterobacteriales;  Enterobacteriaceae; Serratia. |
| 1,4-alpha-glucan branching enzyme GlgB | A8GKV0 | 19 | 3 | 1 | 1.5 | Carbohydrate metabolism; Starch and sucrose metabolism ; Glycogen metabolism | Serratia proteamaculans 568 | Bacteria; Proteobacteria; Gammaproteobacteria; Enterobacteriales;  Enterobacteriaceae; Serratia. |
| 3-isopropylmalate dehydrogenase | Q493R1 | 20 | 1 | 1 | 3.6 | Amino-acid biosynthesis | Candidatus Blochmannia pennsylvanicus str. BPEN | Bacteria; Proteobacteria; Gammaproteobacteria; Enterobacteriales;  Enterobacteriaceae; ant endosymbionts; Candidatus Blochmannia. |
| Glyoxylate/hydroxypyruvate reductase B | Q0W9V5 | 52 | 8 | 2 | 4.9 | Carbohydrate metabolism; Pyruvate metabolism | Yersinia pestis | Bacteria; Proteobacteria; Gammaproteobacteria; Enterobacteriales;  Enterobacteriaceae; Yersinia. |
| Glyceraldehyde-3-phosphate dehydrogenase | P24750 | 45 | 2 | 2 | 7.8 | Carbohydrate metabolism; Glycolysis | Escherichia hermannii | Bacteria; Proteobacteria; Gammaproteobacteria; Enterobacteriales;  Enterobacteriaceae; Escherichia. |
| Major outer membrane lipoprotein | P02940 | 40 | 2 | 2 | 30.8 | Outer membrane proteins | Morganella morganii | Bacteria; Proteobacteria; Gammaproteobacteria; Enterobacteriales;  Enterobacteriaceae; Morganella. |
| 2-keto-3-deoxy-L-rhamnonate aldolase | Q31Z78 | 18 | 2 | 2 | 9.7 | Carbohydrate metabolism; Fructose and mannose metabolism | Shigella boydii Sb227 | Bacteria; Proteobacteria; Gammaproteobacteria; Enterobacteriales;  Enterobacteriaceae; Shigella. |
| UPF0312 protein PST_3941 | A4VRG3 | 43 | 4 | 2 | 6.8 | Other | Pseudomonas stutzeri A1501 | Bacteria; Proteobacteria; Gammaproteobacteria; Pseudomonadales;  Pseudomonadaceae; Pseudomonas. |
| Alkaline phosphatase L | P35482 | 75 | 2 | 1 | 3.3 | Dephosphorylation | Pseudomonas aeruginosa PAO1 | Bacteria; Proteobacteria; Gammaproteobacteria; Pseudomonadales;  Pseudomonadaceae; Pseudomonas. |
| Pyruvate dehydrogenase E1 component | Q59637 | 34 | 3 | 3 | 3.9 | Carbohydrate metabolism; Pyruvate metabolism | Pseudomonas aeruginosa PAO1 | Bacteria; Proteobacteria; Gammaproteobacteria; Pseudomonadales;  Pseudomonadaceae; Pseudomonas. |
| Probable M18 family aminopeptidase 2 | A4VJG1 | 19 | 1 | 1 | 3 | Proteases | Pseudomonas stutzeri A1501 | Bacteria; Proteobacteria; Gammaproteobacteria; Pseudomonadales;  Pseudomonadaceae; Pseudomonas. |
| Alanine--tRNA ligase | A4VJB3 | 18 | 3 | 3 | 4.5 | Translation | Pseudomonas stutzeri A1501 | Bacteria; Proteobacteria; Gammaproteobacteria; Pseudomonadales;  Pseudomonadaceae; Pseudomonas. |
| LPS-assembly protein LptD | Q4K4X8 | 16 | 4 | 3 | 3.3 | Outer membrane proteins; envelope biogenesis | Pseudomonas protegens Pf-5 | Bacteria; Proteobacteria; Gammaproteobacteria; Pseudomonadales;  Pseudomonadaceae; Pseudomonas. |
| Phenylalanine--tRNA ligase alpha subunit | B0KKR4 | 15 | 2 | 2 | 5 | Translation | Pseudomonas putida GB-1 | Bacteria; Proteobacteria; Gammaproteobacteria; Pseudomonadales;  Pseudomonadaceae; Pseudomonas. |
| Malate dehydrogenase | A5WGM2 | 221 | 6 | 3 | 15 | Carbohydrate metabolism; TCA | Psychrobacter sp. PRwf-1 | Bacteria; Proteobacteria; Gammaproteobacteria; Pseudomonadales; Moraxellaceae; Psychrobacter. |
| Uncharacterized protein in vnfD 5'region | P24423 | 40 | 1 | 1 | 1.7 | Other | Azotobacter chroococcum (strain mcd 1) | Bacteria; Proteobacteria; Gammaproteobacteria; Pseudomonadales;  Pseudomonadaceae; Azotobacter. |
| 50S ribosomal protein L2 | C1DKL6 | 27 | 3 | 3 | 10.6 | Ribosomes | Azotobacter vinelandii DJ | Bacteria; Proteobacteria; Gammaproteobacteria; Pseudomonadales;  Pseudomonadaceae; Azotobacter. |
| Outer membrane protein Omp38 | A3M8K2 | 88 | 8 | 3 | 8.1 | Outer membrane proteins | Acinetobacter baumannii ATCC 17978 | Bacteria; Proteobacteria; Gammaproteobacteria; Pseudomonadales;  Moraxellaceae; Acinetobacter; Acinetobacter calcoaceticus/baumannii complex. |
| 50S ribosomal protein L20 | B5EN54 | 33 | 2 | 2 | 14.3 | Ribosomes | Acidithiobacillus ferrooxidans ATCC 53993 | Bacteria; Proteobacteria; Gammaproteobacteria; Acidithiobacillales;  Acidithiobacillaceae; Acidithiobacillus. |
| 50S ribosomal protein L14 | B5ELY9 | 99 | 1 | 1 | 15.6 | Ribosomes | Acidithiobacillus ferrooxidans ATCC 53993 | Bacteria; Proteobacteria; Gammaproteobacteria; Acidithiobacillales;  Acidithiobacillaceae; Acidithiobacillus. |
| 30S ribosomal protein S11 | A0KF43 | 44 | 1 | 1 | 9.3 | Ribosomes | Aeromonas hydrophila subsp. hydrophila ATCC 7966 | Bacteria; Proteobacteria; Gammaproteobacteria; Aeromonadales;  Aeromonadaceae; Aeromonas. |
| 50S ribosomal protein L2 | A1S221 | 45 | 2 | 2 | 5.5 | Ribosomes | Shewanella amazonensis SB2B | Bacteria; Proteobacteria; Gammaproteobacteria; Alteromonadales;  Shewanellaceae; Shewanella. |
| 30S ribosomal protein S3 | A0KRN0 | 33 | 4 | 3 | 14.3 | Ribosomes | Shewanella sp. ANA-3 | Bacteria; Proteobacteria; Gammaproteobacteria; Alteromonadales;  Shewanellaceae; Shewanella. |
| 50S ribosomal protein L2 | Q5QXY0 | 20 | 6 | 3 | 12.8 | Ribosomes | Idiomarina loihiensis L2TR | Bacteria; Proteobacteria; Gammaproteobacteria; Alteromonadales;  Idiomarinaceae; Idiomarina. |
| DNA-directed RNA polymerase subunit beta' | A1WVC8 | 63 | 2 | 2 | 1.5 | Transcription | Halorhodospira halophila SL1 | Bacteria; Proteobacteria; Gammaproteobacteria; Chromatiales;  Ectothiorhodospiraceae; Halorhodospira. |
| 50S ribosomal protein L16 | A1WVB5 | 43 | 2 | 1 | 10.2 | Ribosomes | Halorhodospira halophila SL1 | Bacteria; Proteobacteria; Gammaproteobacteria; Chromatiales;  Ectothiorhodospiraceae; Halorhodospira. |
| Ketol-acid reductoisomerase | Q3J875 | 39 | 2 | 2 | 8.3 | Amino-acid biosynthesis | Nitrosococcus oceani ATCC 19707 | Bacteria; Proteobacteria; Gammaproteobacteria; Chromatiales;  Chromatiaceae; Nitrosococcus. |
| Chaperone protein DnaK | A1WX31 | 34 | 2 | 1 | 1.9 | Chaperone | Halorhodospira halophila SL1 | Bacteria; Proteobacteria; Gammaproteobacteria; Chromatiales;  Ectothiorhodospiraceae; Halorhodospira. |
| RNA-binding protein Hfq | Q0AB60 | 31 | 1 | 1 | 14.6 | Chaperone | Alkalilimnicola ehrlichii MLHE-1 | Bacteria; Proteobacteria; Gammaproteobacteria; Chromatiales;  Ectothiorhodospiraceae; Alkalilimnicola. |
| 50S ribosomal protein L14 | Q0ABG5 | 23 | 3 | 2 | 14.8 | Ribosomes | Alkalilimnicola ehrlichii MLHE-1 | Bacteria; Proteobacteria; Gammaproteobacteria; Chromatiales;  Ectothiorhodospiraceae; Alkalilimnicola. |
| ATP phosphoribosyltransferase | Q3J7H0 | 22 | 1 | 1 | 3.3 | Amino-acid biosynthesis | Nitrosococcus oceani ATCC 19707 | Bacteria; Proteobacteria; Gammaproteobacteria; Chromatiales;  Chromatiaceae; Nitrosococcus. |
| UDP-3-O-acylglucosamine N-acyltransferase | Q9AIP8 | 22 | 2 | 1 | 2.5 | Outer membrane proteins; Bacterial outer membrane biogenesis | Methylococcus capsulatus str. Bath | Bacteria; Proteobacteria; Gammaproteobacteria; Methylococcales;  Methylococcaceae; Methylococcus. |
| Elongation factor Tu | Q605B0 | 326 | 11 | 4 | 8.3 | Translation | Methylococcus capsulatus str. Bath | Bacteria; Proteobacteria; Gammaproteobacteria; Methylococcales;  Methylococcaceae; Methylococcus. |
| DNA-directed RNA polymerase subunit beta | Q2S905 | 62 | 2 | 1 | 0.8 | Transcription | Hahella chejuensis KCTC 2396 | Bacteria; Proteobacteria; Gammaproteobacteria; Oceanospirillales;  Hahellaceae; Hahella. |
| 50S ribosomal protein L3 | Q2S912 | 33 | 2 | 1 | 3.8 | Ribosomes | Hahella chejuensis KCTC 2396 | Bacteria; Proteobacteria; Gammaproteobacteria; Oceanospirillales;  Hahellaceae; Hahella. |
| Protein translocase subunit SecA | A6VQX0 | 23 | 7 | 2 | 2.5 | Transport; Protein transport | Actinobacillus succinogenes 130Z | Bacteria; Proteobacteria; Gammaproteobacteria; Pasteurellales;  Pasteurellaceae; Actinobacillus. |
| 50S ribosomal protein L2 | Q31IX9 | 58 | 2 | 2 | 7.6 | Ribosomes | Thiomicrospira crunogena XCL-2 | Bacteria; Proteobacteria; Gammaproteobacteria; Thiotrichales;  Piscirickettsiaceae; Thiomicrospira. |
| Outer membrane protein U | P0C6Q6 | 69 | 4 | 1 | 2.1 | Outer membrane proteins | Vibrio cholerae O1 biovar El Tor str. N16961 | Bacteria; Proteobacteria; Gammaproteobacteria; Vibrionales;  Vibrionaceae; Vibrio. |
| Nicotinate phosphoribosyltransferase | Q7MK41 | 15 | 1 | 1 | 2.1 | Metabolism of cofactors and vitamins | Vibrio vulnificus YJ016 | Bacteria; Proteobacteria; Gammaproteobacteria; Vibrionales;  Vibrionaceae; Vibrio. |
| 30S ribosomal protein S3 | B0U5K5 | 38 | 2 | 1 | 7.9 | Ribosomes | Xylella fastidiosa M12 | Bacteria; Proteobacteria; Gammaproteobacteria; Xanthomonadales;  Xanthomonadaceae; Xylella. |
| S-adenosylmethionine synthase | Q9PGB0 | 27 | 2 | 2 | 5.7 | Amino-acid biosynthesis | Xylella fastidiosa 9a5c | Bacteria; Proteobacteria; Gammaproteobacteria; Xanthomonadales;  Xanthomonadaceae; Xylella. |
| Undecaprenyl-diphosphatase | Q9PCE0 | 21 | 4 | 1 | 3.4 | Outer membrane proteins; Cell wall biogenesis/degradation | Xylella fastidiosa 9a5c | Bacteria; Proteobacteria; Gammaproteobacteria; Xanthomonadales;  Xanthomonadaceae; Xylella. |
| UDP-2,3-diacylglucosamine hydrolase | B2FQP4 | 57 | 1 | 1 | 5.6 | Lipid metabolism | Stenotrophomonas maltophilia K279a | Bacteria; Proteobacteria; Gammaproteobacteria; Xanthomonadales;  Xanthomonadaceae; Stenotrophomonas; Stenotrophomonas maltophilia group. |
| Acetyl-coenzyme A synthetase | Q9F7R5 | 83 | 2 | 1 | 2.8 | Carbohydrate metabolism;; Pyruvate metabolism | uncultured marine gamma proteobacterium EBAC31A08 | Bacteria; Proteobacteria; Gammaproteobacteria; environmental samples. |
| Sensor protein ChvG | P72292 | 35 | 4 | 1 | 1.9 | Carbohydrate metabolism; Glycan metabolism | Sinorhizobium meliloti 1021 | Bacteria; Proteobacteria; Alphaproteobacteria; Rhizobiales;  Rhizobiaceae; Sinorhizobium/Ensifer group; Sinorhizobium. |
| Acetyl-coenzyme A synthetase 1 | Q9Z3R3 | 24 | 2 | 1 | 1.7 | Carbohydrate metabolism;; Pyruvate metabolism | Sinorhizobium meliloti 1021 | Bacteria; Proteobacteria; Alphaproteobacteria; Rhizobiales;  Rhizobiaceae; Sinorhizobium/Ensifer group; Sinorhizobium. |
| Coproporphyrinogen-III oxidase, aerobic | B9J7B2 | 32 | 2 | 2 | 7.3 | Metabolism of cofactors and vitamins | Agrobacterium radiobacter K84 | Bacteria; Proteobacteria; Alphaproteobacteria; Rhizobiales;  Rhizobiaceae; Rhizobium/Agrobacterium group; Agrobacterium;  Agrobacterium tumefaciens complex. |
| Succinate dehydrogenase flavoprotein subunit | Q59661 | 89 | 2 | 2 | 4 | Carbohydrate metabolism; TCA | Paracoccus denitrificans | Bacteria; Proteobacteria; Alphaproteobacteria; Rhodobacterales; Rhodobacteraceae; Paracoccus. |
| 3-isopropylmalate dehydrogenase | Q2RV53 | 53 | 2 | 2 | 6.2 | Amino-acid biosynthesis | Rhodospirillum rubrum ATCC 11170 | Bacteria; Proteobacteria; Alphaproteobacteria; Rhodospirillales;  Rhodospirillaceae; Rhodospirillum. |
| Chromosome partition protein Smc | A3PMS2 | 24 | 5 | 4 | 4.2 | Replication and repair | Rhodobacter sphaeroides ATCC 17029 | Bacteria; Proteobacteria; Alphaproteobacteria; Rhodobacterales;  Rhodobacteraceae; Rhodobacter. |
| Phosphoglucosamine mutase | Q5NNT4 | 80 | 4 | 2 | 3.8 | Carbohydrate metabolism; Amino sugar and nucleotide sugar metabolism | Zymomonas mobilis subsp. mobilis ZM4 = ATCC 31821 | Bacteria; Proteobacteria; Alphaproteobacteria; Sphingomonadales;  Sphingomonadaceae; Zymomonas. |
| Malate dehydrogenase | B2JQD2 | 94 | 3 | 2 | 10.1 | Carbohydrate metabolism; TCA | Burkholderia phymatum STM815 | Bacteria; Proteobacteria; Betaproteobacteria; Burkholderiales; Burkholderiaceae; Burkholderia. |
| Azurin | P0A320 | 66 | 5 | 1 | 7.3 | Transport; Electron transport | Bordetella pertussis Tohama I | Bacteria; Proteobacteria; Betaproteobacteria; Burkholderiales;  Alcaligenaceae; Bordetella. |
| Deoxyuridine 5'-triphosphate nucleotidohydrolase | A1V6V5 | 39 | 1 | 1 | 8.8 | Nucleotide metabolism | Burkholderia mallei SAVP1 | Bacteria; Proteobacteria; Betaproteobacteria; Burkholderiales;  Burkholderiaceae; Burkholderia; pseudomallei group. |
| UPF0758 protein Bxeno_A3578 | Q13UX3 | 20 | 3 | 1 | 4.3 | Other | Burkholderia xenovorans LB400 | Bacteria; Proteobacteria; Betaproteobacteria; Burkholderiales;  Burkholderiaceae; Burkholderia. |
| ATP phosphoribosyltransferase regulatory subunit | Q1LLK0 | 16 | 1 | 1 | 1.6 | Amino-acid biosynthesis | Cupriavidus metallidurans CH34 | Bacteria; Proteobacteria; Betaproteobacteria; Burkholderiales;  Burkholderiaceae; Cupriavidus. |
| Protein translocase subunit SecA | Q1GZ36 | 33 | 4 | 1 | 1.2 | Transport; Protein transport | Methylobacillus flagellatus KT | Bacteria; Proteobacteria; Betaproteobacteria; Methylophilales;  Methylophilaceae; Methylobacillus. |
| 3-methyl-2-oxobutanoate hydroxymethyltransferase | Q1H3S0 | 31 | 2 | 1 | 2.7 | Metabolism of cofactors and vitamins | Methylobacillus flagellatus KT | Bacteria; Proteobacteria; Betaproteobacteria; Methylophilales;  Methylophilaceae; Methylobacillus. |
| 50S ribosomal protein L10 | Q82T73 | 34 | 1 | 1 | 3.5 | Ribosomes | Nitrosomonas europaea ATCC 19718 | Bacteria; Proteobacteria; Betaproteobacteria; Nitrosomonadales;  Nitrosomonadaceae; Nitrosomonas. |
| DNA mismatch repair protein MutL | Q2LUR5 | 53 | 26 | 2 | 2.8 | Replication and repair | Syntrophus aciditrophicus SB | Bacteria; Proteobacteria; Deltaproteobacteria; Syntrophobacterales;  Syntrophaceae; Syntrophus. |
| ATP synthase subunit beta 1 | Q3A605 | 46 | 4 | 3 | 7.7 | Energy metabolism | Pelobacter carbinolicus DSM 2380 | Bacteria; Proteobacteria; Deltaproteobacteria; Desulfuromonadales;  Pelobacteraceae; Pelobacter. |
| 50S ribosomal protein L11 | B5EFN9 | 23 | 1 | 1 | 10.7 | Ribosomes | Geobacter bemidjiensis Bem | Bacteria; Proteobacteria; Deltaproteobacteria; Desulfuromonadales;  Geobacteraceae; Geobacter. |
| DNA mismatch repair protein MutS | A8ZXH5 | 22 | 2 | 2 | 2.3 | Replication and repair | Desulfococcus oleovorans Hxd3 | Bacteria; Proteobacteria; Deltaproteobacteria; Desulfobacterales;  Desulfobacteraceae; Desulfococcus. |
| Aspartyl/glutamyl-tRNA(Asn/Gln) amidotransferase subunit B | Q2IH95 | 18 | 1 | 1 | 1.9 | Translation | Anaeromyxobacter dehalogenans 2CP-C | Bacteria; Proteobacteria; Deltaproteobacteria; Myxococcales;  Cystobacterineae; Myxococcaceae; Anaeromyxobacter. |
| Chaperone protein ClpB | Q6NF05 | 43 | 2 | 2 | 2.1 | Chaperone | Corynebacterium diphtheriae NCTC 13129 | Bacteria; Actinobacteria; Actinobacteridae; Actinomycetales; Corynebacterineae; Corynebacteriaceae; Corynebacterium. |
| NADH-quinone oxidoreductase subunit D | A0QU33 | 24 | 2 | 2 | 7 | Energy metabolism | Mycobacterium smegmatis str. MC2 155 | Bacteria; Actinobacteria; Actinobacteridae; Actinomycetales; Corynebacterineae; Mycobacteriaceae; Mycobacterium. |
| 3-(3-hydroxy-phenyl)propionate/3-hydroxycinnamic acid hydroxylase | A4T8B6 | 22 | 3 | 3 | 5.3 | Degradation of aromatic compounds | Mycobacterium gilvum PYR-GCK | Bacteria; Actinobacteria; Actinobacteridae; Actinomycetales; Corynebacterineae; Mycobacteriaceae; Mycobacterium. |
| Thymidylate synthase | A1KMA6 | 39 | 1 | 1 | 4.9 | Nucleotide metabolism | Mycobacterium bovis BCG str. Pasteur 1173P2 | Bacteria; Actinobacteria; Actinobacteridae; Actinomycetales; Corynebacterineae; Mycobacteriaceae; Mycobacterium; Mycobacterium tuberculosis complex. |
| Polyribonucleotide nucleotidyltransferase | A0QIW5 | 25 | 11 | 3 | 3.3 | mRNA degradation | Mycobacterium avium 104 | Bacteria; Actinobacteria; Actinobacteridae; Actinomycetales; Corynebacterineae; Mycobacteriaceae; Mycobacterium; Mycobacterium avium complex (MAC). |
| Histidine--tRNA ligase | A1KLS8 | 34 | 2 | 2 | 3.8 | Translation | Mycobacterium bovis BCG str. Pasteur 1173P2 | Bacteria; Actinobacteria; Actinobacteridae; Actinomycetales; Corynebacterineae; Mycobacteriaceae; Mycobacterium; Mycobacterium tuberculosis complex. |
| (-)-trans-carveol dehydrogenase | Q9RA05 | 49 | 2 | 1 | 3.2 | Metabolism of terpenoids and polyketides; Terpene metabolism | Rhodococcus erythropolis | Bacteria; Actinobacteria; Actinobacteridae; Actinomycetales; Corynebacterineae; Nocardiaceae; Rhodococcus. |
| Probable cytosol aminopeptidase | Q0SHL0 | 41 | 1 | 1 | 1.4 | Proteases | Rhodococcus jostii RHA1 | Bacteria; Actinobacteria; Actinobacteridae; Actinomycetales; Corynebacterineae; Nocardiaceae; Rhodococcus. |
| Endo-1,4-beta-xylanase B | P26515 | 65 | 4 | 2 | 5.4 | Carbohydrate metabolism; xylan degradation | Streptomyces lividans | Bacteria; Actinobacteria; Actinobacteridae; Actinomycetales; Streptomycineae; Streptomycetaceae; Streptomyces. |
| Carboxylesterase | Q47M62 | 48 | 1 | 1 | 2.6 | Xenobiotics biodegradation and metabolism; Drug metabolism | Thermobifida fusca YX | Bacteria; Actinobacteria; Actinobacteridae; Actinomycetales; Streptosporangineae; Nocardiopsaceae; Thermobifida. |
| Endoglucanase E-2 | P26222 | 32 | 1 | 1 | 2.7 | Carbohydrate metabolism; Cellulose degradation | Thermobifida fusca | Bacteria; Actinobacteria; Actinobacteridae; Actinomycetales; Streptosporangineae; Nocardiopsaceae; Thermobifida. |
| DNA-directed RNA polymerase subunit beta' | A8M538 | 32 | 2 | 2 | 2 | Transcription | Salinispora arenicola CNS-205 | Bacteria; Actinobacteria; Actinobacteridae; Actinomycetales; Micromonosporineae; Micromonosporaceae; Salinispora. |
| ATP synthase subunit alpha | A4XAW4 | 47 | 3 | 3 | 5.6 | Energy metabolism | Salinispora tropica CNB-440 | Bacteria; Actinobacteria; Actinobacteridae; Actinomycetales; Micromonosporineae; Micromonosporaceae; Salinispora. |
| 60 kDa chaperonin 2 | A4FPA5 | 45 | 3 | 3 | 4.5 | Chaperone | Saccharopolyspora erythraea NRRL 2338 | Bacteria; Actinobacteria; Actinobacteridae; Actinomycetales; Pseudonocardineae; Pseudonocardiaceae; Saccharopolyspora. |
| Phosphoenolpyruvate carboxykinase [GTP] | A8L175 | 32 | 2 | 1 | 2.1 | Carbohydrate metabolism; gluconeogenesis | Frankia sp. EAN1pec | Bacteria; Actinobacteria; Actinobacteridae; Actinomycetales; Frankineae; Frankiaceae; Frankia. |
| Aspartyl/glutamyl-tRNA(Asn/Gln) amidotransferase subunit B | Q2J6U8 | 26 | 3 | 1 | 1.6 | Translation | Frankia sp. CcI3 | Bacteria; Actinobacteria; Actinobacteridae; Actinomycetales; Frankineae; Frankiaceae; Frankia. |
| tRNA (guanosine(18)-2'-O)-methyltransferase | O67577 | 18 | 2 | 1 | 6.2 | Translation; tRNA processing | Aquifex aeolicus VF5 | Bacteria; Aquificae; Aquificales; Aquificaceae; Aquifex. |
| 50S ribosomal protein L9 | B4U7M7 | 38 | 3 | 2 | 7.4 | Ribosomes | Hydrogenobaculum sp. Y04AAS1 | Bacteria; Aquificae; Aquificales; Aquificaceae; Hydrogenobaculum. |
| Gamma-glutamyl phosphate reductase | Q5LI24 | 40 | 1 | 1 | 2.2 | Amino-acid biosynthesis | Bacteroides fragilis NCTC 9343 | Bacteria; Bacteroidetes; Bacteroidia; Bacteroidales;  Bacteroidaceae; Bacteroides. |
| Elongation factor G | A0M5A0 | 27 | 4 | 4 | 7.3 | Translation | Gramella forsetii KT0803 | Bacteria; Bacteroidetes; Flavobacteriia; Flavobacteriales;  Flavobacteriaceae; Gramella. |
| Protein translocase subunit SecD | D0MIN4 | 54 | 2 | 1 | 1 | Transport; Protein transport | Rhodothermus marinus DSM 4252 | Bacteria; Bacteroidetes; Bacteroidetes Order II. Incertae sedis;  Rhodothermaceae; Rhodothermus. |
| 30S ribosomal protein S12 | Q2S3R9 | 16 | 3 | 1 | 9.7 | Ribosomes | Salinibacter ruber DSM 13855 | Bacteria; Bacteroidetes; Bacteroidetes Order II. Incertae sedis;  Rhodothermaceae; Salinibacter. |
| Membrane protein insertase YidC | Q11S04 | 40 | 2 | 2 | 2.4 | Outer membrane proteins | Cytophaga hutchinsonii ATCC 33406 | Bacteria; Bacteroidetes; Cytophagia; Cytophagales; Cytophagaceae;  Cytophaga. |
| Proton extrusion protein PcxA | B1XN58 | 25 | 1 | 1 | 3 | Transport; Ion transport | Synechococcus sp. PCC 7002 | Bacteria; Cyanobacteria; Oscillatoriophycideae; Chroococcales;  Synechococcus. |
| Adaptive-response sensory-kinase SasA | Q06904 | 21 | 2 | 2 | 9.8 | Signal transduction | Synechococcus elongatus PCC 7942 | Bacteria; Cyanobacteria; Oscillatoriophycideae; Chroococcales;  Synechococcus. |
| Putative ribosome biogenesis GTPase RsgA | Q0IE58 | 20 | 1 | 1 | 3.6 | Ribosome biogenesis | Synechococcus sp. CC9311 | Bacteria; Cyanobacteria; Oscillatoriophycideae; Chroococcales;  Synechococcus. |
| 50S ribosomal protein L20 | Q114D6 | 17 | 1 | 1 | 6.8 | Ribosomes | Trichodesmium erythraeum IMS101 | Bacteria; Cyanobacteria; Oscillatoriophycideae; Oscillatoriales;  Trichodesmium. |
| Adenosylhomocysteinase | A2C620 | 32 | 3 | 3 | 6.9 | Amino acid metabolism | Prochlorococcus marinus str. MIT 9303 | Bacteria; Cyanobacteria; Prochlorales; Prochlorococcaceae;  Prochlorococcus. |
| CinA-like protein | Q31CS5 | 21 | 2 | 2 | 5.7 | Metabolism of cofactors and vitamins | Prochlorococcus marinus str. MIT 9312 | Bacteria; Cyanobacteria; Prochlorales; Prochlorococcaceae;  Prochlorococcus. |
| Probable transcriptional regulatory protein GAU_0635 | C1A617 | 22 | 15 | 1 | 4.5 | Transcription | Gemmatimonas aurantiaca T-27 | Bacteria; Gemmatimonadetes; Gemmatimonadales; Gemmatimonadaceae;  Gemmatimonas. |
| DNA-directed RNA polymerase subunit beta | Q7URW6 | 34 | 2 | 2 | 1 | Transcription | Rhodopirellula baltica SH 1 | Bacteria; Planctomycetes; Planctomycetia; Planctomycetales;  Planctomycetaceae; Rhodopirellula. |
| Elongation factor Tu | B2S0H9 | 19 | 1 | 1 | 4.1 | Translation | Borrelia hermsii HS1 | Bacteria; Spirochaetes; Spirochaetales; Spirochaetaceae; Borrelia. |
| ATP synthase subunit alpha | Q8EWZ0 | 43 | 3 | 2 | 4.9 | Energy metabolism | Mycoplasma penetrans HF-2 | Bacteria; Tenericutes; Mollicutes; Mycoplasmataceae; Mycoplasma. |
| Cytadherence high molecular weight protein 2 | Q7NBF8 | 36 | 9 | 6 | 3.2 | Cell motility | Mycoplasma gallisepticum str. R(low) | Bacteria; Tenericutes; Mollicutes; Mycoplasmataceae; Mycoplasma. |
| 50S ribosomal protein L5 | Q98PZ4 | 28 | 1 | 1 | 4.4 | Ribosomes | Mycoplasma pulmonis UAB CTIP | Bacteria; Tenericutes; Mollicutes; Mycoplasmataceae; Mycoplasma. |
| Ribosomal RNA small subunit methyltransferase H | B3R0Q2 | 30 | 1 | 1 | 3 | Ribosome biogenesis; rRNA processing | Candidatus Phytoplasma mali AT | Bacteria; Tenericutes; Mollicutes; Acholeplasmatales; Acholeplasmataceae; Candidatus Phytoplasma; 16SrX (Apple proliferation group). |
| Elongation factor G | P38525 | 55 | 1 | 1 | 1 | Translation | Thermotoga maritima MSB8 | Bacteria; Thermotogae; Thermotogales; Thermotogaceae; Thermotoga. |
| Uracil phosphoribosyltransferase | B1L7Y5 | 19 | 1 | 1 | 5.7 | Nucleotide metabolism | Thermotoga sp. RQ2 | Bacteria; Thermotogae; Thermotogales; Thermotogaceae; Thermotoga. |
