# Supplementary material for: Metaproteomics reveals major microbial players and their biodegradation functions in a large-scale aerobic composting plant
Source: Microb Biotechnol. 2015 May 19;8(6):950–60. doi: 10.1111/1751-7915.12290 (PMC4621448; doi:10.1111/1751-7915.12290)
Supplement: Supplementary file 1 — Table S1. Detailed identifications of bacterial proteins extracted from samples. Table S2. Detailed identifications of fungal proteins extracted from samples. [file mbt20008-0950-sd1.zip › MBT2_12290_supp-0002-SupplementaryTableS2.doc]

**Supplementary Table S2 Detailed identifications of fungal proteins extracted from samples**

| **Protein** | **Accession** | **Score** | **Assigned spetra** | **Unique peptides** | **Sequence coverage** | **Function** | **Source** | **Organism** |
| --- | --- | --- | --- | --- | --- | --- | --- | --- |
| **Samle LS 1** | | | | | | | | |
| Succinate dehydrogenase [ubiquinone] flavoprotein subunit, mitochondrial | Q00711 | 81 | 2 | 2 | 3.4 | Carbohydrate metabolism; TCA | Saccharomyces cerevisiae S288c | Eukaryota; Fungi; Dikarya; Ascomycota; Saccharomycotina; Saccharomycetes; Saccharomycetales; Saccharomycetaceae; Saccharomyces. |
| Citrate synthase, mitochondrial | P00890 | 46 | 3 | 3 | 5.6 | Carbohydrate metabolism; TCA | Saccharomyces cerevisiae S288c | Eukaryota; Fungi; Dikarya; Ascomycota; Saccharomycotina; Saccharomycetes; Saccharomycetales; Saccharomycetaceae; Saccharomyces. |
| Aconitate hydratase, mitochondrial | P19414 | 46 | 1 | 1 | 1.5 | Carbohydrate metabolism; TCA | Saccharomyces cerevisiae S288c | Eukaryota; Fungi; Dikarya; Ascomycota; Saccharomycotina; Saccharomycetes; Saccharomycetales; Saccharomycetaceae; Saccharomyces. |
| Golgin IMH1 | Q06704 | 31 | 4 | 4 | 3.7 | Protein transport | Saccharomyces cerevisiae S288c | Eukaryota; Fungi; Dikarya; Ascomycota; Saccharomycotina; Saccharomycetes; Saccharomycetales; Saccharomycetaceae; Saccharomyces. |
| Uncharacterized protein YKR023W | P36119 | 28 | 7 | 1 | 1.1 | Unknown | Saccharomyces cerevisiae S288c | Eukaryota; Fungi; Dikarya; Ascomycota; Saccharomycotina; Saccharomycetes; Saccharomycetales; Saccharomycetaceae; Saccharomyces. |
| Endo-1,3(4)-beta-glucanase 1 | P53753 | 27 | 1 | 1 | 0.9 | Carbohydrate metabolism; cell wall degradation | Saccharomyces cerevisiae S288c | Eukaryota; Fungi; Dikarya; Ascomycota; Saccharomycotina; Saccharomycetes; Saccharomycetales; Saccharomycetaceae; Saccharomyces. |
| Inositol-3-phosphate synthase | P11986 | 26 | 1 | 1 | 2.1 | Lipid metabolism | Saccharomyces cerevisiae S288c | Eukaryota; Fungi; Dikarya; Ascomycota; Saccharomycotina; Saccharomycetes; Saccharomycetales; Saccharomycetaceae; Saccharomyces. |
| mRNA transport factor GFD1 | Q04839 | 25 | 1 | 1 | 3.7 | Translation | Saccharomyces cerevisiae S288c | Eukaryota; Fungi; Dikarya; Ascomycota; Saccharomycotina; Saccharomycetes; Saccharomycetales; Saccharomycetaceae; Saccharomyces. |
| Uncharacterized protein YHL026C | P38740 | 25 | 6 | 1 | 4.1 | Unknown | Saccharomyces cerevisiae S288c | Eukaryota; Fungi; Dikarya; Ascomycota; Saccharomycotina; Saccharomycetes; Saccharomycetales; Saccharomycetaceae; Saccharomyces. |
| Vacuolar protein sorting-associated protein 45 | P38932 | 24 | 1 | 1 | 1.7 | Transport | Saccharomyces cerevisiae S288c | Eukaryota; Fungi; Dikarya; Ascomycota; Saccharomycotina; Saccharomycetes; Saccharomycetales; Saccharomycetaceae; Saccharomyces. |
| Uncharacterized GTP-binding protein YDR336W | Q05473 | 24 | 5 | 1 | 1 | Unknown | Saccharomyces cerevisiae S288c | Eukaryota; Fungi; Dikarya; Ascomycota; Saccharomycotina; Saccharomycetes; Saccharomycetales; Saccharomycetaceae; Saccharomyces. |
| U1 SNP1-associating protein 1 | Q03714 | 21 | 12 | 2 | 1.7 | Transcription | Saccharomyces cerevisiae S288c | Eukaryota; Fungi; Dikarya; Ascomycota; Saccharomycotina; Saccharomycetes; Saccharomycetales; Saccharomycetaceae; Saccharomyces. |
| Regulator of Ty1 transposition protein 101 | P47050 | 13 | 2 | 2 | 1.8 | Other | Saccharomyces cerevisiae S288c | Eukaryota; Fungi; Dikarya; Ascomycota; Saccharomycotina; Saccharomycetes; Saccharomycetales; Saccharomycetaceae; Saccharomyces. |
| Ubiquitin carboxyl-terminal hydrolase 4 | B3LGK1 | 35 | 2 | 1 | 0.8 | Other | Saccharomyces cerevisiae RM11-1a | Eukaryota; Fungi; Dikarya; Ascomycota; Saccharomycotina; Saccharomycetes; Saccharomycetales; Saccharomycetaceae; Saccharomyces. |
| ATP-dependent RNA helicase DBP10 | A6ZXU0 | 19 | 7 | 4 | 3 | Ribosome biogenesis; rRNA processing | Saccharomyces cerevisiae YJM789 | Eukaryota; Fungi; Dikarya; Ascomycota; Saccharomycotina; Saccharomycetes; Saccharomycetales; Saccharomycetaceae; Saccharomyces. |
| Glyceraldehyde-3-phosphate dehydrogenase 2 | Q6FSM4 | 25 | 4 | 3 | 10.2 | Carbohydrate metabolism; Glycolysis | Candida glabrata CBS 138 | Eukaryota; Fungi; Dikarya; Ascomycota; Saccharomycotina; Saccharomycetes; Saccharomycetales; Saccharomycetaceae; Nakaseomyces; mitosporic Nakaseomyces. |
| Cell division control protein 10 | P39827 | 44 | 1 | 1 | 1.7 | Cell growth and death | Candida albicans SC5314 | Eukaryota; Fungi; Dikarya; Ascomycota; Saccharomycotina; Saccharomycetes; Saccharomycetales; mitosporic Saccharomycetales; Candida. |
| Malate dehydrogenase, cytoplasmic | P83778 | 38 | 1 | 1 | 3.3 | Carbohydrate metabolism; TCA | Candida albicans SC5314 | Eukaryota; Fungi; Dikarya; Ascomycota; Saccharomycotina; Saccharomycetes; Saccharomycetales; mitosporic Saccharomycetales; Candida. |
| Heat shock protein SSC1, mitochondrial | P83784 | 36 | 2 | 2 | 3.7 | Chaperone | Candida albicans SC5314 | Eukaryota; Fungi; Dikarya; Ascomycota; Saccharomycotina; Saccharomycetes; Saccharomycetales; mitosporic Saccharomycetales; Candida. |
| Helicase SWR1 | Q59U81 | 28 | 2 | 1 | 0.5 | Transcription | Candida albicans SC5314 | Eukaryota; Fungi; Dikarya; Ascomycota; Saccharomycotina; Saccharomycetes; Saccharomycetales; mitosporic Saccharomycetales; Candida. |
| Elongation factor 1-alpha 1 | P0CY35 | 25 | 1 | 1 | 1.7 | Protein biosynthesis | Candida albicans SC5314 | Eukaryota; Fungi; Dikarya; Ascomycota; Saccharomycotina; Saccharomycetes; Saccharomycetales; mitosporic Saccharomycetales; Candida. |
| Eukaryotic translation initiation factor 3 subunit C | Q5AML1 | 23 | 4 | 1 | 1.4 | Protein biosynthesis | Candida albicans SC5314 | Eukaryota; Fungi; Dikarya; Ascomycota; Saccharomycotina; Saccharomycetes; Saccharomycetales; mitosporic Saccharomycetales; Candida. |
| Heat shock protein SSB1 | P87222 | 19 | 2 | 2 | 4.4 | Chaperone | Candida albicans WO-1 | Eukaryota; Fungi; Dikarya; Ascomycota; Saccharomycotina; Saccharomycetes; Saccharomycetales; mitosporic Saccharomycetales; Candida. |
| ATPase synthesis protein 25, mitochondrial | C4XVT2 | 18 | 2 | 1 | 1.5 | Energy metabolism | Clavispora lusitaniae ATCC 42720 (anamorph: Candida lusitaniae ATCC 42720) | Eukaryota; Fungi; Dikarya; Ascomycota; Saccharomycotina; Saccharomycetes; Saccharomycetales; Metschnikowiaceae; Clavispora. |
| Mitochondrial inner membrane magnesium transporter MRS2 | Q6CLJ5 | 78 | 2 | 1 | 1.4 | Transport; Ion transport | Kluyveromyces lactis NRRL Y-1140 | Eukaryota; Fungi; Dikarya; Ascomycota; Saccharomycotina; Saccharomycetes; Saccharomycetales; Saccharomycetaceae; Kluyveromyces. |
| SVP1-like protein 2 | Q6CN23 | 32 | 2 | 2 | 5.6 | Transport; Protein transport | Kluyveromyces lactis NRRL Y-1140 | Eukaryota; Fungi; Dikarya; Ascomycota; Saccharomycotina; Saccharomycetes; Saccharomycetales; Saccharomycetaceae; Kluyveromyces. |
| Mediator of RNA polymerase II transcription subunit 15 | P32257 | 28 | 2 | 1 | 0.7 | Transcription | Kluyveromyces lactis NRRL Y-1140 | Eukaryota; Fungi; Dikarya; Ascomycota; Saccharomycotina; Saccharomycetes; Saccharomycetales; Saccharomycetaceae; Kluyveromyces. |
| COPII coat assembly protein SEC16 | Q6CM10 | 28 | 6 | 2 | 1.3 | Transport | Kluyveromyces lactis NRRL Y-1140 | Eukaryota; Fungi; Dikarya; Ascomycota; Saccharomycotina; Saccharomycetes; Saccharomycetales; Saccharomycetaceae; Kluyveromyces. |
| Actin-related protein 4 | Q6CSB9 | 24 | 1 | 1 | 1.3 | Cell motility | Kluyveromyces lactis NRRL Y-1140 | Eukaryota; Fungi; Dikarya; Ascomycota; Saccharomycotina; Saccharomycetes; Saccharomycetales; Saccharomycetaceae; Kluyveromyces. |
| Probable E3 ubiquitin-protein ligase TOM1 | Q756G2 | 48 | 22 | 5 | 1.2 | Protein modification; protein ubiquitination | Ashbya gossypii ATCC 10895 (Eremothecium gossypii ATCC 10895) | Eukaryota; Fungi; Dikarya; Ascomycota; Saccharomycotina; Saccharomycetes; Saccharomycetales; Saccharomycetaceae; Eremothecium. |
| Histone H2B.2 | Q8J1F8 | 37 | 6 | 3 | 20.5 | Replication and repair | Ashbya gossypii ATCC 10895 (Eremothecium gossypii ATCC 10895) | Eukaryota; Fungi; Dikarya; Ascomycota; Saccharomycotina; Saccharomycetes; Saccharomycetales; Saccharomycetaceae; Eremothecium. |
| Heat shock protein SSB | Q75E44 | 22 | 5 | 3 | 7.2 | Chaperone | Ashbya gossypii ATCC 10895 (Eremothecium gossypii ATCC 10895) | Eukaryota; Fungi; Dikarya; Ascomycota; Saccharomycotina; Saccharomycetes; Saccharomycetales; Saccharomycetaceae; Eremothecium. |
| Exonuclease V, mitochondrial | C5DXZ1 | 21 | 3 | 2 | 3.1 | Replication and repair | Zygosaccharomyces rouxii CBS 732 | Eukaryota; Fungi; Dikarya; Ascomycota; Saccharomycotina; Saccharomycetes; Saccharomycetales; Saccharomycetaceae; Zygosaccharomyces. |
| Vacuolar protein sorting/targeting protein 10 | B5RU30 | 44 | 4 | 2 | 0.8 | Transport; Protein transport | Debaryomyces hansenii CBS767 | Eukaryota; Fungi; Dikarya; Ascomycota; Saccharomycotina; Saccharomycetes; Saccharomycetales; Debaryomycetaceae; Debaryomyces. |
| mRNA-capping enzyme subunit alpha | Q6BT58 | 28 | 12 | 3 | 5.5 | Transcription | Debaryomyces hansenii CBS767 | Eukaryota; Fungi; Dikarya; Ascomycota; Saccharomycotina; Saccharomycetes; Saccharomycetales; Debaryomycetaceae; Debaryomyces. |
| mRNA cleavage and polyadenylation factor CLP1 | A5DY33 | 35 | 1 | 1 | 1.3 | Transcription | Lodderomyces elongisporus NRRL YB-4239 | Eukaryota; Fungi; Dikarya; Ascomycota; Saccharomycotina; Saccharomycetes; Saccharomycetales; Debaryomycetaceae; Lodderomyces. |
| Nucleolar protein 9 | A5DBF4 | 25 | 3 | 2 | 2.2 | Ribosome biogenesis; rRNA processing | Meyerozyma guilliermondii ATCC 6260 (Pichia guilliermondii ATCC 6260) | Eukaryota; Fungi; Dikarya; Ascomycota; Saccharomycotina; Saccharomycetes; Saccharomycetales; Debaryomycetaceae; Meyerozyma. |
| ATP-dependent RNA helicase DRS1 | A3LSN3 | 53 | 5 | 1 | 1.1 | Ribosome biogenesis | Scheffersomyces stipitis CBS 6054 (Pichia stipitis CBS 6054) | Eukaryota; Fungi; Dikarya; Ascomycota; Saccharomycotina; Saccharomycetes; Saccharomycetales; Debaryomycetaceae; Scheffersomyces. |
| Stress response protein NST1 | Q6CBW0 | 31 | 3 | 1 | 0.8 | Stress response; salt tolerance | Yarrowia lipolytica CLIB122 | Eukaryota; Fungi; Dikarya; Ascomycota; Saccharomycotina; Saccharomycetes; Saccharomycetales; Dipodascaceae; Yarrowia. |
| Pre-mRNA-splicing factor CLF1 | Q6C186 | 28 | 7 | 2 | 2.4 | Transcription | Yarrowia lipolytica CLIB122 | Eukaryota; Fungi; Dikarya; Ascomycota; Saccharomycotina; Saccharomycetes; Saccharomycetales; Dipodascaceae; Yarrowia. |
| Peptidyl-prolyl isomerase CWC27 | Q6CGK4 | 26 | 18 | 1 | 2.2 | Transcription | Yarrowia lipolytica CLIB122 | Eukaryota; Fungi; Dikarya; Ascomycota; Saccharomycotina; Saccharomycetes; Saccharomycetales; Dipodascaceae; Yarrowia. |
| Lipase 1 | P17573 | 98 | 1 | 1 | 2.5 | Lipid metabolism | Geotrichum candidum | Eukaryota; Fungi; Dikarya; Ascomycota; Saccharomycotina; Saccharomycetes; Saccharomycetales; Dipodascaceae; mitosporic Dipodascaceae; Geotrichum. |
| Proteasome component ecm29 | Q9P7H8 | 44 | 6 | 2 | 0.8 | Proteasome | Schizosaccharomyces pombe 972h- | Eukaryota; Fungi; Dikarya; Ascomycota; Taphrinomycotina; Schizosaccharomycetes; Schizosaccharomycetales; Schizosaccharomycetaceae; Schizosaccharomyces. |
| Heat shock protein 78, mitochondrial | O74402 | 32 | 4 | 3 | 3.2 | Chaperone | Schizosaccharomyces pombe 972h- | Eukaryota; Fungi; Dikarya; Ascomycota; Taphrinomycotina; Schizosaccharomycetes; Schizosaccharomycetales; Schizosaccharomycetaceae; Schizosaccharomyces. |
| Meiotic expression up-regulated protein 1/2 | Q9UTJ3 | 29 | 4 | 2 | 3.2 | Cell growth and death | Schizosaccharomyces pombe 972h- | Eukaryota; Fungi; Dikarya; Ascomycota; Taphrinomycotina; Schizosaccharomycetes; Schizosaccharomycetales; Schizosaccharomycetaceae; Schizosaccharomyces. |
| Metaxin-like protein C409.19c | Q9UUA5 | 27 | 6 | 2 | 3.1 | Transport; Protein transport | Schizosaccharomyces pombe 972h- | Eukaryota; Fungi; Dikarya; Ascomycota; Taphrinomycotina; Schizosaccharomycetes; Schizosaccharomycetales; Schizosaccharomycetaceae; Schizosaccharomyces. |
| Protein fyv4, mitochondrial | O74972 | 27 | 4 | 1 | 6.2 | Other | Schizosaccharomyces pombe 972h- | Eukaryota; Fungi; Dikarya; Ascomycota; Taphrinomycotina; Schizosaccharomycetes; Schizosaccharomycetales; Schizosaccharomycetaceae; Schizosaccharomyces. |
| Lid2 complex component lid2 | Q9HDV4 | 27 | 2 | 1 | 0.5 | Transcription | Schizosaccharomyces pombe 972h- | Eukaryota; Fungi; Dikarya; Ascomycota; Taphrinomycotina; Schizosaccharomycetes; Schizosaccharomycetales; Schizosaccharomycetaceae; Schizosaccharomyces. |
| DNA-directed RNA polymerase III subunit rpc1 | O94666 | 24 | 5 | 3 | 1.7 | Transcription | Schizosaccharomyces pombe 972h- | Eukaryota; Fungi; Dikarya; Ascomycota; Taphrinomycotina; Schizosaccharomycetes; Schizosaccharomycetales; Schizosaccharomycetaceae; Schizosaccharomyces. |
| Eukaryotic translation initiation factor 2-alpha kinase 1 | O13889 | 24 | 1 | 1 | 0 | Translation | Schizosaccharomyces pombe 972h- | Eukaryota; Fungi; Dikarya; Ascomycota; Taphrinomycotina; Schizosaccharomycetes; Schizosaccharomycetales; Schizosaccharomycetaceae; Schizosaccharomyces. |
| DNA replication licensing factor mcm10 | O42709 | 21 | 1 | 1 | 1.5 | Replication and repair | Schizosaccharomyces pombe 972h- | Eukaryota; Fungi; Dikarya; Ascomycota; Taphrinomycotina; Schizosaccharomycetes; Schizosaccharomycetales; Schizosaccharomycetaceae; Schizosaccharomyces. |
| Cohesin subunit psc3 | O13816 | 21 | 1 | 1 | 0.8 | Replication and repair | Schizosaccharomyces pombe 972h- | Eukaryota; Fungi; Dikarya; Ascomycota; Taphrinomycotina; Schizosaccharomycetes; Schizosaccharomycetales; Schizosaccharomycetaceae; Schizosaccharomyces. |
| 37S ribosomal protein S9, mitochondrial | Q7S7R6 | 37 | 2 | 2 | 4.8 | Ribosomes | Neurospora crassa OR74A | Eukaryota; Fungi; Dikarya; Ascomycota; Pezizomycotina; Sordariomycetes; Sordariomycetidae; Sordariales; Sordariaceae; Neurospora. |
| Deoxyhypusine synthase | P49365 | 35 | 1 | 1 | 1.7 | Protein modification | Neurospora crassa OR74A | Eukaryota; Fungi; Dikarya; Ascomycota; Pezizomycotina; Sordariomycetes; Sordariomycetidae; Sordariales; Sordariaceae; Neurospora. |
| Pyrroline-5-carboxylate reductase | Q12641 | 30 | 1 | 1 | 2.4 | Amino acid metabolism | Neurospora crassa OR74A | Eukaryota; Fungi; Dikarya; Ascomycota; Pezizomycotina; Sordariomycetes; Sordariomycetidae; Sordariales; Sordariaceae; Neurospora. |
| Exoglucanase 1 | P38676 | 23 | 3 | 1 | 2.3 | Carbohydrate metabolism; Cellulose metabolism | Neurospora crassa OR74A | Eukaryota; Fungi; Dikarya; Ascomycota; Pezizomycotina; Sordariomycetes; Sordariomycetidae; Sordariales; Sordariaceae; Neurospora. |
| Mannose-6-phosphate isomerase | Q870Y1 | 22 | 3 | 1 | 2.4 | Carbohydrate metabolism; Nucleotide-sugar biosynthesis | Neurospora crassa OR74A | Eukaryota; Fungi; Dikarya; Ascomycota; Pezizomycotina; Sordariomycetes; Sordariomycetidae; Sordariales; Sordariaceae; Neurospora. |
| Eukaryotic translation initiation factor 3 subunit C | Q2H731 | 46 | 5 | 1 | 0.9 | Translation | Chaetomium globosum CBS 148.51 | Eukaryota; Fungi; Dikarya; Ascomycota; Pezizomycotina; Sordariomycetes; Sordariomycetidae; Sordariales; Chaetomiaceae; Chaetomium. |
| Class E vacuolar protein-sorting machinery protein HSE1 | Q2GT05 | 26 | 1 | 1 | 1.8 | Transport; Protein transport | Chaetomium globosum CBS 148.51 | Eukaryota; Fungi; Dikarya; Ascomycota; Pezizomycotina; Sordariomycetes; Sordariomycetidae; Sordariales; Chaetomiaceae; Chaetomium. |
| Actin | Q6TCF2 | 186 | 11 | 6 | 18.7 | Cell motility | Gaeumannomyces graminis var. avenae | Eukaryota; Fungi; Dikarya; Ascomycota; Pezizomycotina; Sordariomycetes; Sordariomycetidae; Magnaporthales; Magnaporthaceae; Gaeumannomyces. |
| Glyceraldehyde-3-phosphate dehydrogenase | Q00584 | 50 | 1 | 1 | 4.2 | Carbohydrate metabolism; Glycolysis | Claviceps purpurea 20.1 | Eukaryota; Fungi; Dikarya; Ascomycota; Pezizomycotina; Sordariomycetes; Hypocreomycetidae; Hypocreales; Clavicipitaceae; Claviceps. |
| Putative exoglucanase type C | P46238 | 315 | 9 | 2 | 3.7 | Carbohydrate metabolism; Cellulose metabolism | Fusarium oxysporum | Eukaryota; Fungi; Dikarya; Ascomycota; Pezizomycotina; Sordariomycetes; Hypocreomycetidae; Hypocreales; Nectriaceae; mitosporic Nectriaceae; Fusarium; Fusarium oxysporum species complex. |
| Heat shock 70 kDa protein | Q5B2V1 | 260 | 13 | 7 | 10.9 | Chaperone | Aspergillus nidulans FGSC A4 | Eukaryota; Fungi; Dikarya; Ascomycota; Pezizomycotina; Eurotiomycetes; Eurotiomycetidae; Eurotiales; Aspergillaceae; Aspergillus. |
| Cell division control protein 48 | Q5AWS6 | 35 | 1 | 1 | 1 | Cell growth and death | Aspergillus nidulans FGSC A4 | Eukaryota; Fungi; Dikarya; Ascomycota; Pezizomycotina; Eurotiomycetes; Eurotiomycetidae; Eurotiales; Aspergillaceae; Aspergillus. |
| Catalase-peroxidase | A2Q7T1 | 19 | 1 | 1 | 2.5 | Oxidative stress | Aspergillus niger CBS 513.88 | Eukaryota; Fungi; Dikarya; Ascomycota; Pezizomycotina; Eurotiomycetes; Eurotiomycetidae; Eurotiales; Aspergillaceae; Aspergillus. |
| Catalase-peroxidase | Q2TW34 | 35 | 2 | 2 | 2.5 | Oxidative stress | Aspergillus oryzae RIB40 | Eukaryota; Fungi; Dikarya; Ascomycota; Pezizomycotina; Eurotiomycetes; Eurotiomycetidae; Eurotiales; Aspergillaceae; Aspergillus. |
| Dolichyl pyrophosphate Glc1Man9GlcNAc2 alpha-1,3-glucosyltransferase | Q2UB20 | 14 | 1 | 1 | 1.6 | Protein modification; protein glycosylation | Aspergillus oryzae RIB40 | Eukaryota; Fungi; Dikarya; Ascomycota; Pezizomycotina; Eurotiomycetes; Eurotiomycetidae; Eurotiales; Aspergillaceae; Aspergillus. |
| Mediator of RNA polymerase II transcription subunit 14 | Q0CL23 | 14 | 7 | 3 | 2.3 | Transcription | Aspergillus terreus NIH2624 | Eukaryota; Fungi; Dikarya; Ascomycota; Pezizomycotina; Eurotiomycetes; Eurotiomycetidae; Eurotiales; Aspergillaceae; Aspergillus. |
| Putative fatty acid synthase subunit TOXC | Q92215 | 19 | 6 | 1 | 0.4 | Lipid metabolism | Bipolaris zeicola | Eukaryota; Fungi; Dikarya; Ascomycota; Pezizomycotina; Dothideomycetes; Pleosporomycetidae; Pleosporales; Pleosporineae; Pleosporaceae; Bipolaris. |
| Protein GET1 | A8N7T9 | 28 | 4 | 2 | 7.9 | Transport | Coprinopsis cinerea okayama7#130 (Coprinus cinereus okayama7#130) | Eukaryota; Fungi; Dikarya; Basidiomycota; Agaricomycotina; Agaricomycetes; Agaricomycetidae; Agaricales; Psathyrellaceae; Coprinopsis. |
| Cellobiose dehydrogenase | Q01738 | 26 | 1 | 1 | 1 | Carbohydrate metabolism; Cellulose metabolism | Phanerochaete chrysosporium (anamorph: Sporotrichum pruinosum) | Eukaryota; Fungi; Dikarya; Basidiomycota; Agaricomycotina; Agaricomycetes; Corticiales; Corticiaceae; Phanerochaete. |
| Autophagy-related protein 18 | Q4P4N1 | 27 | 6 | 2 | 3.8 | Transport | Ustilago maydis 521 | Eukaryota; Fungi; Dikarya; Basidiomycota; Ustilaginomycotina; Ustilaginomycetes; Ustilaginales; Ustilaginaceae; Ustilago. |
| Transcription elongation factor SPT6 | Q4PI89 | 26 | 4 | 2 | 0.4 | Transcription | Ustilago maydis 521 | Eukaryota; Fungi; Dikarya; Basidiomycota; Ustilaginomycotina; Ustilaginomycetes; Ustilaginales; Ustilaginaceae; Ustilago. |
| Mitochondrial-type heat shock protein 70 | O96772 | 94 | 6 | 3 | 6.4 | Chaperone | Encephalitozoon cuniculi GB-M1 | Eukaryota; Fungi; Microsporidia; Unikaryonidae; Encephalitozoon. |
| Probable myosin heavy chain ECU04_1000 | Q8SS35 | 61 | 8 | 5 | 3.2 | Cell motility | Encephalitozoon cuniculi GB-M1 | Eukaryota; Fungi; Microsporidia; Unikaryonidae; Encephalitozoon. |
| Iron sulfur cluster assembly protein 1 | B0YLW7 | 19 | 5 | 1 | 5.1 | Other | Trachipleistophora hominis | Eukaryota; Fungi; Microsporidia; Pleistophoridae; Trachipleistophora. |
| Histone H4 | Q9HDF5 | 25 | 2 | 2 | 19.4 | Replication and repair | Mortierella alpina | Eukaryota; Fungi; Fungi incertae sedis; Early diverging fungal lineages; Mortierellomycotina; Mortierellales; Mortierellaceae; Mortierella. |
| **Samle LS 2** | | | | | | | | |
| Cell morphogenesis protein PAG1 | P40468 | 109 | 11 | 5 | 2 | Cell morphogenesis | Saccharomyces cerevisiae S288c | Eukaryota; Fungi; Dikarya; Ascomycota; Saccharomycotina; Saccharomycetes; Saccharomycetales; Saccharomycetaceae; Saccharomyces. |
| Low-affinity glucose transporter HXT1 | P32465 | 96 | 10 | 2 | 2.5 | Transport; Sugar transport | Saccharomyces cerevisiae S288c | Eukaryota; Fungi; Dikarya; Ascomycota; Saccharomycotina; Saccharomycetes; Saccharomycetales; Saccharomycetaceae; Saccharomyces. |
| Uncharacterized protein YDR179W-A | Q03983 | 59 | 5 | 2 | 4.8 | Other | Saccharomyces cerevisiae S288c | Eukaryota; Fungi; Dikarya; Ascomycota; Saccharomycotina; Saccharomycetes; Saccharomycetales; Saccharomycetaceae; Saccharomyces. |
| Coproporphyrinogen-III oxidase | P11353 | 51 | 2 | 2 | 5.8 | Metabolism of cofactors and vitamins | Saccharomyces cerevisiae S288c | Eukaryota; Fungi; Dikarya; Ascomycota; Saccharomycotina; Saccharomycetes; Saccharomycetales; Saccharomycetaceae; Saccharomyces. |
| Argininosuccinate lyase | P04076 | 43 | 1 | 1 | 1.5 | Amino-acid biosynthesis | Saccharomyces cerevisiae S288c | Eukaryota; Fungi; Dikarya; Ascomycota; Saccharomycotina; Saccharomycetes; Saccharomycetales; Saccharomycetaceae; Saccharomyces. |
| Eukaryotic translation initiation factor 5 | P38431 | 33 | 3 | 1 | 2 | Translation | Saccharomyces cerevisiae S288c | Eukaryota; Fungi; Dikarya; Ascomycota; Saccharomycotina; Saccharomycetes; Saccharomycetales; Saccharomycetaceae; Saccharomyces. |
| Uncharacterized protein YGR053C | P53234 | 31 | 1 | 1 | 2.8 | Other | Saccharomyces cerevisiae S288c | Eukaryota; Fungi; Dikarya; Ascomycota; Saccharomycotina; Saccharomycetes; Saccharomycetales; Saccharomycetaceae; Saccharomyces. |
| DDK kinase regulatory subunit DBF4 | P32325 | 27 | 3 | 2 | 2.4 | Replication and repair | Saccharomyces cerevisiae S288c | Eukaryota; Fungi; Dikarya; Ascomycota; Saccharomycotina; Saccharomycetes; Saccharomycetales; Saccharomycetaceae; Saccharomyces. |
| SWR1-complex protein 3 | P31376 | 27 | 1 | 1 | 1.3 | Replication and repair | Saccharomyces cerevisiae S288c | Eukaryota; Fungi; Dikarya; Ascomycota; Saccharomycotina; Saccharomycetes; Saccharomycetales; Saccharomycetaceae; Saccharomyces. |
| Serine/threonine-protein kinase GCN2 | P15442 | 25 | 7 | 4 | 2.1 | Translation | Saccharomyces cerevisiae S288c | Eukaryota; Fungi; Dikarya; Ascomycota; Saccharomycotina; Saccharomycetes; Saccharomycetales; Saccharomycetaceae; Saccharomyces. |
| Heat shock protein 104 | P31539 | 23 | 3 | 2 | 2 | Chaperone | Saccharomyces cerevisiae S288c | Eukaryota; Fungi; Dikarya; Ascomycota; Saccharomycotina; Saccharomycetes; Saccharomycetales; Saccharomycetaceae; Saccharomyces. |
| Putative uncharacterized protein YCL021W-A | Q96VH3 | 23 | 2 | 1 | 5 | Other | Saccharomyces cerevisiae S288c | Eukaryota; Fungi; Dikarya; Ascomycota; Saccharomycotina; Saccharomycetes; Saccharomycetales; Saccharomycetaceae; Saccharomyces. |
| ARF guanine-nucleotide exchange factor 1 | P47102 | 23 | 1 | 1 | 0.6 | Other | Saccharomyces cerevisiae S288c | Eukaryota; Fungi; Dikarya; Ascomycota; Saccharomycotina; Saccharomycetes; Saccharomycetales; Saccharomycetaceae; Saccharomyces. |
| Trans-acting factor D | Q06891 | 20 | 2 | 1 | 5 | Plasmid partition | Saccharomyces cerevisiae S288c | Eukaryota; Fungi; Dikarya; Ascomycota; Saccharomycotina; Saccharomycetes; Saccharomycetales; Saccharomycetaceae; Saccharomyces. |
| Inheritance of peroxisomes protein 2 | A6ZMM2 | 21 | 1 | 1 | 0.7 | Oxidative stress | Saccharomyces cerevisiae YJM789 | Eukaryota; Fungi; Dikarya; Ascomycota; Saccharomycotina; Saccharomycetes; Saccharomycetales; Saccharomycetaceae; Saccharomyces. |
| ATP-dependent RNA helicase DBP10 | A6ZXU0 | 20 | 8 | 3 | 2.4 | Ribosome biogenesis; rRNA processing | Saccharomyces cerevisiae YJM789 | Eukaryota; Fungi; Dikarya; Ascomycota; Saccharomycotina; Saccharomycetes; Saccharomycetales; Saccharomycetaceae; Saccharomyces. |
| Spindle pole component BBP1 | Q755J5 | 24 | 1 | 1 | 2.8 | Cell growth and death | Ashbya gossypii ATCC 10895 (Eremothecium gossypii ATCC 10895) | Eukaryota; Fungi; Dikarya; Ascomycota; Saccharomycotina; Saccharomycetes; Saccharomycetales; Saccharomycetaceae; Eremothecium. |
| Probable E3 ubiquitin-protein ligase TOM1 | Q756G2 | 121 | 17 | 2 | 0.7 | Protein modification; protein ubiquitination. | Ashbya gossypii ATCC 10895 (Eremothecium gossypii ATCC 10895) | Eukaryota; Fungi; Dikarya; Ascomycota; Saccharomycotina; Saccharomycetes; Saccharomycetales; Saccharomycetaceae; Eremothecium. |
| Serine/threonine-protein kinase MEC1 | Q75DB8 | 42 | 5 | 5 | 2.3 | Replication and repair | Ashbya gossypii ATCC 10895 (Eremothecium gossypii ATCC 10895) | Eukaryota; Fungi; Dikarya; Ascomycota; Saccharomycotina; Saccharomycetes; Saccharomycetales; Saccharomycetaceae; Eremothecium. |
| ATP-dependent RNA helicase CHL1 | Q750G3 | 38 | 2 | 1 | 1 | Replication and repair | Ashbya gossypii ATCC 10895 (Eremothecium gossypii ATCC 10895) | Eukaryota; Fungi; Dikarya; Ascomycota; Saccharomycotina; Saccharomycetes; Saccharomycetales; Saccharomycetaceae; Eremothecium. |
| Chromatin modification-related protein EAF1 | Q756L0 | 33 | 6 | 3 | 3 | Replication and repair | Ashbya gossypii ATCC 10895 (Eremothecium gossypii ATCC 10895) | Eukaryota; Fungi; Dikarya; Ascomycota; Saccharomycotina; Saccharomycetes; Saccharomycetales; Saccharomycetaceae; Eremothecium. |
| Kinesin-like protein KIP1 | Q8J1G4 | 28 | 5 | 2 | 2.1 | Cell motility | Ashbya gossypii ATCC 10895 (Eremothecium gossypii ATCC 10895) | Eukaryota; Fungi; Dikarya; Ascomycota; Saccharomycotina; Saccharomycetes; Saccharomycetales; Saccharomycetaceae; Eremothecium. |
| Vacuolar protein sorting-associated protein 27 | Q755J9 | 26 | 6 | 3 | 3.6 | Transport; Protein transport | Ashbya gossypii ATCC 10895 (Eremothecium gossypii ATCC 10895) | Eukaryota; Fungi; Dikarya; Ascomycota; Saccharomycotina; Saccharomycetes; Saccharomycetales; Saccharomycetaceae; Eremothecium. |
| ATP synthase subunit alpha, mitochondrial | P49375 | 36 | 3 | 3 | 6 | Energy metabolism | Kluyveromyces lactis NRRL Y-1140 | Eukaryota; Fungi; Dikarya; Ascomycota; Saccharomycotina; Saccharomycetes; Saccharomycetales; Saccharomycetaceae; Kluyveromyces. |
| 60S ribosomal subunit assembly/export protein LOC1 | Q6CN96 | 29 | 2 | 2 | 10.1 | Ribosome biogenesis | Kluyveromyces lactis NRRL Y-1140 | Eukaryota; Fungi; Dikarya; Ascomycota; Saccharomycotina; Saccharomycetes; Saccharomycetales; Saccharomycetaceae; Kluyveromyces. |
| Mitochondrial escape protein 2 | Q6CTB6 | 25 | 2 | 2 | 2.5 | Other | Kluyveromyces lactis NRRL Y-1140 | Eukaryota; Fungi; Dikarya; Ascomycota; Saccharomycotina; Saccharomycetes; Saccharomycetales; Saccharomycetaceae; Kluyveromyces. |
| Superoxide dismutase [Cu-Zn] | Q6CPE2 | 13 | 1 | 1 | 6.5 | Oxidative stress | Kluyveromyces lactis NRRL Y-1140 | Eukaryota; Fungi; Dikarya; Ascomycota; Saccharomycotina; Saccharomycetes; Saccharomycetales; Saccharomycetaceae; Kluyveromyces. |
| Myosin-3 | Q6FMJ3 | 39 | 6 | 3 | 1.7 | Cell motility | Candida glabrata CBS 138 | Eukaryota; Fungi; Dikarya; Ascomycota; Saccharomycotina; Saccharomycetes; Saccharomycetales; Saccharomycetaceae; Nakaseomyces; mitosporic Nakaseomyces. |
| Mitochondrial distribution and morphology protein 34 | Q6FQE0 | 23 | 4 | 3 | 6.3 | Other | Candida glabrata CBS 138 | Eukaryota; Fungi; Dikarya; Ascomycota; Saccharomycotina; Saccharomycetes; Saccharomycetales; Saccharomycetaceae; Nakaseomyces; mitosporic Nakaseomyces. |
| Autophagy protein 5 | A7TS83 | 23 | 1 | 1 | 2.4 | Transport; Protein transport | Vanderwaltozyma polyspora DSM 70294 | Eukaryota; Fungi; Dikarya; Ascomycota; Saccharomycotina; Saccharomycetes; Saccharomycetales; Saccharomycetaceae; Vanderwaltozyma. |
| 78 kDa glucose-regulated protein homolog | Q9HG01 | 36 | 1 | 1 | 1.8 | Stress response | Ogataea angusta | Eukaryota; Fungi; Dikarya; Ascomycota; Saccharomycotina; Saccharomycetes; Saccharomycetales; Saccharomycetales incertae sedis; Ogataea. |
| Peroxiredoxin TSA1 | Q9Y7F0 | 119 | 2 | 1 | 7.1 | Oxidative stress | Candida albicans SC5314 | Eukaryota; Fungi; Dikarya; Ascomycota; Saccharomycotina; Saccharomycetes; Saccharomycetales; mitosporic Saccharomycetales; Candida. |
| mRNA-capping enzyme subunit alpha | Q6BT58 | 24 | 3 | 1 | 2.2 | Transcription | Debaryomyces hansenii CBS767 | Eukaryota; Fungi; Dikarya; Ascomycota; Saccharomycotina; Saccharomycetes; Saccharomycetales; Debaryomycetaceae; Debaryomyces. |
| Nucleolar protein 16 | A5DYR5 | 34 | 1 | 1 | 3 | Ribosome biogenesis; rRNA processing. | Lodderomyces elongisporus NRRL YB-4239 | Eukaryota; Fungi; Dikarya; Ascomycota; Saccharomycotina; Saccharomycetes; Saccharomycetales; Debaryomycetaceae; Lodderomyces. |
| Elongation factor Tu, mitochondrial | A5DN78 | 18 | 2 | 2 | 5.6 | Protein biosynthesis | Meyerozyma guilliermondii ATCC 6260 (Pichia guilliermondii ATCC 6260) | Eukaryota; Fungi; Dikarya; Ascomycota; Saccharomycotina; Saccharomycetes; Saccharomycetales; Debaryomycetaceae; Meyerozyma. |
| DNA damage-inducible protein 1 | Q6CFI3 | 22 | 5 | 2 | 3.3 | Transport | Yarrowia lipolytica CLIB122 | Eukaryota; Fungi; Dikarya; Ascomycota; Saccharomycotina; Saccharomycetes; Saccharomycetales; Dipodascaceae; Yarrowia. |
| ATP-dependent RNA helicase dbp6 | A2QA23 | 29 | 3 | 2 | 3.4 | Ribosome biogenesis; rRNA processing | Aspergillus niger CBS 513.88 | Eukaryota; Fungi; Dikarya; Ascomycota; Pezizomycotina; Eurotiomycetes; Eurotiomycetidae; Eurotiales; Trichocomaceae; mitosporic Trichocomaceae; Aspergillus. |
| Amino-acid acetyltransferase, mitochondrial | Q0CY06 | 26 | 1 | 1 | 0.9 | Amino-acid biosynthesis | Aspergillus terreus NIH2624 | Eukaryota; Fungi; Dikarya; Ascomycota; Pezizomycotina; Eurotiomycetes; Eurotiomycetidae; Eurotiales; Trichocomaceae; mitosporic Trichocomaceae; Aspergillus. |
| Histone acetyltransferase ESA1 | Q2UMQ5 | 15 | 2 | 1 | 2.6 | Transcription | Aspergillus oryzae RIB40 | Eukaryota; Fungi; Dikarya; Ascomycota; Pezizomycotina; Eurotiomycetes; Eurotiomycetidae; Eurotiales; Trichocomaceae; mitosporic Trichocomaceae; Aspergillus. |
| DNA-directed RNA polymerase III subunit rpc3 | A6R9K6 | 25 | 2 | 2 | 2.9 | Transcription | Ajellomyces capsulatus NAm1 | Eukaryota; Fungi; Dikarya; Ascomycota; Pezizomycotina; Eurotiomycetes; Eurotiomycetidae; Onygenales; Ajellomycetaceae; Ajellomyces. |
| Dicer-like protein 1; ATP-dependent helicase DCL1 | Q1DKI1 | 53 | 6 | 5 | 3.8 | RNA interference | Coccidioides immitis RS | Eukaryota; Fungi; Dikarya; Ascomycota; Pezizomycotina; Eurotiomycetes; Eurotiomycetidae; Onygenales; mitosporic Onygenales; Coccidioides. |
| ATP synthase subunit beta, mitochondrial | P23704 | 70 | 2 | 2 | 4 | Energy metabolism | Neurospora crassa OR74A | Eukaryota; Fungi; Dikarya; Ascomycota; Pezizomycotina; Sordariomycetes; Sordariomycetidae; Sordariales; Sordariaceae; Neurospora. |
| Dynein heavy chain, cytoplasmic | P45443 | 25 | 13 | 10 | 2.3 | Cell motility | Neurospora crassa OR74A | Eukaryota; Fungi; Dikarya; Ascomycota; Pezizomycotina; Sordariomycetes; Sordariomycetidae; Sordariales; Sordariaceae; Neurospora. |
| ATP-dependent RNA helicase MRH4, mitochondrial | Q2H679 | 32 | 6 | 4 | 6.1 | mitochondrial RNA metabolism | Chaetomium globosum CBS 148.51 | Eukaryota; Fungi; Dikarya; Ascomycota; Pezizomycotina; Sordariomycetes; Sordariomycetidae; Sordariales; Chaetomiaceae; Chaetomium. |
| Glyceraldehyde-3-phosphate dehydrogenase | Q00584 | 67 | 11 | 2 | 6.2 | Carbohydrate metabolism | Claviceps purpurea (ergot fungus) | Eukaryota; Fungi; Dikarya; Ascomycota; Pezizomycotina; Sordariomycetes; Hypocreomycetidae; Hypocreales; Clavicipitaceae; Claviceps. |
| Ubiquitin-40S ribosomal protein S27a | P0C016 | 34 | 3 | 3 | 22 | ribosome biogenesis | Schizosaccharomyces pombe 972h- | Eukaryota; Fungi; Dikarya; Ascomycota; Taphrinomycotina; Schizosaccharomycetes; Schizosaccharomycetales; Schizosaccharomycetaceae; Schizosaccharomyces. |
| Pre-mRNA-splicing factor cwf10 | O94316 | 31 | 3 | 2 | 1.7 | Transcription; mRNA processing | Schizosaccharomyces pombe 972h- | Eukaryota; Fungi; Dikarya; Ascomycota; Taphrinomycotina; Schizosaccharomycetes; Schizosaccharomycetales; Schizosaccharomycetaceae; Schizosaccharomyces. |
| Lid2 complex component lid2 | Q9HDV4 | 27 | 5 | 4 | 2.5 | Transcription | Schizosaccharomyces pombe 972h- | Eukaryota; Fungi; Dikarya; Ascomycota; Taphrinomycotina; Schizosaccharomycetes; Schizosaccharomycetales; Schizosaccharomycetaceae; Schizosaccharomyces. |
| Valine--tRNA ligase, mitochondrial | O14160 | 24 | 3 | 3 | 3.2 | Protein biosynthesis | Schizosaccharomyces pombe 972h- | Eukaryota; Fungi; Dikarya; Ascomycota; Taphrinomycotina; Schizosaccharomycetes; Schizosaccharomycetales; Schizosaccharomycetaceae; Schizosaccharomyces. |
| Cation-transporting ATPase 4 | O14072 | 22 | 1 | 1 | 0.6 | Transpot | Schizosaccharomyces pombe 972h- | Eukaryota; Fungi; Dikarya; Ascomycota; Taphrinomycotina; Schizosaccharomycetes; Schizosaccharomycetales; Schizosaccharomycetaceae; Schizosaccharomyces. |
| Brix domain-containing protein C1B9.03c | O14206 | 22 | 1 | 1 | 1.8 | ribosomal large subunit assembly | Schizosaccharomyces pombe 972h- | Eukaryota; Fungi; Dikarya; Ascomycota; Taphrinomycotina; Schizosaccharomycetes; Schizosaccharomycetales; Schizosaccharomycetaceae; Schizosaccharomyces. |
| Autophagy-related protein 18 | Q4P4N1 | 26 | 4 | 2 | 3.8 | Transport; Protein transport | Ustilago maydis 521 | Eukaryota; Fungi; Dikarya; Basidiomycota; Ustilaginomycotina; Ustilaginomycetes; Ustilaginales; Ustilaginaceae; Ustilago. |
| Transcription elongation factor SPT6 | Q4PI89 | 23 | 3 | 3 | 1.3 | Transcription | Ustilago maydis 521 | Eukaryota; Fungi; Dikarya; Basidiomycota; Ustilaginomycotina; Ustilaginomycetes; Ustilaginales; Ustilaginaceae; Ustilago. |
| Actin | P53689 | 323 | 11 | 4 | 12 | Cell motility | Xanthophyllomyces dendrorhous | Eukaryota; Fungi; Dikarya; Basidiomycota; Agaricomycotina; Tremellomycetes; Cystofilobasidiales; Cystofilobasidiaceae; Xanthophyllomyces. |
| Probable myosin heavy chain ECU04_1000 | Q8SS35 | 97 | 5 | 2 | 1.4 | Cell motility | Encephalitozoon cuniculi GB-M1 | Eukaryota; Fungi; Microsporidia; Unikaryonidae; Encephalitozoon. |
| UPF0329 protein ECU01_0110/ECU01_1500/ECU08_0040 | Q8STB5 | 18 | 2 | 2 | 2.5 | Other | Encephalitozoon cuniculi GB-M1 | Eukaryota; Fungi; Microsporidia; Unikaryonidae; Encephalitozoon. |
| Histone H4 | Q9HDF5 | 58 | 2 | 2 | 17.5 | Replication and repair | Mortierella alpina | Eukaryota; Fungi; Fungi incertae sedis; Early diverging fungal lineages; Mortierellomycotina; Mortierellales; Mortierellaceae; Mortierella. |
| **Samle LS 3** | | | | | | | | |
| Cell morphogenesis protein PAG1 | P40468 | 100 | 10 | 5 | 2.1 | Cell morphogenesis | Saccharomyces cerevisiae S288c | Eukaryota; Fungi; Dikarya; Ascomycota; Saccharomycotina; Saccharomycetes; Saccharomycetales; Saccharomycetaceae; Saccharomyces. |
| ATP synthase subunit alpha, mitochondrial | P07251 | 47 | 1 | 1 | 2.4 | Energy metabolism | Saccharomyces cerevisiae S288c | Eukaryota; Fungi; Dikarya; Ascomycota; Saccharomycotina; Saccharomycetes; Saccharomycetales; Saccharomycetaceae; Saccharomyces. |
| Coproporphyrinogen-III oxidase | P11353 | 43 | 2 | 2 | 5.8 | Metabolism of cofactors and vitamins | Saccharomyces cerevisiae S288c | Eukaryota; Fungi; Dikarya; Ascomycota; Saccharomycotina; Saccharomycetes; Saccharomycetales; Saccharomycetaceae; Saccharomyces. |
| Argininosuccinate lyase | P04076 | 43 | 2 | 2 | 3.7 | Amino-acid metabolism | Saccharomyces cerevisiae S288c | Eukaryota; Fungi; Dikarya; Ascomycota; Saccharomycotina; Saccharomycetes; Saccharomycetales; Saccharomycetaceae; Saccharomyces. |
| Uncharacterized protein YDR179W-A | Q03983 | 41 | 4 | 2 | 4.8 | Other | Saccharomyces cerevisiae S288c | Eukaryota; Fungi; Dikarya; Ascomycota; Saccharomycotina; Saccharomycetes; Saccharomycetales; Saccharomycetaceae; Saccharomyces. |
| Uncharacterized protein YBR063C | P38083 | 40 | 1 | 1 | 1.5 | Other | Saccharomyces cerevisiae S288c | Eukaryota; Fungi; Dikarya; Ascomycota; Saccharomycotina; Saccharomycetes; Saccharomycetales; Saccharomycetaceae; Saccharomyces. |
| DDK kinase regulatory subunit DBF4 | P32325 | 32 | 4 | 2 | 2.1 | Replication and repair | Saccharomyces cerevisiae S288c | Eukaryota; Fungi; Dikarya; Ascomycota; Saccharomycotina; Saccharomycetes; Saccharomycetales; Saccharomycetaceae; Saccharomyces. |
| Lysine--tRNA ligase, cytoplasmic | P15180 | 30 | 3 | 1 | 1.4 | Protein biosynthesis | Saccharomyces cerevisiae S288c | Eukaryota; Fungi; Dikarya; Ascomycota; Saccharomycotina; Saccharomycetes; Saccharomycetales; Saccharomycetaceae; Saccharomyces. |
| Aminodeoxychorismate lyase | Q03266 | 27 | 4 | 2 | 4.3 | Metabolism of cofactors and vitamins | Saccharomyces cerevisiae S288c | Eukaryota; Fungi; Dikarya; Ascomycota; Saccharomycotina; Saccharomycetes; Saccharomycetales; Saccharomycetaceae; Saccharomyces. |
| ARF guanine-nucleotide exchange factor 1 | P47102 | 26 | 3 | 3 | 2 | Other | Saccharomyces cerevisiae S288c | Eukaryota; Fungi; Dikarya; Ascomycota; Saccharomycotina; Saccharomycetes; Saccharomycetales; Saccharomycetaceae; Saccharomyces. |
| Putative transcription factor PHD1 | P36093 | 26 | 1 | 1 | 1.6 | Transcription | Saccharomyces cerevisiae S288c | Eukaryota; Fungi; Dikarya; Ascomycota; Saccharomycotina; Saccharomycetes; Saccharomycetales; Saccharomycetaceae; Saccharomyces. |
| Protein SPT3 | P06844 | 26 | 1 | 1 | 2.1 | Transcription | Saccharomyces cerevisiae S288c | Eukaryota; Fungi; Dikarya; Ascomycota; Saccharomycotina; Saccharomycetes; Saccharomycetales; Saccharomycetaceae; Saccharomyces. |
| tRNA-specific adenosine deaminase subunit TAD3 | Q9URQ3 | 26 | 1 | 1 | 2.5 | Translation; tRNA processing | Saccharomyces cerevisiae S288c | Eukaryota; Fungi; Dikarya; Ascomycota; Saccharomycotina; Saccharomycetes; Saccharomycetales; Saccharomycetaceae; Saccharomyces. |
| Protein PSP1 | P50896 | 25 | 3 | 2 | 2.7 | Replication and repair | Saccharomyces cerevisiae S288c | Eukaryota; Fungi; Dikarya; Ascomycota; Saccharomycotina; Saccharomycetes; Saccharomycetales; Saccharomycetaceae; Saccharomyces. |
| Uncharacterized protein YJR098C | P47139 | 24 | 1 | 1 | 1.2 | Other | Saccharomyces cerevisiae S288c | Eukaryota; Fungi; Dikarya; Ascomycota; Saccharomycotina; Saccharomycetes; Saccharomycetales; Saccharomycetaceae; Saccharomyces. |
| Mitochondrial chaperone BCS1 | P32839 | 22 | 13 | 1 | 2.2 | Chaperone | Saccharomyces cerevisiae S288c | Eukaryota; Fungi; Dikarya; Ascomycota; Saccharomycotina; Saccharomycetes; Saccharomycetales; Saccharomycetaceae; Saccharomyces. |
| Threonine--tRNA ligase, cytoplasmic | P04801 | 21 | 7 | 5 | 5.2 | Protein biosynthesis | Saccharomyces cerevisiae S288c | Eukaryota; Fungi; Dikarya; Ascomycota; Saccharomycotina; Saccharomycetes; Saccharomycetales; Saccharomycetaceae; Saccharomyces. |
| Probable ATP-dependent RNA helicase DHR1 | Q04217 | 19 | 8 | 6 | 4.7 | Ribosome biogenesis; rRNA processing | Saccharomyces cerevisiae S288c | Eukaryota; Fungi; Dikarya; Ascomycota; Saccharomycotina; Saccharomycetes; Saccharomycetales; Saccharomycetaceae; Saccharomyces. |
| MAP-homologous protein 1 | P43638 | 16 | 5 | 4 | 2.5 | Cell motility; Microtubule | Saccharomyces cerevisiae S288c | Eukaryota; Fungi; Dikarya; Ascomycota; Saccharomycotina; Saccharomycetes; Saccharomycetales; Saccharomycetaceae; Saccharomyces. |
| Low-affinity glucose transporter HXT4 | A6ZT02 | 68 | 9 | 2 | 3.3 | Transport; Sugar transport | Saccharomyces cerevisiae YJM789 | Eukaryota; Fungi; Dikarya; Ascomycota; Saccharomycotina; Saccharomycetes; Saccharomycetales; Saccharomycetaceae; Saccharomyces. |
| Myosin-3 | Q6FMJ3 | 41 | 6 | 4 | 2.4 | Cell motility | Candida glabrata CBS 138 | Eukaryota; Fungi; Dikarya; Ascomycota; Saccharomycotina; Saccharomycetes; Saccharomycetales; Saccharomycetaceae; Nakaseomyces; mitosporic Nakaseomyces. |
| Peroxiredoxin TSA1 | Q9Y7F0 | 71 | 2 | 1 | 7.1 | Oxidative stress | Candida albicans SC5314 | Eukaryota; Fungi; Dikarya; Ascomycota; Saccharomycotina; Saccharomycetes; Saccharomycetales; mitosporic Saccharomycetales; Candida. |
| rRNA-processing protein EFG1 | Q5AK42 | 22 | 6 | 4 | 14.8 | Ribosome biogenesis; rRNA processing | Candida albicans SC5314 | Eukaryota; Fungi; Dikarya; Ascomycota; Saccharomycotina; Saccharomycetes; Saccharomycetales; mitosporic Saccharomycetales; Candida. |
| Probable E3 ubiquitin-protein ligase TOM1 | Q756G2 | 59 | 12 | 2 | 0.6 | Protein modification; protein ubiquitination | Ashbya gossypii ATCC 10895 (Eremothecium gossypii ATCC 10895) | Eukaryota; Fungi; Dikarya; Ascomycota; Saccharomycotina; Saccharomycetes; Saccharomycetales; Saccharomycetaceae; Eremothecium. |
| Serine/threonine-protein kinase MEC1 | Q75DB8 | 41 | 8 | 5 | 2 | Replication and repair | Ashbya gossypii ATCC 10895 (Eremothecium gossypii ATCC 10895) | Eukaryota; Fungi; Dikarya; Ascomycota; Saccharomycotina; Saccharomycetes; Saccharomycetales; Saccharomycetaceae; Eremothecium. |
| ATP-dependent RNA helicase CHL1 | Q750G3 | 40 | 2 | 2 | 3 | Replication and repair | Ashbya gossypii ATCC 10895 (Eremothecium gossypii ATCC 10895) | Eukaryota; Fungi; Dikarya; Ascomycota; Saccharomycotina; Saccharomycetes; Saccharomycetales; Saccharomycetaceae; Eremothecium. |
| Chromatin modification-related protein EAF1 | Q756L0 | 35 | 8 | 4 | 4 | Replication and repair | Ashbya gossypii ATCC 10895 (Eremothecium gossypii ATCC 10895) | Eukaryota; Fungi; Dikarya; Ascomycota; Saccharomycotina; Saccharomycetes; Saccharomycetales; Saccharomycetaceae; Eremothecium. |
| Lon protease homolog 2, peroxisomal | Q6CWS4 | 20 | 3 | 2 | 2.6 | Protease | Kluyveromyces lactis NRRL Y-1140 | Eukaryota; Fungi; Dikarya; Ascomycota; Saccharomycotina; Saccharomycetes; Saccharomycetales; Saccharomycetaceae; Kluyveromyces. |
| Pentafunctional AROM polypeptide | C4R4R8 | 26 | 2 | 1 | 0.6 | Amino acid metabolism | Komagataella pastoris GS115 (Pichia pastoris GS115) | Eukaryota; Fungi; Dikarya; Ascomycota; Saccharomycotina; Saccharomycetes; Saccharomycetales; Saccharomycetaceae; Komagataella. |
| High osmolarity signaling protein SHO1 | C5DE38 | 31 | 1 | 1 | 1.5 | Stress response | Lachancea thermotolerans CBS 6340 (Kluyveromyces thermotolerans CBS 6340) | Eukaryota; Fungi; Dikarya; Ascomycota; Saccharomycotina; Saccharomycetes; Saccharomycetales; Saccharomycetaceae; Lachancea. |
| Autophagy-related protein 21 | Q5QJC0 | 29 | 1 | 1 | 2.1 | Transport; Protein transport | Ogataea angusta | Eukaryota; Fungi; Dikarya; Ascomycota; Saccharomycotina; Saccharomycetes; Saccharomycetales; Saccharomycetales incertae sedis; Ogataea. |
| Protein DSE1 | A3LTS5 | 36 | 4 | 2 | 2.3 | Outer membrane proteins; Cell wall biogenesis/degradation | Scheffersomyces stipitis CBS 6054 (Pichia stipitis CBS 6054) | Eukaryota; Fungi; Dikarya; Ascomycota; Saccharomycotina; Saccharomycetes; Saccharomycetales; Debaryomycetaceae; Scheffersomyces. |
| Actin cytoskeleton-regulatory complex protein END3 | A5DF78 | 19 | 4 | 3 | 8.2 | Cell motility | Meyerozyma guilliermondii ATCC 6260 (Pichia guilliermondii ATCC 6260) | Eukaryota; Fungi; Dikarya; Ascomycota; Saccharomycotina; Saccharomycetes; Saccharomycetales; Debaryomycetaceae; Meyerozyma. |
| Pentafunctional AROM polypeptide 1 | A5H2Q8 | 16 | 7 | 5 | 2.8 | Amino acid metabolism | Lodderomyces elongisporus NRRL YB-4239 | Eukaryota; Fungi; Dikarya; Ascomycota; Saccharomycotina; Saccharomycetes; Saccharomycetales; Debaryomycetaceae; Lodderomyces. |
| Autophagy-related protein 2 | Q6C6M0 | 30 | 4 | 2 | 1.5 | Transport; Protein transport | Yarrowia lipolytica CLIB122 | Eukaryota; Fungi; Dikarya; Ascomycota; Saccharomycotina; Saccharomycetes; Saccharomycetales; Dipodascaceae; Yarrowia. |
| ATP-dependent rRNA helicase SPB4 | Q6C193 | 23 | 4 | 2 | 3.7 | Ribosome biogenesis; rRNA processing | Yarrowia lipolytica CLIB122 | Eukaryota; Fungi; Dikarya; Ascomycota; Saccharomycotina; Saccharomycetes; Saccharomycetales; Dipodascaceae; Yarrowia. |
| Pre-mRNA-splicing factor cwf10 | O94316 | 33 | 2 | 2 | 1.7 | Transcription; mRNA processing | Schizosaccharomyces pombe 972h- | Eukaryota; Fungi; Dikarya; Ascomycota; Taphrinomycotina; Schizosaccharomycetes; Schizosaccharomycetales; Schizosaccharomycetaceae; Schizosaccharomyces. |
| Lid2 complex component lid2 | Q9HDV4 | 29 | 6 | 5 | 3.2 | Transcription | Schizosaccharomyces pombe 972h- | Eukaryota; Fungi; Dikarya; Ascomycota; Taphrinomycotina; Schizosaccharomycetes; Schizosaccharomycetales; Schizosaccharomycetaceae; Schizosaccharomyces. |
| Spindle assembly checkpoint component mad1 | P87169 | 25 | 4 | 2 | 2.6 | Cell growth and death; Mitosis | Schizosaccharomyces pombe 972h- | Eukaryota; Fungi; Dikarya; Ascomycota; Taphrinomycotina; Schizosaccharomycetes; Schizosaccharomycetales; Schizosaccharomycetaceae; Schizosaccharomyces. |
| Uncharacterized ABC transporter ATP-binding protein C825.01 | Q9USH9 | 25 | 3 | 2 | 1.3 | Other | Schizosaccharomyces pombe 972h- | Eukaryota; Fungi; Dikarya; Ascomycota; Taphrinomycotina; Schizosaccharomycetes; Schizosaccharomycetales; Schizosaccharomycetaceae; Schizosaccharomyces. |
| Hexokinase-2 | P50521 | 24 | 1 | 1 | 1.5 | Carbohydrate metabolism; Glycolysis | Schizosaccharomyces pombe 972h- | Eukaryota; Fungi; Dikarya; Ascomycota; Taphrinomycotina; Schizosaccharomycetes; Schizosaccharomycetales; Schizosaccharomycetaceae; Schizosaccharomyces. |
| Elongation factor Tu, mitochondrial | Q9Y700 | 22 | 2 | 2 | 5.2 | Protein biosynthesis | Schizosaccharomyces pombe 972h- | Eukaryota; Fungi; Dikarya; Ascomycota; Taphrinomycotina; Schizosaccharomycetes; Schizosaccharomycetales; Schizosaccharomycetaceae; Schizosaccharomyces. |
| Probable inositol polyphosphate 5-phosphatase C9G1.10c | O14306 | 18 | 4 | 4 | 4 | Other | Schizosaccharomyces pombe 972h- | Eukaryota; Fungi; Dikarya; Ascomycota; Taphrinomycotina; Schizosaccharomycetes; Schizosaccharomycetales; Schizosaccharomycetaceae; Schizosaccharomyces. |
| LisH domain-containing protein C29A3.03c | O59668 | 18 | 3 | 1 | 2.3 | Other | Schizosaccharomyces pombe 972h- | Eukaryota; Fungi; Dikarya; Ascomycota; Taphrinomycotina; Schizosaccharomycetes; Schizosaccharomycetales; Schizosaccharomycetaceae; Schizosaccharomyces. |
| Phosphatidylinositol 3-kinase tor1 | O14356 | 17 | 1 | 1 | 0.3 | Stress response | Schizosaccharomyces pombe 972h- | Eukaryota; Fungi; Dikarya; Ascomycota; Taphrinomycotina; Schizosaccharomycetes; Schizosaccharomycetales; Schizosaccharomycetaceae; Schizosaccharomyces. |
| tRNA-dihydrouridine(47) synthase [NAD(P)(+)] | A2QAU6 | 31 | 2 | 2 | 2 | Protein biosynthesis; tRNA processing | Aspergillus niger CBS 513.88 | Eukaryota; Fungi; Dikarya; Ascomycota; Pezizomycotina; Eurotiomycetes; Eurotiomycetidae; Eurotiales; Aspergillaceae; Aspergillus. |
| Probable beta-glucosidase I | A2R989 | 20 | 2 | 2 | 1.3 | Carbohydrate metabolism; Cellulose degradation | Aspergillus niger CBS 513.88 | Eukaryota; Fungi; Dikarya; Ascomycota; Pezizomycotina; Eurotiomycetes; Eurotiomycetidae; Eurotiales; Aspergillaceae; Aspergillus. |
| ATP-dependent RNA helicase dbp6 | A2QA23 | 20 | 2 | 2 | 3 | Ribosome biogenesis; rRNA processing | Aspergillus niger CBS 513.88 | Eukaryota; Fungi; Dikarya; Ascomycota; Pezizomycotina; Eurotiomycetes; Eurotiomycetidae; Eurotiales; Aspergillaceae; Aspergillus. |
| Triosephosphate isomerase | Q9HGY8 | 19 | 2 | 2 | 10 | Carbohydrate metabolism; glycolysis | Aspergillus oryzae RIB40 | Eukaryota; Fungi; Dikarya; Ascomycota; Pezizomycotina; Eurotiomycetes; Eurotiomycetidae; Eurotiales; Aspergillaceae; Aspergillus. |
| ATP synthase subunit beta, mitochondrial | P23704 | 39 | 3 | 2 | 4 | Energy metabolism | Neurospora crassa OR74A | Eukaryota; Fungi; Dikarya; Ascomycota; Pezizomycotina; Sordariomycetes; Sordariomycetidae; Sordariales; Sordariaceae; Neurospora. |
| Glyceraldehyde-3-phosphate dehydrogenase | Q00584 | 45 | 9 | 2 | 6.2 | Carbohydrate metabolism; Glycolysis | Claviceps purpurea 20.1 | Eukaryota; Fungi; Dikarya; Ascomycota; Pezizomycotina; Sordariomycetes; Hypocreomycetidae; Hypocreales; Clavicipitaceae; Claviceps. |
| Actin | P53689 | 339 | 14 | 4 | 12 | Cell motility | Xanthophyllomyces dendrorhous | Eukaryota; Fungi; Dikarya; Basidiomycota; Agaricomycotina; Tremellomycetes; Cystofilobasidiales; Cystofilobasidiaceae; Xanthophyllomyces. |
| Autophagy-related protein 18 | Q4P4N1 | 24 | 4 | 2 | 3.8 | Transport; Protein transport | Ustilago maydis 521 | Eukaryota; Fungi; Dikarya; Basidiomycota; Ustilaginomycotina; Ustilaginomycetes; Ustilaginales; Ustilaginaceae; Ustilago. |
| Probable myosin heavy chain ECU04_1000 | Q8SS35 | 92 | 4 | 2 | 1.4 | Cell motility | Encephalitozoon cuniculi GB-M1 | Eukaryota; Fungi; Microsporidia; Unikaryonidae; Encephalitozoon. |
| Elongation factor 2 | Q8SQT7 | 31 | 14 | 4 | 3.6 | Protein biosynthesis | Encephalitozoon cuniculi GB-M1 | Eukaryota; Fungi; Microsporidia; Unikaryonidae; Encephalitozoon. |
| Probable lysine--tRNA ligase, cytoplasmic | C4V8R9 | 28 | 1 | 1 | 2.3 | Protein biosynthesis | Nosema ceranae BRL01 | Eukaryota; Fungi; Microsporidia; Nosematidae; Nosema. |
| Histone H4 | Q9HDF5 | 43 | 2 | 1 | 9.7 | Replication and repair | Mortierella alpina | Eukaryota; Fungi; Fungi incertae sedis; Early diverging fungal lineages; Mortierellomycotina; Mortierellales; Mortierellaceae; Mortierella. |
